# Metabolic Context Regulates Distinct Hypothalamic Transcriptional Responses to Antiaging Interventions

**DOI:** 10.1155/2012/732975

**Published:** 2012-08-27

**Authors:** Alexis M. Stranahan, Bronwen Martin, Wayne Chadwick, Sung-Soo Park, Liyun Wang, Kevin G. Becker, William H. WoodIII, Yongqing Zhang, Stuart Maudsley

**Affiliations:** ^1^Physiology Department, Georgia Health Sciences University, Augusta, GA 30912, USA; ^2^Metabolism Unit, National Institute on Aging Intramural Research Program, Baltimore, MD 21224-6825, USA; ^3^Receptor Pharmacology Unit, National Institute on Aging Intramural Research Program, Baltimore, MD 21224-6825, USA; ^4^Gene Expression and Genomics Unit, National Institute on Aging Intramural Research Program, Baltimore, MD 21224-6825, USA

## Abstract

The hypothalamus is an essential relay in the neural circuitry underlying energy metabolism that needs to continually adapt to changes in the energetic environment. The neuroendocrine control of food intake and energy expenditure is associated with, and likely dependent upon, hypothalamic plasticity. Severe disturbances in energy metabolism, such as those that occur in obesity, are therefore likely to be associated with disruption of hypothalamic transcriptomic plasticity. In this paper, we investigated the effects of two well-characterized antiaging interventions, caloric restriction and voluntary wheel running, in two distinct physiological paradigms, that is, diabetic (*db/db*) and nondiabetic wild-type (*C57/Bl/6*) animals to investigate the contextual sensitivity of hypothalamic transcriptomic responses. We found that, both quantitatively and qualitatively, caloric restriction and physical exercise were associated with distinct transcriptional signatures that differed significantly between diabetic and non-diabetic mice. This suggests that challenges to metabolic homeostasis regulate distinct hypothalamic gene sets in diabetic and non-diabetic animals. A greater understanding of how genetic background contributes to hypothalamic response mechanisms could pave the way for the development of more nuanced therapeutics for the treatment of metabolic disorders that occur in diverse physiological backgrounds.

## 1. Introduction

The hypothalamus plays a pivotal role in mediating the central control of somatic energy metabolism and regulation of higher central nervous function throughout life This ability of the hypothalamus to act as a bridge between central and peripheral systems has made it an important target for the study of age-related decline in metabolism and cognition [1–3]. Hypothalamic neuronal structure and function are dynamically regulated by homeostatic challenges, including feeding, fasting, and physical activity [4, 5]. In addition, genetic animal models with perturbations of somatic energy metabolism have also been shown to exhibit significant alterations in hypothalamic plasticity [6]. The neuroendocrine control of food intake and energy expenditure is associated with, and likely dependent upon, hypothalamic synaptic plasticity. Reinforcing the importance of energy metabolism, both cellular or somatic, in homeostatic stability during lifespan, multiple studies have demonstrated the ability of experimental interventions that either curtail energy intake (caloric restriction) or increase energy expenditure (physical exercise) to ameliorate age-related pathophysiology [7–11]. Severe disturbances in energy metabolism, such as those that occur in obesity that significantly disrupt healthy aging for multiple reasons, are therefore likely to be associated with disruption of hypothalamic neuroplasticity. Maladaptive circuit alterations may also contribute to deleterious increases in hunger and reduced energy expenditure in the context of excessive adiposity. Mice possessing an inactivating mutation of the cognate cytokine receptor for leptin, *db*/*db* mice, are obese, insulin resistant [12], and demonstrate an increased excitatory drive onto orexigenic neuropeptide Y-expressing hypothalamic neurons [13]. Leptin also contributes to the development of hypothalamic responsiveness and integration of food intake and metabolism [6] and also promotes morphological plasticity among hypothalamic neurons in the adult brain [14]. Various molecular transcriptomic signatures that respond to changes in energy intake and expenditure have now been characterized [15–18]. Consistent with leptin's role in energy metabolism, several leptin-responsive target genes have been identified in the hypothalamus [18, 19]. Challenges to metabolic homeostasis, such as caloric restriction (CR [20]) and voluntary wheel running [21, 22] are also associated with changes in hypothalamic gene transcription. Both reduced energy intake, through CR, and increased energy expenditure, through wheel running, have been associated with neuroendocrine alterations that are likely to be accompanied by dynamic alterations in hypothalamic neuroplasticity [4, 23]. However, a comprehensive picture of the transcriptional alterations that occur in both diabetic and nondiabetic animals in response to complex energetic challenges that prolong life and health span in multiple species, such as CR or wheel running, has yet to be determined. Such a question is important considering the multiple alterations in hormone levels, feedback responses and receptor functionality that occur in distinct physiological contexts, as these may disrupt the efficacy of the applied antiaging interventions. In order to assess global alterations in gene expression patterns following energetic challenges, we compared hypothalamic transcriptional profiles, following running or CR, in a diabetic context, that is, leptin receptor-deficient (*db/db)* mice compared to non-diabetic C57Bl/6 controls. Our study therefore is aimed at appreciating how the prevailing health context background can affect the applied therapeutic antiaging interventions of CR or exercise.

## 2. Materials and Methods

### 2.1. Animal Husbandry and Activity Monitoring

 Animal care and experimental procedures followed NIH guidelines and were approved by the National Institute on Aging Animal Care and Use Committee (293-LNS-2010). For microarray analyses, male leptin receptor mutant (*db/db*, *n* = 24) mice, bred on a C57Bl/6 background, were purchased from The Jackson Laboratories. Age-matched male C57Bl/6 mice (wild type, *n* = 24) were used as controls. To validate the cycling conditions used to detect leptin mRNA in the brain, hippocampal tissue from (*n* = 3) *ob/ob* mice and (*n* = 3) wild-type mice was obtained and analyzed. Animals were one month old at the start of experiments. Mice from each genotype were kept in individual cages containing a running wheel equipped with an automated, computerized monitoring system. The running wheel was continuously available to the mice. The number of wheel rotations per day for each mouse was continuously recorded using MedSci Behavior monitoring software (Columbus Instruments, Columbus, OH). An additional cohort of (*n* = 6) C57Bl/6 male mice and (*n* = 8) *db/db* mice was used for *in situ* hybridization experiments, to confirm microarray data. During the initial two weeks of the experiment, all mice were fed *ad libitum*, and food weights were recorded daily by experimenters. *Ad libitum* feeding levels were initially monitored for control and *db/db* animals, and then a diet that would supply sixty percent of their individual mean food intake was applied to generate forty percent caloric restriction (40% CR). This level of restricted feeding was chosen based on previous experiments [15, 16, 24]. The vivarium was maintained on a twelve hour light/dark cycle; all mice assigned to the CR diet were fed once daily at the onset of the dark period (18:00 hrs). Body weights were recorded on a weekly basis.

### 2.2. Hypothalamic Tissue Preparation

 For *in situ* hybridization, mice were deeply anesthetized with Isoflurane, then perfused with 4% paraformaldehyde in phosphate buffer. Brains were postfixed in 4% paraformaldehyde with progressively increasing concentrations of sucrose, then stored at −80°C prior to sectioning. Hemibrains were sectioned at 40 *μ*m in the coronal plane using a freezing microtome (Michrom M450, Fisher Scientific, Pittsburgh, PA). Sections were collected in a 1 : 6 series and stored in 4% paraformaldehyde. For microarray analysis and semiquantitative RT PCR, mice were anesthetized with isoflurane as described above, decapitated, and the brains were removed for hypothalamic dissection on ice. Dissected hypothalamic samples were frozen on dry ice and stored at −80°C prior to RNA extraction. Trunk blood was collected for serum analyses of metabolic and stress hormones, lipids; and ketone bodies and was stored at −80°C until used.

### 2.3. Circulating Metabolic Hormone and Lipid Measurements

For measurement of fasting glucose levels, food was removed from the cages of the mice on the *ad libitum* diet, and the daily allotment of food was withheld from mice on the 40% CR diet, the evening before glucose testing (17:00 hrs). The following morning, animals were briefly restrained, and glucose levels were measured following tail nick using a Therasense handheld analyzer (Therasense, Alameda, CA). Cholesterol and triglycerides were measured from trunk blood (after euthanization) using a Roche Cobas Fara II robotic chemical analyzer according to the manufacturer's specifications. All reagents for these analyses were purchased from Wako Diagnostics (Richmond, VA). Total cholesterol levels were determined using a kit (catalog no. 439-17501), as were triglyceride levels (catalog no. 461-08992). Insulin and leptin concentrations in serum samples were determined by ELISA (Crystal Chem., Inc., Downers Grove, IL). These assays were performed according to the manufacturer's instructions. Briefly, a microtiter plate coated with mouse antiinsulin or guinea pig antileptin antibody was washed three times with wash buffer (50 mM Tris-buffered saline (TBS) containing Tween 20). Five microliters of diluted standards and serum samples were added to wells in duplicate. Detection antibodies conjugated to the appropriate species were applied, and the plate was sealed and incubated for 2 hours while shaking. The wells were then washed and the enzyme solution was incubated for 30 minutes. After washing, wells were reacted with substrate solution (o-phenylenediamine). Once the color developed sufficiently (15 minutes), stop solution (1 N sulfuric acid) was added, and the plate was read at 490 nm on an automatic plate reader (Perkin Elmer HTS 7000 Plus Bio Assay Reader, Perkin Elmer, Waltham, MA).

### 2.4. Circulating Corticosterone Measurements

 Corticosterone levels were measured using a commercially available Radioimmunoassay (RIA) kit (Diagnostic Products Corp., Los Angeles, CA) according to the manufacturer's instructions. Serum was separated from trunk blood by centrifugation at 14,000 rpm for two minutes. Serum samples were stored at −80°C prior to analysis. Samples and corticosterone standards were thawed at room temperature and added to antibody-coated tubes in duplicate. 1.0 mL of (I^125^-) labeled corticosterone was added, and each tube was vortexed before incubation for two hours at room temperature. Tubes were then decanted and counted using a Packard Cobra (5010) gamma counter.

### 2.5. Illumina Oligonucleotide Microarray

 RNA isolation was carried out using the Qiagen RNeasy Mini Kit for animal tissues (Qiagen, Inc., Valencia, CA). In short, frozen tissues were cut into small pieces and allowed to thaw at 4°C in RLT lysis buffer (Qiagen). Tissue sections were disrupted using a Mini-Beadbeater-8 and 1.0 mm glass beads (BioSpec Inc., Bartlesville, OK). Tissue samples were then centrifuged, the supernatant transferred to a second tube and centrifuged again for cell debris clarification. The supernatant was added to 95% ethanol, mixed, and added to the binding columns. The columns were centrifuged, washed several times and the bound RNA was eluted using water. The RNA quality and quantity was checked using an Agilent 2100 bioanalyzer and RNA 6000 nano-chips. Total RNA was used to generate biotin-labeled cRNA using the Illumina TotalPrep RNA Amplification Kit (Ambion; Austin, TX). Briefly, 0.5 *μ*g of total RNA was first converted into single-stranded cDNA with reverse transcriptase using an oligo-dT primer containing the T7 RNA polymerase promoter site and then copied to produce double-stranded cDNA molecules. Double-stranded cDNA was used in an overnight *in vitro* transcription reaction where single-stranded RNA (cRNA) was generated and labeled by incorporation of biotin-16-UTP. A total of 0.75 *μ*g of biotin-labeled cRNA was hybridized at 58°C for 16 hours to Illumina's Sentrix MouseRef-8 Expression BeadChips (Illumina, San Diego, CA). Arrays were then washed, blocked and the labeled cRNA was detected by staining with streptavidin-Cy3. The arrays were scanned using an Illumina BeadStation 500X Genetic Analysis Systems scanner, and the image data extracted using the Illumina BeadStudio software, version 3.0.

### 2.6. Microarray Data Analysis

 Microarray data were analyzed using DIANE 6.0, a spreadsheet-based microarray analysis program based on the SAS JMP7.0 system. Raw microarray data were subjected to filtering and *Z-*normalization and tested for significant changes as described previously [25]. Briefly, initial filtering identified genes with *Z*-ratio ≥ 1.50, with the *Z*-ratio derived from the difference between the averages of the observed gene *Z* scores, divided by the standard deviation of all of the differences for that particular comparison. Genes were then refined by calculating the false discovery rate (FDR), which controls for the expected proportion of falsely rejected hypotheses, and including only those genes with FDR < 0.05. These data were further analyzed using a 2 × 3 ANOVA design with significance set at *P* < 0.05. The ANOVA design compared across genotype (*db/db* versus C57Bl/6) and environmental condition (sedentary/*ad libitum* versus sedentary/caloric restriction versus runner/*ad libitum*). This allowed us to identify transcripts that differed in their intensity across the various conditions in normal and leptin receptor-deficient mice. For functional genomic pathway analysis, specific significantly regulated gene lists were analyzed using Ingenuity version 8.6. The reference gene set was defined through the Ingenuity knowledge base (Genes Only). Inclusion in a pathway was defined as having a direct or indirect relationship with that pathway. For each pathway identified, the inclusion of at least two genes was required from the input dataset with a probability value of < 0.05. The resultant data were also filtered by considering only those molecules and relationships, where species = mouse, and tissue = nervous system.

### 2.7. *In Situ* Hybridization

 Generation of riboprobes for *in situ* hybridization was performed as described previously [26]. Probe sequences were as follows: for Pias2, right primer agtgttactgggctttgctg, left primer tctcaaaggtgggcttagtg; for Slc17a6, right primer tggcacatgtcatcctacag, left primer ccctccctttacaagctctc. Target genes were selected on the basis of significance derived from the microarray analysis, and on selective expression enrichment in the hypothalamus based on interrogation of the Allen Brain Atlas [27]. Leptin primer sequences were as follows: right primer tgtccaagatggaccagactc, left primer actggtctgaggcagggagca. Animal tissue for *in situ* hybridization was processed in separate runs that were balanced to include equal numbers of animals from each experimental condition. *In situ* hybridization was carried out as described previously [26]. Densitometric analysis was performed upon scanned images generated using a Typhoon Phosphorimager (GE Healthcare, Piscataway, NJ). Regions of interest from matched sections were user defined with ImageQuant (GE Healthcare) by an experimenter blind to the specific experimental group conditions. Five bilateral sections were averaged to obtain a single score for each animal. In order to validate our microarray studies, which measured gene expression changes across the whole hypothalamus, we sampled across all hypothalamic nuclei for *in situ* hybridization analyses. Sampling extended from the lateral preoptic area rostrally (Bregma +0.14 mm) to the lateral hypothalamic area caudally (Bregma −2.80 mm) according to the atlas of Paxinos and Franklin [28]. Anatomical sampling was balanced across groups to include one section containing the lateral preoptic area (Bregma +0.14 mm to −0.22 mm); one section spanning the anterior hypothalamic area, medial preoptic nucleus, suprachiasmatic nucleus, paraventricular nucleus, and lateral hypothalamus (Bregma −0.34 mm to Bregma −1.06 mm); one section containing paraventricular nucleus, ventromedial hypothalamus, arcuate nucleus, dorsomedial hypothalamic nucleus, and lateral hypothalamus (Bregma −1.22 mm to Bregma −1.70 mm); one section containing the posterior hypothalamic area, lateral hypothalamus, dorsomedial hypothalamus, ventromedial hypothalamus, and arcuate nucleus (Bregma −1.82 mm to Bregma −2.30 mm); one section containing the posterior hypothalamic area, lateral hypothalamus, and arcuate nucleus (Bregma −2.46 mm to Bregma −2.80 mm).

### 2.8. Semiquantitative Real-Time PCR

Primer sequences and expected product sizes are shown in Table S1 Supplementary Matrial available online at doi: 10.1155/2012/732975. For genes with multiple transcript variants (IGF1 and NTRK3), Biology Workbench (version 3.2) was used to align the sequences, and primers were generated based on the consensus sequence. RNA sample quality was assessed through gel electrophoresis and spectrophotometric analysis. Total RNA samples were also treated with 1.0 *μ*L DNase I (2 U/*μ*L; Ambion, Austin, TX) to remove any genomic DNA. RNA samples (0.5 *μ*g) were converted to cDNA using SuperScript III reverse transcriptase (Invitrogen, Carlsbad, CA) with oligo-dT and random hexamers. Samples were also run in the absence of SuperScript III to evaluate the potential for genomic DNA contamination. cDNA samples (4.0 *μ*L) were added to a 50 *μ*L PCR amplification using Platinum PCR Supermix (Invitrogen, Carlsbad, CA), with gene-specific primers (Table S1). The linear range of PCR amplification for each primer set was determined in pilot experiments (25–35 cycles). Cycling conditions were a single 5-minute step at 95°C, followed by the appropriate number of amplification cycles, with annealing at 58°C. For leptin detection, PCR products were verified with DdeI restriction enzyme digest as described on The Jackson Laboratories website (http://jaxmice.jax.org/protocols). PCR products were run in a 1.2% agarose gel containing 0.5 *μ*g/mL ethidium bromide at 100 V for one hour. Images were acquired under ultraviolet light using a BioRad ChemiDoc molecular imaging system (BioRad, Hercules, CA). Band intensities were quantified using NIH ImageJ (http://rsbweb.nih.gov/ij/) and expressed relative to the band intensity for glyceraldehyde-3-phosphate dehydrogenase (GAPDH).

### 2.9. Statistical Analysis

*In situ* hybridization and semiquantitative PCR data were compared across genotypes using bidirectional Student's *t*-tests (GraphPad Prism v.5, La Jolla, CA). Endocrine data were analyzed using one-way ANOVA with planned *post hoc* comparisons (sedentary C57Bl/6 mice on the *ad libitum* diet compared to all other groups; sedentary *db/db* mice on the *ad libitum* diet compared to *db/db* runners; sedentary *db/db* mice on the *ad libitum* diet compared to *db/db* mice on CR). The same *post hoc* planned comparisons were applied to the data on body weight gain and food intake following 2 × 3 repeated measures ANOVA using SPSS version 18. For all analyses, statistical significance was set at *P* < 0.05.

## 3. Results

### 3.1. Body Weight and Food Intake Alterations following Energetic Challenges

**Figure 1:**
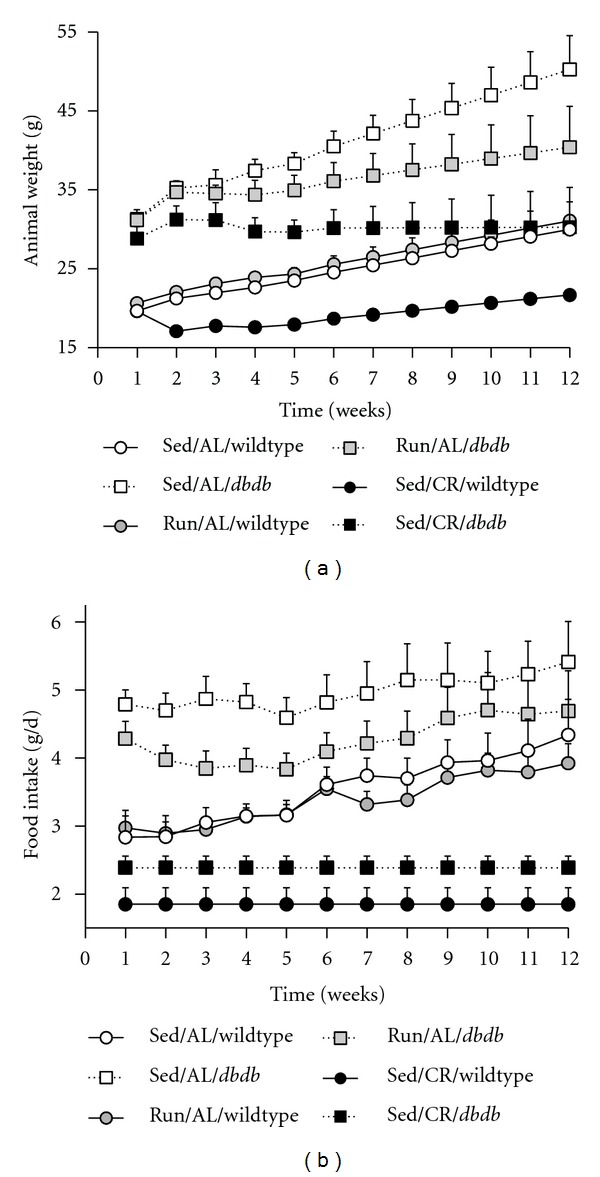
Caloric restriction and wheel running alter body weight gain and food intake in C57Bl/6 and leptin receptor-deficient mice. (a) C57Bl/6 mice maintained on 40% caloric restriction (CR) gain weight more slowly than wild-type mice on the *ad libitum* (AL) diet. *db/db* mice maintained on CR from one month of age did not significantly gain weight over the subsequent twelve weeks of the experiment, and voluntary wheel running decelerates body weight gain in *db/db* mice. (b) Running transiently suppresses food intake in *db/db* mice. Data were analyzed with 2 × 3 repeated measures ANOVA with Tukey's *post hoc* and significance set at *P* < 0.05. Error bars represent SEM.

 In agreement with previous reports, the CR paradigm reduced body weight in both C57Bl/6 (WT) and *db/db* mice (Figure 1(a); *F*_5,35_ = 16.84, *P* = 0.0001) [29, 30]. In *db/db* mice, but not WT mice, voluntary wheel running also reduced body weight gain (*F*_5,35_ = 2.94, *P* = 0.02). Effects on body weight occurred in the context of attenuated hyperphagia in *db/db* runners (*F*_5,35_ = 3.71, *P* = 0.01; Figure 1(b)), without any change in food intake in WT runners.

### 3.2. Alterations in Metabolic Hormone, Stress Hormone, and Lipid Profiles following Running or Caloric Restriction

**Figure 2:**
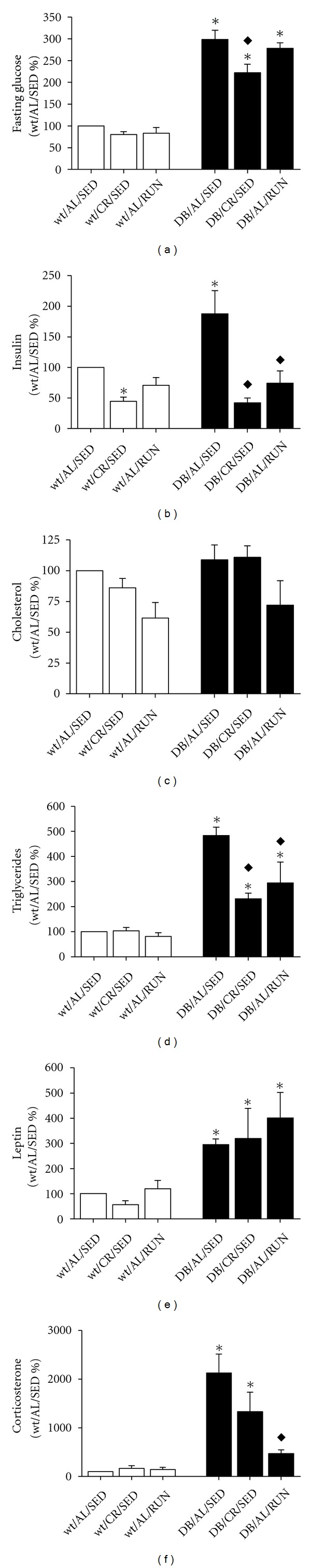
Endocrine changes in C57Bl/6 and *db/db* mice following running or caloric restriction. For all graphs, asterisk (*) indicates significance at *P* < 0.05 relative to sedentary C57Bl/6 mice fed *ad libitum*. Black diamonds (♦) represent significance at *P* < 0.05 relative to sedentary *db/db* mice fed *ad libitum*. (a) Caloric restriction (CR) attenuates fasting hyperglycemia in *db/db* mice. (b) CR lowers circulating insulin concentrations in C57Bl/6 mice, and both running and CR reinstate normal insulin levels in *db/db* mice. (c) Although there is a trend towards reduced total cholesterol in runners, this did not reach statistical significance. (d) Serum triglycerides are elevated in *db/db* mice, but this elevation can be ameliorated following running or CR. (e) Serum leptin levels are increased in *db/db* mice across all conditions. (f) Running attenuates the elevated corticosterone levels observed in *db/db* mice. Abbreviations: wt = C57Bl/6; AL = *ad libitum*; CR = caloric restriction; RUN = wheel running; DB = *db/db* mice.

Fasting glucose levels were significantly reduced following CR in *db/db* mice (Figure 2(a); *F*_5,34_ = 45.51, *P* = 0.0001). In WT mice, CR lowered circulating insulin levels, and in *db/db* mice, both running and CR significantly attenuated hyperinsulinemia (Figure 2(b); *F*_5,42_ = 4.65, *P* = 0.002). A strong trend towards reduced total cholesterol in runners of both genotypes was also evident (Figure 2(c); *F*_5,31_ = 2.48, *P* = 0.057). The significantly elevated serum triglycerides in *db/db* mice (compared to WT) were significantly attenuated following running or CR (Figure 2(d); *F*_5,31_ = 24.54, *P* = 0.0001). Additionally, *db/db* mice demonstrated persistently elevated leptin levels, relative to WT mice (Figure 2(e); *F*_5,29_ = 4.61, *P* = 0.004). In agreement with previous studies performed in the Zucker rat model [31], running reversed the elevated corticosterone levels observed in *db/db* mice (Figure 2(f); *F*_5,30_ = 11.81, *P* = 0.0001).

### 3.3. Differential Hypothalamic Gene Expression Patterns in *db/db* Mice, Compared to WT Mice, under Ad Libitum, Sedentary Conditions

**Figure 3:**
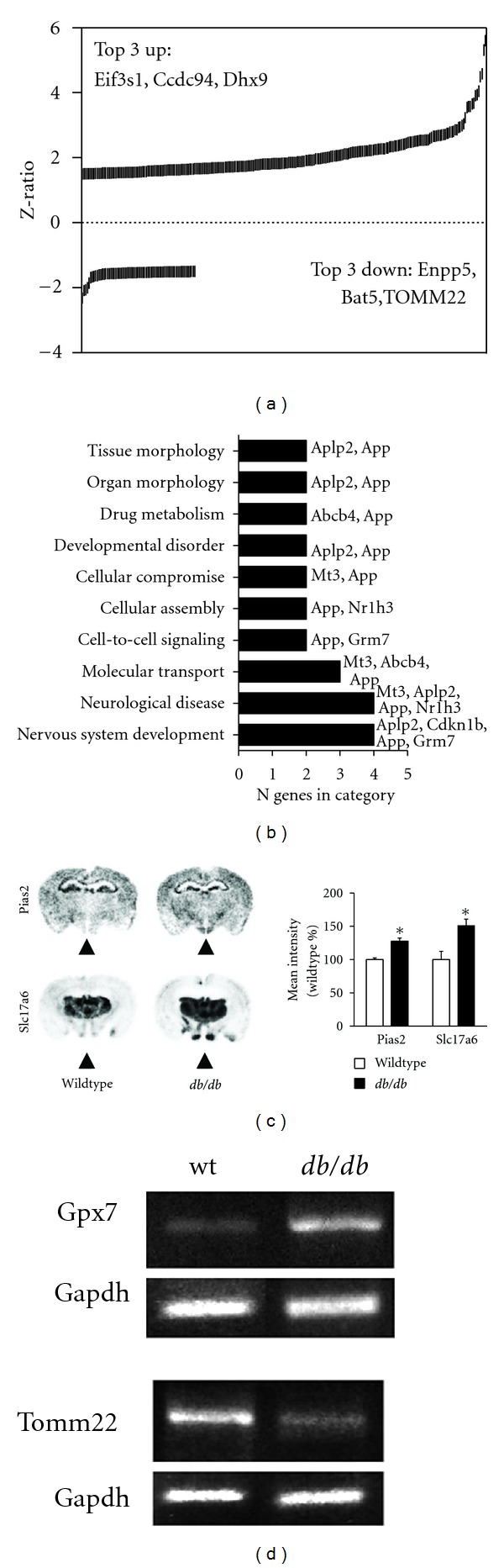
Differences in hypothalamic gene expression and pathway recruitment between C57Bl/6 and *db/db* mice under ad libitum, sedentary conditions. (a) Graph of Z-ratios showing that differences in gene expression between C57Bl/6 and *db/db* mice are primarily attributable to transcriptional upregulation, as opposed to down-regulation. Abbreviations: Eif3s1, eukaryotic translation initiation factor 3, subunit J; Ccdc94, coiled-coil domain containing 94; Dhx9, DEAH (Asp-Glu-Ala-His) box polypeptide 9; Enpp5, ectonucleotide pyrophosphatase/phosphodiesterase 5; Bat5, HLA-B associated transcript 5; Tomm22, translocase of outer mitochondrial membrane 22. (b) Pathways that differ between wild-type and db/db mice under *ad libitum*, sedentary conditions. Aplp2, amyloid beta (A4) precursor-like protein 2; App, amyloid beta precursor protein; Abcb4, ATP-binding cassette, subfamily B member 4; Mt3, metallothionein 3; Nr1h3, nuclear receptor subfamily 1, group H, member 3; Grm7, glutamate receptor, metabotropic 7; Cdkn1b, cyclin-dependent kinase inhibitor 1B. (c) Pias2 and Slc17a6 expression in C57Bl/6 and *db/db* mice under *ad libitum*, sedentary conditions. Arrowheads indicate the hypothalamic nuclei, which were traced over the rostrocaudal extent of the hypothalamus to generate an average index of gene expression that would be directly comparable to the microarray analysis conducted in whole-hypothalamus RNA extracts. Pias2, protein inhibitor of activated STAT 2; Slc17a6, solute carrier family 17 member 6. Associated graph indicating densitometric results of the expression analysis. Consistent with the microarray results, both Pias2 and Slc17a6 were significantly upregulated in the hypothalamus of *db/db* mice. Asterisk (*) indicates statistical significance at *P* < 0.05 following bidirectional student's *t*-tests. (d) Further validation through semiquantitative PCR reveals that, as shown by the microarray, Gpx7 mRNA is increased and Tomm22 mRNA is reduced in the hypothalamus of *db/db* mice.

 To establish a baseline comparison for hypothalamic gene transcripts that differ significantly in their expression between *db/db *and WT mice under *ad libitum* feeding (simplified to “*ad libitum*”) and sedentary (nonrunning) conditions, we assessed global hypothalamic gene expression using an Illumina bead microarray. 244 genes differed significantly in their expression between sedentary *db/db* and WT mice, the majority of which were upregulated (185 genes upregulated; Figure 3(a), Table S2). Among upregulated genes, transcriptional regulators were prominently represented, for example, eukaryotic translation initiation factor 3, subunit 1 (Eif3s1), coiled-coil domain containing 94 (Ccdc94), and DEAH (Asp-Glu-Ala-His) box polypeptide 9 (Dhx9). Among significantly downregulated transcripts (in *db/db* compared to WT), a strong representation of enzymes, membrane proteins, and mitochondrial-related gene transcripts was evident, for example, ectonucleotide pyrophosphatase/phosphodiesterase 5 (Enpp5), HLA-B-associated transcript 5 (Bat5), and translocase of outer mitochondrial membrane 22 (Tomm22). Statistical signaling pathway analysis (Ingenuity Pathway Analysis: IPA) of the gene transcripts differentially expressed between the *db/db *and WT mice under *ad libitum*, sedentary conditions were linked primarily with “nervous system development,*”* “neurological disease,*”* and “molecular transport” (Figure 3(b)). The specific genes associated with the regulation of these highest probability-scoring signaling pathways included amyloid beta precursor-like protein 2 (Aplp2), cyclin-dependent kinase inhibitor 1B (Cdkn1b), amyloid precursor protein (App), and metabotropic glutamate receptor type 7 (Grm7). Additionally functional gene networks, created using IPA algorithms, from this dataset included pathways linked to “cell-to-cell signaling,” as well as “tissue and organ morphology.”

To validate multiple aspects of our hypothalamic microarray analysis, we characterized *db/db*-related differences in gene expression using *in situ* hybridization and semiquantitative reverse transcriptase PCR (RT-PCR). We measured the expression of four chosen transcripts differentially expressed in *db/db* mice compared to WT. From our array data both protein inhibitor of activated STAT2 (Pias2) and solute carrier family 17 (sodium-dependent inorganic phosphate cotransporter) member 6 (Slc17a6) were significantly upregulated in the hypothalamus of *db/db *mice compared to WT. *In situ* hybridization of these genes confirmed their expression potentiation in *db/db* mice (Figures 3(c) and 3(d)). With RT-PCR, expression of glutathione peroxidase 7 (Gpx7) was elevated, and the expression of translocase of outer mitochondrial membrane 22 (Tomm22) was found to be reduced, both of which were consistent with the microarray results (Table S2).

### 3.4. Regulation of Hypothalamic Transcription Patterns by Running Activity in WT Mice

**Figure 4:**
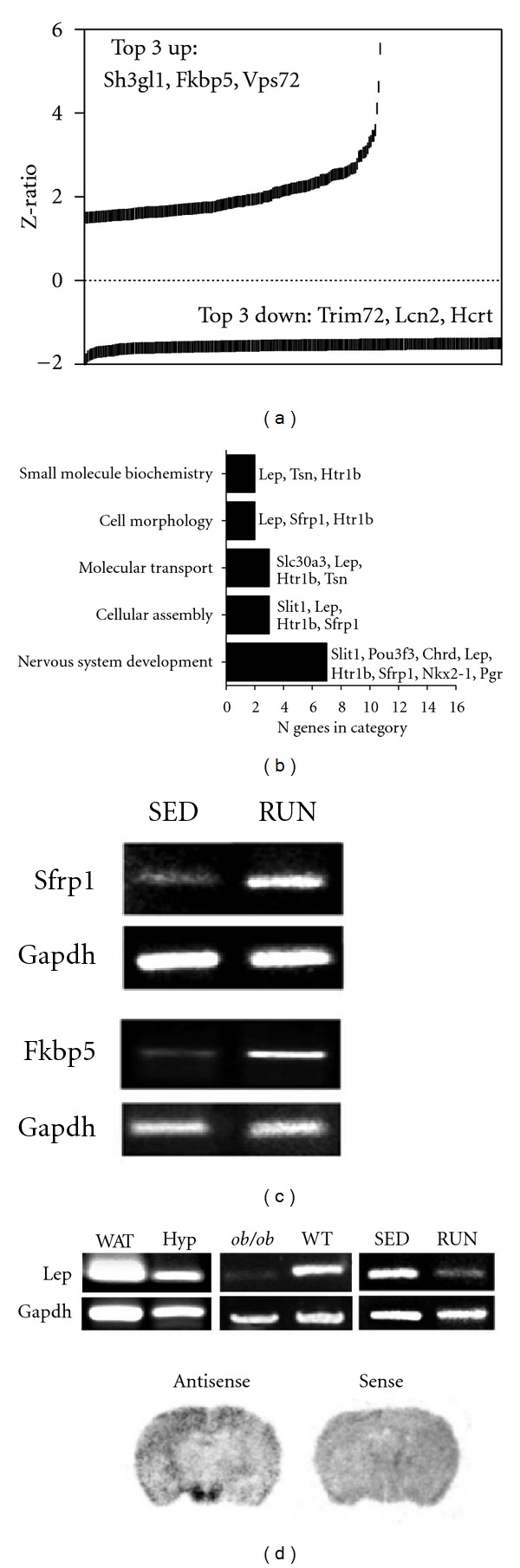
Wheel running influences hypothalamic gene expression and pathway activation in C57Bl/6 mice. (a) *Z*-ratios for gene transcripts up- or downregulated following voluntary wheel running in C57Bl/6 mice. Fkbp5, FK506 binding protein 5; Vps72, vacuolar protein sorting 72; Sh3gl1, SH3-domain GRB2-like 1; Lcn2, lipocalin 2; Hcrt, hypocretin; Trim72, tripartite motif-containing 72. (b), Pathways responsive to running in the hypothalamus of C57Bl/6 mice. Abbreviations: Lep, leptin; Htr1b, 5-hydroxytryptamine receptor 1B; Tsn, translin; Sfrp1, secreted frizzled-related protein 1; Slc30a3, solute carrier family 30, member 3; Slit1, slit homolog 1; Pou3f3, POU domain, class 3, transcription factor 3; Chrd, chordin; Nkx2-1, NK2 homeobox 1; Pgr, progesterone receptor. (c), PCR validation of running-induced transcriptional alterations in the hypothalamus of wild-type mice. (d) Leptin mRNA was detected in the hypothalamus samples using microarray, PCR, and *in situ* hybridization techniques. White adipose tissue (WAT) expresses leptin mRNA at higher levels than hypothalamus (Hyp) by PCR; *ob/ob* mice lack leptin bands at 155 bp in the hypothalamus following restriction enzyme digest, while wild-type (wt) controls express leptin mRNA. As shown by the microarray, running reduced endogenous leptin mRNA expression in the hypothalamus. *In situ* hybridization techniques were also used to detect endogenous leptin mRNA expression in the hypothalamus of sedentary wild-type mice.

 Experimental mice often voluntarily run long distances with an available running wheel. The WT mice employed in this study ran 6.68 ± 1.45 km/24 hr. When comparing the gene transcripts differentially regulated by running in WT mice compared to their WT sedentary counterparts, we found that the majority of significantly altered genes were downregulated (*∼*58%; Figure 4(a); Table S3) in response to running. This profound increase in activity potentiated the expression of transcripts involved with neuronal development and differentiation (FK506 binding protein 5, Fkbp5: [32]; SH3-domain GRB2-like 1, Sh3gl1: [33]; secreted frizzled-related protein 1, Sfrp1: [34]) and chromatin remodelling (vacuolar protein sorting 72, Vps72: [35]). Running activity, in contrast, depressed hypothalamic expression of transcripts related to appetite and metabolism (hypocretin/orexin, Hcrt: [36]; lipocalin 2, Lcn2 [37]; leptin, Lep [38]) as well as insulin receptor signaling (tripartite motif-containing 72, Trim72 [39]). Alterations in Sfrp1 and Fkbp5 and mRNA expression were confirmed through RT-PCR (Figures 4(b), 4(c)). Both PCR and *in situ* hybridization were used to detect leptin mRNA in the hypothalamus (Figure 4(d)). Hypothalamic leptin mRNA expression was detectable in sedentary mice using *in situ* hybridization (Figure 4(d)), consistent with previous reports demonstrating endogenous leptin mRNA expression in the hypothalamus [29, 30]. We compared hypothalamic leptin mRNA in hypothalamus with mRNA in white adipose tissue (WAT). Leptin mRNA expression was lower in the hypothalamus than WAT and as a control was absent in ob/ob hypothalamus (Figure 4(d)). Confirming our array data we found, with RT-PCR, that indeed hypothalamic leptin mRNA was reduced with running (Figure 4(d)). Investigating the signaling pathways populated by the differential running versus sedentary gene-set in WT mice. a strong neurodevelopmental phenotype was observed (Figure 4(b)). Running influenced the expression of numerous genes associated with *“nervous system development,”* “molecular transport,” and “cell morphology.” Genes implicated in nervous system development and function included leptin and the murine homolog of *Drosophila* Slit1. The gene encoding POU domain, class 3, transcription factor 3 (Pou3f3), was also represented in this category, as was the presynaptic modulator chordin (Chrd [40]). Serotonin receptor 1b (Htr1b), Sfrp1, NK2 homeobox 1 (Nkx2-1), and the progesterone receptor (Pgr) were also included as components of the “*nervous system development*” pathway regulated by running in WT mice.

### 3.5. Regulation of Hypothalamic Transcription Patterns by Caloric Restriction in WT Mice

**Figure 5:**
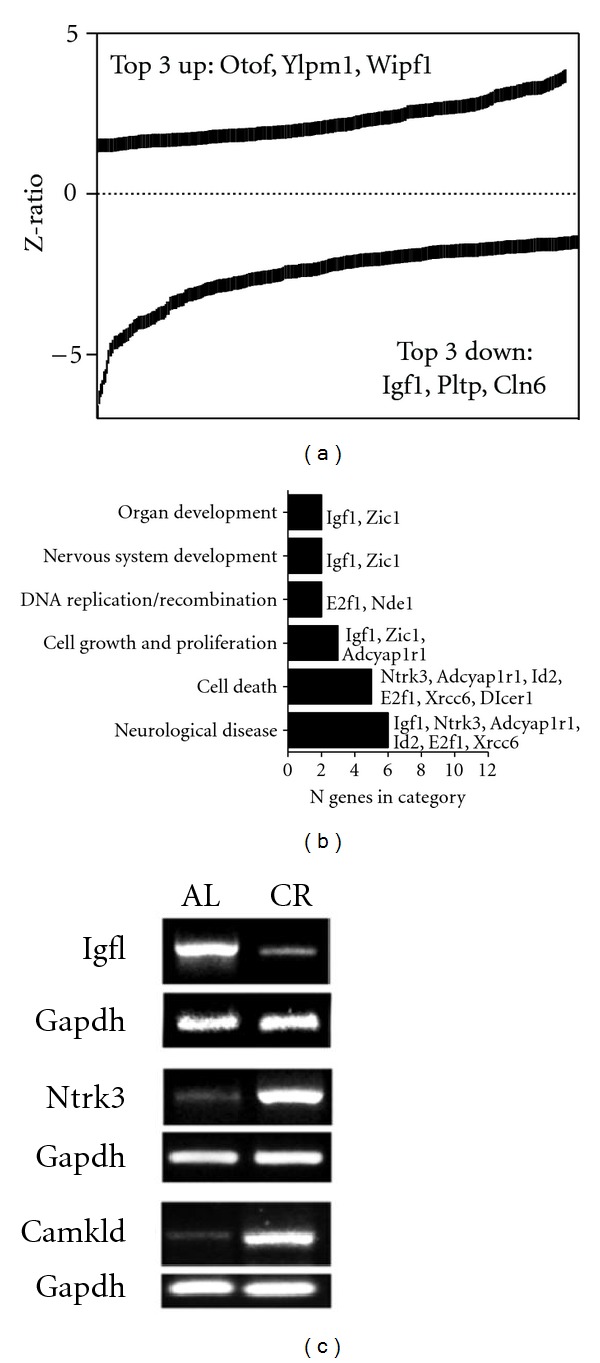
CR influences hypothalamic gene expression and pathway recruitment in wild-type mice. (a) *Z*-ratios for genes that were up- or down-regulated following CR in wild-type mice. Otof, otoferlin; Ylpm1, YLP motif containing 1; Wipf1, WAS/WASL interacting protein family, member 1; Igf1, insulin-like growth factor 1; Pltp, phospholipid transfer protein; Cln6, ceroid-lipofuscinosis, neuronal 6. (b) Pathway analysis of genes responsive to CR in wild-type mice. Abbreviations: Zic1, zinc finger protein of the cerebellum 1; E2f1, E2F transcription factor 1; Nde1, nuclear distribution gene E homolog 1; Adcyap1r1, adenylate cyclase activating polypeptide 1 receptor 1; Ntrk3, neurotrophic tyrosine kinase, receptor, type 3; Id2, inhibitor of DNA binding 2; Xrcc6, X-ray repair in Chinese hamster cells 6; Dicer1, Dicer1 homolog. (c) PCR validation of changes in gene expression following CR in wild-type mice.

Following CR implementation in WT mice, 521 genes were significantly altered in the hypothalamus compared to *ad libitum*-fed WT controls. Similar numbers of genes were significantly up- or downregulated (269 up-regulated, 225 downregulated; Figure 5(a), Table S4). Many of the upregulated transcripts are involved in regulating neurotransmission (otoferlin, Otof [41]: Wiskott-Aldrich syndrome protein interacting protein family 1, Wipf1 [42]; neurotrophin receptor tyrosine kinase 3, Ntrk3 [43]; calcium/calmodulin kinase 1D, CamK1d [44]) and differentiation YLP motif containing 1 (Ylpm1 [45]). CR in the WT animals, however, caused profound suppression of multiple energy-modulatory factors including insulin-like growth factor 1 (Igf1), phospholipid transfer protein (Pltp), and neuronal ceroid lipofuscinosis 6 (Cln6), which were significantly downregulated following CR. Changes in Igf1, Ntrk3, and CamK1d were further validated by RT-PCR (Figures 5(b), 5(c)). Band intensities for each of the validated genes confirmed the differences shown by the microarray results.

The functional pathways that were significantly altered in the C57Bl/6 mice following CR included “neurological disease,” “cell death,” and “cellular growth and proliferation” (Figure 5(b)). Ntrk3, adenylate cyclase activating polypeptide 1 receptor 1 (Adcyap1r1), E2F transcription factor 1 (E2f1) and X-ray repair in Chinese hamster cells 6 (Xrcc6) were represented within the “neurological disease” pathway. Reinforcing its pluripotent role in both endocrine and neuronal health status, Igf1 was represented across multiple pathways, including “nervous system development”, “cell growth and proliferation”, and “neurological disease” (Figure 5(b)).

### 3.6. Regulation of Hypothalamic Transcription Patterns by Running Activity in *db/db* Mice

**Figure 6:**
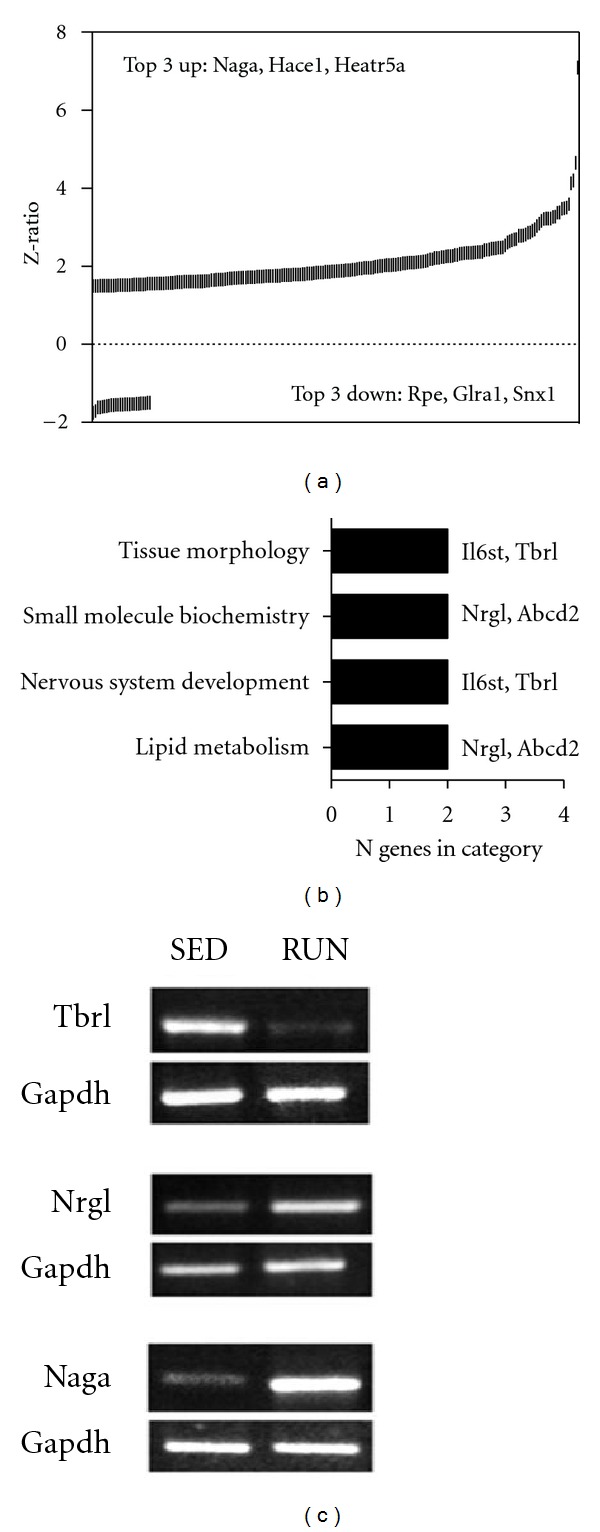
*db/db* mice respond to running with a distinct transcriptional profile. (a) Leptin receptor-deficient *db/db *mice exhibit transcriptional upregulation, indicated by positive *Z*-ratios, following twelve weeks of voluntary wheel running. Rpe, ribulose-5-phosphate-3-epimerase; Glra1, glycine receptor, alpha 1 subunit; Snx1, sorting nexin 1; Naga, N-acetyl galactosaminidase, alpha; Hace1, HECT domain and ankyrin repeat containing, E3 ubiquitin protein ligase 1; Heatr5a, HEAT repeat containing 5A. (b) Pathway analysis of genes regulated by voluntary exercise in *db/db* mice. Abbreviations: Il6st, interleukin 6 signal transducer; Tbr1, T-box brain gene 1; Nrg1, neuregulin 1; Abcd2, ATP-binding cassette, sub-family D, member 2. (c) PCR validation of differences in gene expression following running in *db/db* mice.

Diabetic *db/db* mice ran considerably less than their WT counterparts (0.33 ± 0.08 km/24 hr). Consistent with this reduced drive for voluntary activity, considerably fewer hypothalamic genes (compared to WT mice) were differentially expressed following running in *db/db *mice. 240 genes met our criteria for differential expression following running (compared to sedentary *db/db* mice) in the *db/db *mouse hypothalamus. Of those genes, the majority (*∼*89%) were upregulated (Figure 6(a); Table S5). Significantly upregulated genes included transcripts associated with neuronal protein modification involving lysosomal or ubiquitin function (N-acetylgalactosaminidase alpha, Naga [46]: HECT domain and ankyrin repeat containing, Hace1 [47]), and cell growth and cycle progression (neuregulin, Nrg1 [48]: HEAT repeat containing 5A, Heatr5a [49]). Among the down-regulated transcripts several are prominent controllers of neuronal excitability and development (glycine receptor alpha 1, Glra1 [50]: T-box brain gene 1, Tbr1 [51]), receptor expression stabilization (sorting nexin 1, Snx1 [52]), and pentose-phosphate metabolism (ribulose-5-phosphate-3-epimerase, Rpe [53]). Changes in Naga, Tbr1 and Nrg1 were further confirmed by RT-PCR (Figures 6 (b), 6 (c)). Band intensities for each of these genes were compared across *db/db* mice that ran or were sedentary, and the differences in band intensities were in accordance with the microarray results.

Functional gene pathway analysis of the genes significantly altered in *db/db* mice after running, revealed that four major functional categories, “nervous system development,” “small molecule biochemistry,” “tissue morphology,” and “lipid metabolism” were significantly represented (Figure 6(b)). Two genes associated with “lipid metabolism” and “small molecule biochemistry,” ATP-binding cassette, sub-family D, member 2 (Abcd2) and Nrg1 were influenced by running in *db/db* mice. Two genes implicated in “nervous system development and function” and “tissue morphology”, T-box brain gene 1 (Tbr1) and interleukin 6 signal transducer (Il6st), were also differentially regulated following running in *db/db* mice.

### 3.7. Regulation of Hypothalamic Transcription Patterns by Caloric Restriction in *db/db* Mice

**Figure 7:**
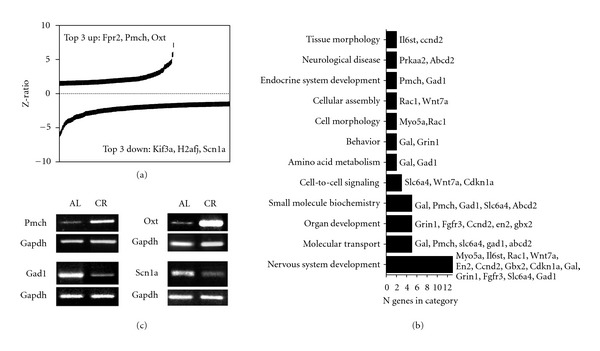
CR activates a diverse set of transcripts and pathways in *db/db *mice. (a) *Z*-ratios for genes that were up- or downregulated following CR in *db/db* mice. Fpr2, formyl peptide receptor 2; Pmch, pro-melanin-concentrating hormone; Oxt, oxytocin; Kif2a, kinesin family member 2A; H2afj, H2A histone family, member J; Scn1a, sodium channel, voltage-gated, type I, alpha. (b) Pathway analysis of genes responsive to restricted feeding in leptin receptor-deficient mice. Abbreviations: Il6st, interleukin 6 signal transducer; Ccnd2, cyclin D2; Prkaa2, protein kinase, AMP activated, alpha 2 catalytic subunit; Abcd2, ATP-binding cassette, subfamily D member 2; Pmch, pro-melanin-concentrating hormone; Gad1, glutamic acid decarboxylase 1, Rac1, RAS-related C3 botulinum substrate 1; Wnt7a, wingless-related MMTV integration site 7A; Myo5a, myosin VA; Gal, galanin; Grin1, glutamate receptor, ionotropic, NMDA1; Slc6a4, solute carrier family 6 member 4; Cdkn1a, cyclin-dependent kinase inhibitor 1A; Fgfr3, fibroblast growth factor receptor 3; En2, engrailed 2; Gbx2, gastrulation brain homeobox 2. (c) PCR validation of differences in gene expression after CR in *db/db* mice.

Quantitatively, the functional effects of CR implementation on hypothalamic gene transcription were more conserved across both metabolic context, that is, WT to *db/db*, than were the effects of running. *db/db* mice exhibited statistically significant changes in 488 genes following CR, and the majority (*n* = 287 transcripts) were significantly down-regulated (Figure 7(a), Table S6). Among the down-regulated genes were factors linked to transmembrane receptor (kinesin family member 3A, Kif3a [54]) and neuronal ion channel activity (voltage-gated, type I, alpha subunit, Scn1a [55]; glutamic acid decarboxylase 1, Gad1). Genes up-regulated following CR in *db/db* mice included transcripts for receptors and ligands associated with chemosensation and neuronal control of energy regulation (formyl peptide receptor 2, Fpr2 [56]: oxytocin, Oxt; pro-melanin-concentrating hormone, Pmch [57]). The expressions of Pmch, Oxt, Scn1a, and Gad1 were further evaluated using RT-PCR (Figures 7(b), 7(c)). Pmch and Oxt were increased, and GAD1 and Scn1a were decreased in these assays, in accordance with the microarray data. 

Significantly populated signaling pathways recruited following CR in *db/db* mice included “nervous system development,” “neurological disease,” and “cell-to-cell signaling” (Figure 7(b)). Gad1, wingless-related integration site 7A (Wnt7a), and cyclin-dependent kinase inhibitor 1A (Cdkn1) were represented in the “nervous system development” functional network. The significantly-populated signaling pathway “neurological disease” comprised both protein kinase, AMP-activated, alpha 2 catalytic subunit (Prkaa2) and ATP-binding cassette, subfamily D, member 2 (Abcd2).

### 3.8. Common and Distinct Functional Pathways Alterations in Response to Running and Caloric Restriction in C57Bl/6 and *db/db* Mice

**Figure 8:**
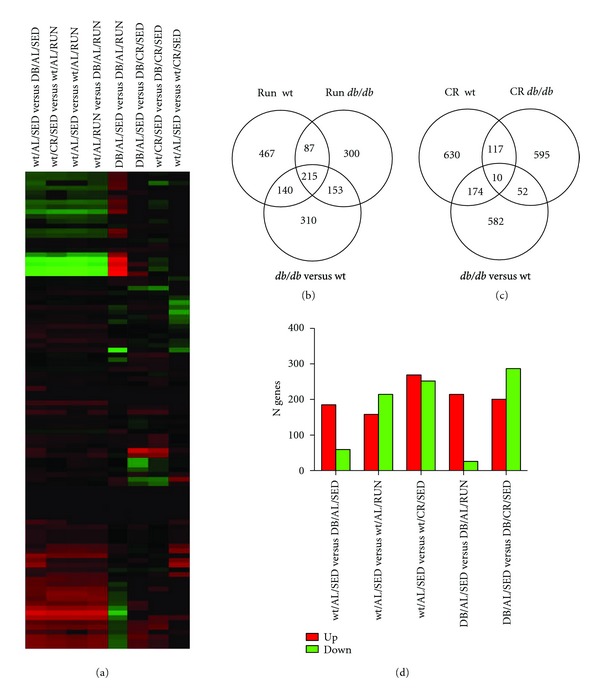
*db/db *and C57Bl/6 mice respond differently to exercise and caloric restriction. (a) Heat map profiles showing that *db/db *and C57Bl/6 mice exhibit distinct transcriptional responses to energetic challenges. (b) Venn diagram showing the number of genes expressed following voluntary wheel running in C57Bl/6 and *db/db *mice. (c) Venn diagram showing the number of transcripts responsive to CR in C57Bl/6 and *db/db *mice. (d) Side-by-side comparison of the number of genes that differ between C57Bl/6 and *db/db *mice following running or CR.

 Under sedentary conditions with *ad libitum* feeding, *db/db* mice differed from C57Bl/6 mice in a manner that was primarily attributable to transcriptional upregulation (Figure 8(a)). Numerically, there was a greater overlap between genotypes when comparing gene transcription following running (Figure 8(b)), relative to comparison across genotypes following CR (Figure 8(c)). Wheel running was associated with greater numbers of genes that were upregulated in *db/db* mice, while, in contrast, CR was predominantly associated with higher numbers of genes that were down-regulated in *db/db* mice (Figure 8(d)). 31 distinct, significantly populated, pathways were represented across the entire dataset. Among these pathways, only two categories contained genes that were significantly regulated across all experimental manipulations. These two categories were “nervous system development” and “tissue morphology”, suggesting that they are highly conserved functions.

**Figure 9:**
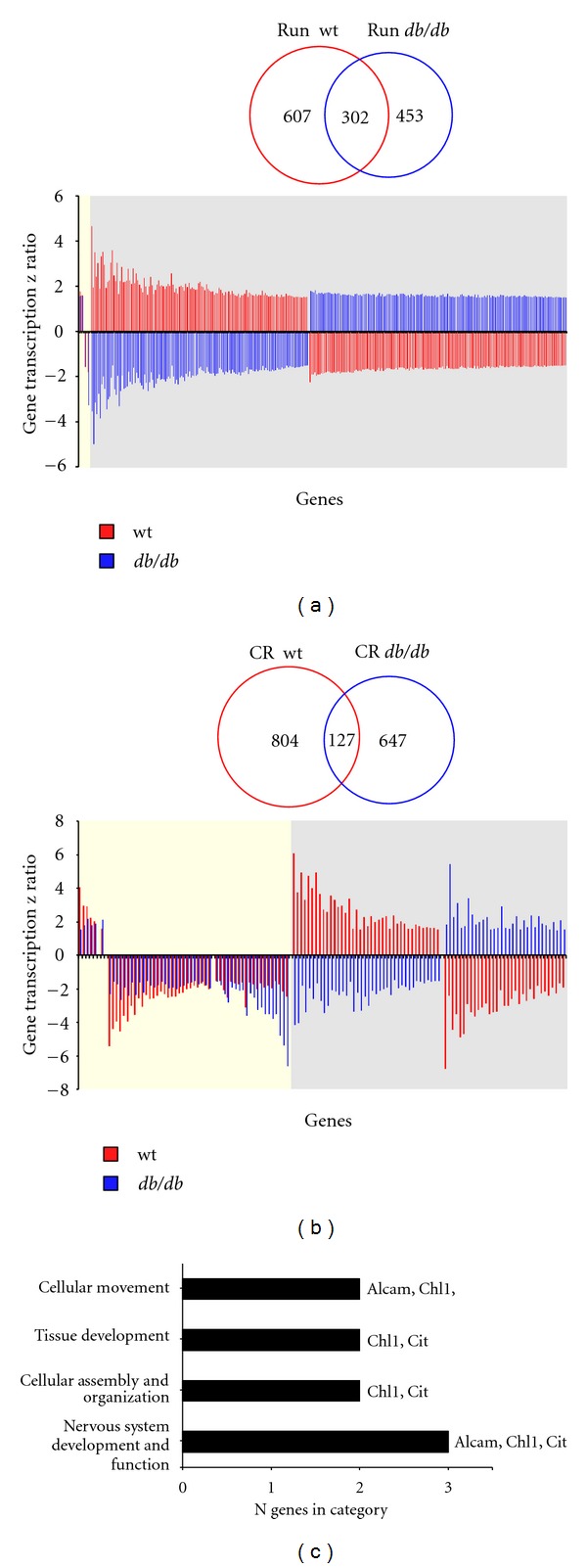
Physiological background differentially modulates the effects of exercise and caloric restriction upon hypothalamic transcriptomes. (a) Venn diagram illustrates the number of significantly regulated genes (up- and downregulated) affected by exercise (Run) in WT (red) or *db/db* (blue) animals. The associated histogram indicates the significantly regulated genes common to the effects of exercise in both genetic backgrounds (WT and *db/db*). The yellow area indicates the genes whose direction of regulation was conserved in both genetic backgrounds while the grey area shows the genes that showed reversal of their exercise-mediated expression profile. (b) Venn diagram and associated histogram depict similar data to (a) but for the implementation of CR to the WT (blue) or *db/db* (red) mice. (c) Significantly regulated pathways associated with the conserved CR-induced transcriptional activity in both WT and *db/db* mice.

 To assess directly the ability of the two imposed antiaging paradigms (CR and running) to modulate hypothalamic transcription in the diverse genomic backgrounds (WT, *db/db*), we created Venn diagrams of the specific transcriptomic effects. We found that between the WT and *db/db* backgrounds, running appeared to regulate more common genes (302: Figure 9(a), Table S7) compared to CR (127: Figure 9(b), Table S8). However, despite the greater numerical conservancy of running effects (compared to CR effects) between WT and *db/db*, only 1.6% of these genes were regulated in the same direction between both mouse backgrounds (Figure 9(a) histogram). In contrast, for the CR paradigm imposition upon the WT and *db/db* mice, a considerably greater percentage of the commonly regulated genes (42.5%) possessed the same polarity of expression across the two mouse genotypes. Taking this coregulated subset of genes [54] we investigated the potential biological functions of this conserved CR-mediated transcriptomic group (Figure 9(c)). We found that these CR effects upon hypothalamic transcription that were conserved in both WT and *db/db* mice were strongly associated with neuronal development (“nervous system development and function,” “tissue development”) and cellular architecture (“cellular movement,” “cellular assembly and organization”) (Figure 9(c)). Therefore, it appears that the actions of the antiaging CR paradigm, related to neuronal remodelling/rewiring in the hypothalamus, can be effected in animals of differing metabolic backgrounds. In contrast, the effects of running seem to be transcriptionally sensitive to the animals' metabolic background.

## 4. Discussion

An absence of leptin receptor signaling in the diabetic *db-db* mice significantly altered the expression of genes associated with excitatory synaptic transmission (Slc17a6 [58]) and neuronal development (Pias2 [59]) under *ad libitum*, sedentary conditions. Our data demonstrate that challenges to metabolic homeostasis regulated distinct hypothalamic gene-sets in wild-type and *db/db* mice. Although different genes were recruited following energetic challenges in wild-type and *db/db* mice, interestingly, transcriptional alterations following running or CR converged on functional pathways associated with nervous system development, suggesting that developmental signaling pathways are important for both neural circuit formation and plasticity in the adult hypothalamus.

Caloric restriction has previously been shown to alter neuronal morphology in the hypothalamus [60] and promotes synaptogenesis in select neuronal populations [23]. In our study, a significant number of genes that play important roles in regulating synaptic plasticity and cytoskeletal motility were differentially expressed in response to energetic challenges (e.g., Fkbp5 [32]; Chrd [40]; Hcrt [36]; App [61]). While our current study is primarily focused on investigating global alterations in hypothalamic gene transcription, the fact that we observed numerous significant alterations in the expression of multiple genes involved in regulating plasticity suggests the possibility that challenges to homeostasis can also reorganize circuitry across multiple other brain areas. The hypothalamic effects are likely to develop following an extended period of adaptation to caloric restriction in particular, as few changes in hypothalamic gene expression have been reported in response to shorter durations of CR [62]. As no other studies to date have profiled hypothalamic gene expression in the context of running, it is difficult to speculate on the time course for transcriptional adaptation and further studies are needed to investigate different time courses. Our data suggest that energetic challenges regulate gene transcription across the hypothalamus as a whole, and that the transcriptomic responses are dependent upon the genetic background (i.e., diabetic or nondiabetic). We are planning future studies that will focus on characterizing the genomic responses to energetic challenges that occur in individual hypothalamic nuclei, as this would shed further light upon how distinct neuronal populations respond to energetic challenges. Additionally, we also plan to extend our analyses to investigate overall hypothalamic proteome changes in response to energetic challenges. Due to the highly conserved nature of many neuroendocrine signaling pathways represented in our data, such as insulin/IGF-1, glucocorticoid, and androgen signaling, it is possible that the snapshot of transcriptional alterations captured in these experiments may include mechanisms for the establishment and maintenance of a metabolic set point in other species, with relevance for the prevention and treatment of obesity. Additionally, gaining a greater understanding of how different genetic backgrounds (e.g., diabetic or nondiabetic) can alter hypothalamic transcriptomic responses to energetic challenges, could pave the way for the development of novel therapeutics for the treatment of hypothalamic dysfunction. Our data further suggests that therapeutic caloric restriction paradigms, or pharmacomimetics thereof, may be more readily applicable (compared to exercise paradigms) across different patients with diverse metabolic backgrounds.

## Supplementary Material

Table S1 demonstrates the hypothalamic validation PCR primer sequences employed in this study. Hypothalamic gene transcripts differentially expressed in *db/db *compared to C57Bl/6 (wt) mice, both under *ad libitum* sedentary conditions are outlined in Table S2. Hypothalamic gene transcripts differentially expressed in running- *ad libitum* C57Bl/6 mice compared to sedentary- *ad libitum* C57Bl/6 mice are outlined in Table S3. Hypothalamic gene transcripts differentially expressed in sedentary-caloric restriction (CR) C57Bl/6 mice compared to sedentary- *ad libitum* (AL) C57Bl/6 mice are outlined in Table S4. Hypothalamic gene transcripts differentially expressed in running (run) -*ad libitumdb/db* mice compared to sedentary (sed) -*ad libitum db/db* mice are outlined in Table S5. Hypothalamic gene transcripts differentially expressed in sedentary-caloric restriction (CR) *db/db* mice compared to sedentary- *ad libitum* (AL) *db/db* mice are outlined in Table S6. Hypothalamic gene transcripts commonly regulated by running in WT and *db/db ad libitum* fed mice are outlined in Table S7. Hypothalamic gene transcripts commonly regulated by CR in WT and *db/db *mice are outlined in Table S8.

## References

[1] Wang L, Chadwick W, Park SS (2010). Gonadotropin-releasing hormone receptor system: modulatory role in aging and neurodegeneration. *CNS and Neurological Disorders*.

[2] Martin B, Maudsley S, McNeilly J (2009). Neonatal estrogenic effects upon the male rat pituitary: early gonadotrophin attenuation precedes long-term recovery. *NeuroMolecular Medicine*.

[3] Martin B, Golden E, Carlson OD, Egan JM, Mattson MP, Maudsley S (2008). Caloric restriction: impact upon pituitary function and reproduction. *Ageing Research Reviews*.

[4] Jackson K, Vieira Silva HM, Zhang W, Michelini LC, Stern JE (2005). Exercise training differentially affects intrinsic excitability of autonomic and neuroendocrine neurons in the hypothalamic paraventricular nucleus. *Journal of Neurophysiology*.

[5] Sternson SM, Shepherd GMG, Friedman JM (2005). Topographic mapping of VMH arcuate nucleus microcircuits and their reorganization by fasting. *Nature Neuroscience*.

[6] Pinto S, Roseberry AG, Liu H (2004). Rapid rewiring of arcuate nucleus feeding circuits by leptin. *Science*.

[7] Maudsley S, Martin B, Egan JM (2011). To be or not to—obese. *Endocrinology*.

[8] Martin B, Ji S, Maudsley S, Mattson MP (2010). ‘Control’ laboratory rodents are metabolically morbid: why it matters. *Proceedings of the National Academy of Sciences of the United States of America*.

[9] Stranahan AM, Zhou Y, Martin B, Maudsley S (2009). Pharmacomimetics of exercise: novel approaches for hippocampally-targeted neuroprotective agents. *Current Medicinal Chemistry*.

[10] Martin B, Golden E, Egan JM (2007). Reduced energy intake: the secret to a long and healthy life?. *IBS Journal of Science*.

[11] Martin B, Mattson MP, Maudsley S (2006). Caloric restriction and intermittent fasting: two potential diets for successful brain aging. *Ageing Research Reviews*.

[12] Hummel KP, Dickie MM, Coleman DL (1966). Diabetes, a new mutation in the mouse. *Science*.

[13] Bouret SG, Draper SJ, Simerly RB (2004). Trophic action of leptin on hypothalamic neurons that regulate feeding. *Science*.

[14] Bouret SG, Gorski JN, Patterson CM, Chen S, Levin BE, Simerly RB (2008). Hypothalamic neural projections are permanently disrupted in diet-induced obese rats. *Cell Metabolism*.

[15] Martin B, Pearson M, Kebejian L (2007). Sex-dependent metabolic, neuroendocrine, and cognitive responses to dietary energy restriction and excess. *Endocrinology*.

[16] Martin B, Pearson M, Brenneman R (2008). Conserved and differential effects of dietary energy intake on the hippocampal transcriptomes of females and males. *PLoS ONE*.

[17] Martin B, Pearson M, Brenneman R (2009). Gonadal transcriptome alterations in response to dietary energy intake: sensing the reproductive environment. *PLoS ONE*.

[18] Tung YCL, Ma M, Piper S, Coll A, O’Rahilly S, Yeo GSH (2008). Novel leptin-regulated genes revealed by transcriptional profiling of the hypothalamic paraventricular nucleus. *Journal of Neuroscience*.

[19] Draper S, Kirigiti M, Glavas M (2010). Differential gene expression between neuropeptide y expressing neurons of the dorsomedial nucleus of the Hypothalamus and the Arcuate Nucleus: microarray analysis study. *Brain Research*.

[20] Wu P, Jiang C, Shen Q, Hu Y (2009). Systematic gene expression profile of hypothalamus in calorie-restricted mice implicates the involvement of mTOR signaling in neuroprotective activity. *Mechanisms of Ageing and Development*.

[21] Kim Y, Park M, Boghossian S, York DA (2010). Three weeks voluntary running wheel exercise increases endoplasmic reticulum stress in the brain of mice. *Brain Research*.

[22] Bi S, Scott KA, Hyun J, Ladenheim EE, Moran TH (2005). Running wheel activity prevents hyperphagia and obesity in Otsuka Long-Evans Tokushima fatty rats: role of hypothalamic signaling. *Endocrinology*.

[23] Horvath TL (2005). The hardship of obesity: a soft-wired hypothalamus. *Nature Neuroscience*.

[24] Stranahan AM, Lee K, Martin B (2009). Voluntary exercise and caloric restriction enhance hippocampal dendritic spine density and BDNF levels in diabetic mice. *Hippocampus*.

[25] Stranahan AM, Lee K, Becker KG (2010). Hippocampal gene expression patterns underlying the enhancement of memory by running in aged mice. *Neurobiology of Aging*.

[26] Park SS, Stranahan AM, Chadwick W (2011). Cortical gene transcription response patterns to water maze training in aged mice. *BMC Neuroscience*.

[27] Lein ES, Hawrylycz MJ, Ao N (2007). Genome-wide atlas of gene expression in the adult mouse brain. *Nature*.

[28] Paxinos G, Franklin KBJ (2001). *The Mouse Brain in Stereotaxic Coordinates*.

[29] Morash B, Li A, Murphy PR, Wilkinson M, Ur E (1999). Leptin gene expression in the brain and pituitary gland. *Endocrinology*.

[30] Brown RE (2008). Could there be a fine-tuning role for brain-derived adipokines in the regulation of bodyweight and prevention of obesity?. *McGill Journal of Medicine*.

[31] Campbell JE, Király MA, Atkinson DJ, D’Souza AM, Vranic M, Riddell MC (2010). Regular exercise prevents the development of hyperglucocorticoidemia via adaptations in the brain and adrenal glands in male Zucker diabetic fatty rats. *The American Journal of Physiology*.

[32] Quintá HR, Maschi D, Gomez-Sanchez C, Piwien-Pilipuk G, Galigniana MD (2010). Subcellular rearrangement of hsp90-binding immunophilins accompanies neuronal differentiation and neurite outgrowth. *Journal of Neurochemistry*.

[33] So CW, Sham MH, Chew SL (2000). Expression and protein-binding studies of the EEN gene family, new interacting partners for dynamin, synaptojanin and huntingtin proteins. *Biochemical Journal*.

[34] Domanitskaya E, Wacker A, Mauti O (2010). Sonic hedgehog guides post-crossing commissural axons both directly and indirectly by regulating Wnt activity. *Journal of Neuroscience*.

[35] Cai Y, Jin J, Florens L (2005). The mammalian YL1 protein is a shared subunit of the TRRAP/TIP60 histone acetyltransferase and SRCAP complexes. *Journal of Biological Chemistry*.

[36] Ganjavi H, Shapiro CM (2007). Hypocretin/orexin: a molecular link between sleep, energy regulation, and pleasure. *Journal of Neuropsychiatry and Clinical Neurosciences*.

[37] Zhou Y, Rui L (2010). Major urinary protein regulation of chemical communication and nutrient metabolism. *Vitamins and Hormones*.

[38] Jovanovic Z, Yeo GSH (2010). Central leptin signalling: beyond the arcuate nucleus. *Autonomic Neuroscience*.

[39] Lee CS, Yi JS, Jung SY (2010). TRIM72 negatively regulates myogenesis via targeting insulin receptor substrate-1. *Cell Death and Differentiation*.

[40] Sun M, Thomas MJ, Herder R, Bofenkamp ML, Selleck SB, O’Connor MB (2007). Presynaptic contributions of chordin to hippocampal plasticity and spatial learning. *Journal of Neuroscience*.

[41] Johnson CP, Chapman ER (2010). Otoferlin is a calcium sensor that directly regulates SNARE-mediated membrane fusion. *Journal of Cell Biology*.

[42] Ramesh N, Geha R (2009). Recent advances in the biology of WASP and WIP. *Immunologic Research*.

[43] Mercader JM, Saus E, Agüera Z (2008). Association of NTRK3 and its interaction with NGF suggest an altered cross-regulation of the neurotrophin signaling pathway in eating disorders. *Human Molecular Genetics*.

[44] Simonis-Bik AM, Nijpels G, van Haeften TW (2010). Gene variants in the novel type 2 diabetes loci CDC123/CAMK1D, THADA, ADAMTS9, BCL11A, and MTNR1B affect different aspects of pancreatic *β*-cell function. *Diabetes*.

[45] Armstrong L, Lako M, van Herpe I, Evans J, Saretzki G, Hole N (2004). A role for nucleoprotein Zap3 in the reduction of telomerase activity during embryonic stem cell differentiation. *Mechanisms of Development*.

[46] Wolfe DE, Schindler D, Desnick RJ (1995). Neuroaxonal dystrophy in infantile *α*-N-acetylgalactosaminidase deficiency. *Journal of the Neurological Sciences*.

[47] Zhang L, Anglesio MS, O’Sullivan M (2007). The E3 ligase HACE1 is a critical chromosome 6q21 tumor suppressor involved in multiple cancers. *Nature Medicine*.

[48] Cannella B, Hoban CJ, Gao YL (1998). The neuregulin, glial growth factor 2, diminishes autoimmune demyelination and enhances remyelination in a chronic relapsing model for multiple sclerosis. *Proceedings of the National Academy of Sciences of the United States of America*.

[49] Park MH (2006). The post-translational synthesis of a polyamine-derived amino acid, hypusine, in the eukaryotic translation initiation factor 5A (eIF5A). *Journal of Biochemistry*.

[50] Rees MI, Lewis TM, Vafa B (2001). Compound heterozygosity and nonsense mutations in the *α*1-subunit of the inhibitory glycine receptor in hyperekplexia. *Human Genetics*.

[51] Méndez-Gómez HR, Vergaño-Vera E, Abad JL (2011). The T-box brain 1 (Tbr1) transcription factor inhibits astrocyte formation in the olfactory bulb and regulates neural stem cell fate. *Molecular and Cellular Neuroscience*.

[52] Haft CR, de la Luz Sierra M, Barr VA, Haft DH, Taylor SI (1998). Identification of a family of sorting nexin molecules and characterization of their association with receptors. *Molecular and Cellular Biology*.

[53] Spencer N, Hopkinson DA (1980). Biochemical genetics of the pentose phosphate cycle: human ribose 5-phosphate isomerase (RPI) and ribulose 5-phosphate 3-epimerase (RPE). *Annals of Human Genetics*.

[54] Kovacs JJ, Whalen EJ, Liu R (2008). *β*-arrestin-mediated localization of smoothened to the primary cilium. *Science*.

[55] Goldin AL, Snutch T, Lubbert H (1986). Messenger RNA coding for only the *α* subunit of the rat brain Na channel is sufficient for expression of functional channels in Xenopus oocytes. *Proceedings of the National Academy of Sciences of the United States of America*.

[56] Le Y, Yazawa H, Gong W (2001). Cutting edge: the neurotoxic prion peptide fragment PrP106-126 is a chemotactic agonist for the G protein-coupled receptor formyl peptide receptor-like 11,2. *Journal of Immunology*.

[57] Qu D, Ludwig DS, Gammeltoft S (1996). A role for melanin-concentrating hormone in the central regulation of feeding behaviour. *Nature*.

[58] Korosi A, Shanabrough M, McClelland S (2010). Early-life experience reduces excitation to stress-responsive hypothalamic neurons and reprograms the expression of corticotropin-releasing hormone. *Journal of Neuroscience*.

[59] Adhikary S, Peukert K, Karsunky H (2003). Miz1 is required for early embryonic development during gastrulation. *Molecular and Cellular Biology*.

[60] Flanagan-Cato LM, Fluharty SJ, Weinreb EB, Labelle DR (2008). Food restriction alters neuronal morphology in the hypothalamic ventromedial nucleus of male rats. *Endocrinology*.

[61] Lee KJ, Moussa CEH, Lee Y (2010). Beta amyloid-independent role of amyloid precursor protein in generation and maintenance of dendritic spines. *Neuroscience*.

[62] Yamamoto Y, Tanahashi T, Kawai T (2009). Changes in behavior and gene expression induced by caloric restriction in C57BL/6 mice. *Physiological Genomics*.

