# Supplementary material for: Metabolic Context Regulates Distinct Hypothalamic Transcriptional Responses to Antiaging Interventions
Source: Int J Endocrinol. 2012 Aug 27;2012:732975. doi: 10.1155/2012/732975 (PMC3427989; doi:10.1155/2012/732975)
Supplement: Supplementary file 1 — Table S1 demonstrates the hypothalamic validation PCR primer sequences employed in this study. Hypothalamic gene transcripts differentially expressed in db/db compared to C57Bl/6 (wt) mice, both under ad libitum sedentary conditions are outlined in Table S2. Hypothalamic gene transcripts differentially expressed in running- ad libitum C57Bl/6 mice compared to sedentary- ad libitum C57Bl/6 mice are outlined in Table S3. Hypothalamic gene transcripts differentially expressed in sedentary-caloric restriction (CR) C57Bl/6 mice compared to sedentary- ad libitum (AL) C57Bl/6 mice are outlined in Table S4. Hypothalamic gene transcripts differentially expressed in running (run) -ad libitumdb/db mice compared to sedentary (sed) -ad libitum db/db mice are outlined in Table S5. Hypothalamic gene transcripts differentially expressed in sedentary-caloric restriction (CR) db/db mice compared to sedentary- ad libitum (AL) db/db mice are outlined in Table S6. Hypothalamic gene transcripts commonly regulated by running in WT and db/db ad libitum fed mice are outlined in Table S7. Hypothalamic gene transcripts commonly regulated by CR in WT and db/db mice are outlined in Table S8. [file 732975.f1.doc]

**Table S1.** Hypothalamic validation PCR primer sequences employed.

| **Gene Target** | **Forward primer** | **Reverrse primer** | **Product size** |
| --- | --- | --- | --- |
| Gpx7 | acaccaacagggagattgag | ggagtgaagggaagagaagc | 367 |
| Tomm22 | ttcttcccgttgtctttgag | agcattggagtgagaacagg | 364 |
| Sfrp1 | acaacgtgggctacaagaag | gttcaatgatggcctctgac | 396 |
| Lep | tgtccaagatggaccagactc | actggtctgaggcagggagca | 155 |
| Fkbp5 | atgcatcaagccaaagtctc | gctgcaaggctagtgaagag | 499 |
| Igf1 | catcatgtcgtcttcacacc | ctgagtcttgggcatgtcag | 325 |
| Ntrk3 | agcctttactgcatcagtgc | ccacacgtggagggtagtag | 367 |
| Camk1d | acagagaaagatgccagcac | tctgccttgaggatctgttc | 362 |
| Tbr1 | tgactccaaggactcaccag | acaggtatacttgcgccttg | 419 |
| Nrg11 | tagcccattgaggatagtgg | ttcttcctgtgtggagaagc | 328 |
| Naga | actcctggaagagtgtgctg | cacttcgatatgggatttgc | 320 |
| Gad1 | agatgtgtgcaggctacctc | agatcctgacccaacctctc | 496 |
| Pmch | ctgcagaaagatccgttgtc | ccaacatggtcggtagactc | 332 |
| Oxt | tgtgctggacctggatatg | gaagcgcgctaaaggtattc | 351 |
| Scn1a | gccacaaccaaacaaacttc | tgtggacattgtcaggtcag | 415 |

**Table S2.** Hypothalamic gene transcripts differentially expressed in *db/db* compared to C57Bl/6 (WT) mice, both under ad libitum sedentary conditions (n=3 animals per group). Statistical significance was considered at p≤0.05.

| **Gene Symbol** | **Description** | **z-ratio (db/db vs. WT)** |
| --- | --- | --- |
| Eif3j | eukaryotic translation initiation factor 3, subunit J | 5.609117706 |
| Ccdc94 | coiled-coil domain containing 94 | 5.317015389 |
| Dhx9 | DEAH (Asp-Glu-Ala-His) box polypeptide 9 | 4.585493845 |
| Gimap1 | GTPase, IMAP family member 1 | 4.497791002 |
| Csda | cold shock domain protein A | 3.942549061 |
| Pofut2 | protein O-fucosyltransferase 2 | 3.910884109 |
| Myl4 | myosin, light chain 4, alkali; atrial, embryonic | 3.67530989 |
| Chst12 | carbohydrate (chondroitin 4) sulfotransferase 12 | 3.657874345 |
| Grhl1 | grainyhead-like 1 (Drosophila) | 3.576344756 |
| Prrc1 | proline-rich coiled-coil 1 | 3.55104373 |
| Gpr180 | G protein-coupled receptor 180 | 2.989492972 |
| Serf1a | small EDRK-rich factor 1A (telomeric) | 2.952752273 |
| Rage | renal tumor antigen | 2.932303201 |
| E2f4 | E2F transcription factor 4, p107/p130-binding | 2.866138146 |
| Brdt | bromodomain, testis-specific | 2.815645072 |
| Slc45a4 | solute carrier family 45, member 4 | 2.794313905 |
| Necab3 | N-terminal EF-hand calcium binding protein 3 | 2.793768753 |
| Fzd5 | frizzled homolog 5 (Drosophila) | 2.753107699 |
| Lpl | lipoprotein lipase | 2.750493867 |
| Spns1 | spinster homolog 1 (Drosophila) | 2.7114154 |
| Fbxo36 | F-box protein 36 | 2.694608003 |
| Gpx7 | glutathione peroxidase 7 | 2.689539262 |
| Gfpt2 | glutamine-fructose-6-phosphate transaminase 2 | 2.677889967 |
| Cryba4 | crystallin, beta A4 | 2.592378838 |
| Igdcc3 | immunoglobulin superfamily, DCC subclass, member 3 | 2.549095808 |
| Dnm1 | dynamin 1 | 2.547837546 |
| P4ha1 | prolyl 4-hydroxylase, alpha polypeptide I | 2.53872414 |
| Usp8 | ubiquitin specific peptidase 8 | 2.537219155 |
| Cpxm1 | carboxypeptidase X (M14 family), member 1 | 2.49386401 |
| Usp28 | ubiquitin specific peptidase 28 | 2.478097726 |
| Cd52 | CD52 molecule | 2.472827407 |
| Phf14 | PHD finger protein 14 | 2.425242378 |
| Doc2a | double C2-like domains, alpha | 2.412195689 |
| Luc7l | LUC7-like (S. cerevisiae) | 2.40012365 |
| Cideb | cell death-inducing DFFA-like effector b | 2.382723852 |
| Xrcc2 | X-ray repair complementing defective repair in Chinese hamster cells 2 | 2.375913332 |
| Zc3h6 | zinc finger CCCH-type containing 6 | 2.364668489 |
| Rhoj | ras homolog gene family, member J | 2.342315889 |
| Col15a1 | collagen, type XV, alpha 1 | 2.337272743 |
| Pnpla7 | patatin-like phospholipase domain containing 7 | 2.302522366 |
| Chic1 | cysteine-rich hydrophobic domain 1 | 2.301348181 |
| Ccdc51 | coiled-coil domain containing 51 | 2.279979429 |
| EVc | Ellis van Creveld syndrome | 2.279537219 |
| Mmp2 | matrix metallopeptidase 2 (gelatinase A, 72kDa gelatinase, 72kDa type IV collagenase) | 2.277297679 |
| Hepacam | hepatocyte cell adhesion molecule | 2.272914313 |
| C14orf68 | chromosome 14 open reading frame 68 | 2.26664118 |
| E2f5 | E2F transcription factor 5, p130-binding | 2.234674978 |
| Dzip1 | DAZ interacting protein 1 | 2.231015968 |
| Slc16a9 | solute carrier family 16, member 9 (monocarboxylic acid transporter 9) | 2.228241262 |
| Ndufb2 | NADH dehydrogenase (ubiquinone) 1 beta subcomplex, 2, 8kDa | 2.209412977 |
| Cep63 | centrosomal protein 63kDa | 2.191659786 |
| Haus1 | HAUS augmin-like complex, subunit 1 | 2.190796246 |
| Foxo1 | forkhead box O1 | 2.186905862 |
| P4ha2 | prolyl 4-hydroxylase, alpha polypeptide II | 2.175464363 |
| Gfm1 | G elongation factor, mitochondrial 1 | 2.170710878 |
| AVpi1 | arginine vasopressin-induced 1 | 2.169135836 |
| Unc119 | unc-119 homolog (C. elegans) | 2.152179774 |
| Higd1b | HIG1 hypoxia inducible domain family, member 1B | 2.142062889 |
| Gpr153 | G protein-coupled receptor 153 | 2.119191672 |
| Spc24 | SPC24, NDC80 kinetochore complex component, homolog (S. cerevisiae) | 2.118889394 |
| Psd3 | pleckstrin and Sec7 domain containing 3 | 2.098096351 |
| Ddt | D-dopachrome tautomerase | 2.084004688 |
| Fzd7 | frizzled homolog 7 (Drosophila) | 2.07896409 |
| Mospd3 | motile sperm domain containing 3 | 2.06704062 |
| Atcay | ataxia, cerebellar, Cayman type | 2.0621852 |
| Phf12 | PHD finger protein 12 | 2.051602328 |
| Pik3ca | phosphoinositide-3-kinase, catalytic, alpha polypeptide | 2.048924708 |
| Ankrd35 | ankyrin repeat domain 35 | 2.038827365 |
| Cap1 | CAP, adenylate cyclase-associated protein 1 (yeast) | 2.018784142 |
| Hist1h4j | histone cluster 1, H4j | 2.002361386 |
| Cox6a2 | cytochrome c oxidase subunit VIa polypeptide 2 | 1.994990804 |
| Agpat3 | 1-acylglycerol-3-phosphate O-acyltransferase 3 | 1.986949454 |
| Gng8 | guanine nucleotide binding protein (G protein), gamma 8 | 1.974853993 |
| Cobll1 | COBL-like 1 | 1.970696789 |
| Il13ra1 | interleukin 13 receptor, alpha 1 | 1.969564369 |
| Dnajc17 | DnaJ (Hsp40) homolog, subfamily C, member 17 | 1.965797686 |
| Angptl4 | angiopoietin-like 4 | 1.939006043 |
| Cnga4 | cyclic nucleotide gated channel alpha 4 | 1.919387851 |
| Mt2a | metallothionein 2A | 1.913665986 |
| Eml5 | echinoderm microtubule associated protein like 5 | 1.896798665 |
| Ecel1 | endothelin converting enzyme-like 1 | 1.886560737 |
| Atp6V0a1 | ATPase, H+ transporting, lysosomal V0 subunit a1 | 1.882476621 |
| Mad1l1 | MAD1 mitotic arrest deficient-like 1 (yeast) | 1.88027458 |
| Vars2 | valyl-tRNA synthetase 2, mitochondrial (putative) | 1.8587447 |
| Spata2l | spermatogenesis associated 2-like | 1.858191155 |
| Mrps2 | mitochondrial ribosomal protein S2 | 1.851715147 |
| Cdh6 | cadherin 6, type 2, K-cadherin (fetal kidney) | 1.83685185 |
| Nr1h3 | nuclear receptor subfamily 1, group H, member 3 | 1.834064427 |
| Map1lc3a | microtubule-associated protein 1 light chain 3 alpha | 1.832002299 |
| Abcb6 | ATP-binding cassette, sub-family B (MDR/TAP), member 6 | 1.831213375 |
| Spag6 | sperm associated antigen 6 | 1.826636965 |
| Tcf12 | transcription factor 12 | 1.820788765 |
| Fzd2 | frizzled homolog 2 (Drosophila) | 1.814036989 |
| Tm9sf2 | transmembrane 9 superfamily member 2 | 1.810567201 |
| Ankrd10 | ankyrin repeat domain 10 | 1.809337326 |
| Glo1 | glyoxalase I | 1.808568633 |
| Myef2 | myelin expression factor 2 | 1.806382634 |
| Mapk4 | mitogen-activated protein kinase 4 | 1.794433362 |
| Heatr3 | HEAT repeat containing 3 | 1.786154131 |
| Hlcs | holocarboxylase synthetase (biotin-(proprionyl-Coenzyme A-carboxylase (ATP-hydrolysing)) ligase) | 1.782534903 |
| Ephx2 | epoxide hydrolase 2, cytoplasmic | 1.756692373 |
| Nudt1 | nudix (nucleoside diphosphate linked moiety X)-type motif 1 | 1.754879327 |
| Ccne1 | cyclin E1 | 1.748221453 |
| Cyp46a1 | cytochrome P450, family 46, subfamily A, polypeptide 1 | 1.747353423 |
| Clec11a | C-type lectin domain family 11, member A | 1.743467714 |
| Senp7 | SUMO1/sentrin specific peptidase 7 | 1.738627736 |
| Scly | selenocysteine lyase | 1.736816878 |
| Slc17a6 | solute carrier family 17 (sodium-dependent inorganic phosphate cotransporter), member 6 | 1.735807856 |
| Dapk2 | death-associated protein kinase 2 | 1.733684875 |
| Gabrd | gamma-aminobutyric acid (GABA) A receptor, delta | 1.732209947 |
| Vwa5a | von Willebrand factor A domain containing 5A | 1.728441849 |
| Abcb4 | ATP-binding cassette, sub-family B (MDR/TAP), member 4 | 1.72409191 |
| Zc3h14 | zinc finger CCCH-type containing 14 | 1.722388262 |
| Ripk1 | receptor (TNFRSF)-interacting serine-threonine kinase 1 | 1.715677132 |
| Ndfip1 | Nedd4 family interacting protein 1 | 1.715606227 |
| Prkcd | protein kinase C, delta | 1.701160723 |
| Eif2ak2 | eukaryotic translation initiation factor 2-alpha kinase 2 | 1.699279616 |
| Lypla1 | lysophospholipase I | 1.698096921 |
| Cstf3 | cleavage stimulation factor, 3' pre-RNA, subunit 3, 77kDa | 1.691538006 |
| Ywhah | tyrosine 3-monooxygenase/tryptophan 5-monooxygenase activation protein, eta polypeptide | 1.691104867 |
| Tmem136 | transmembrane protein 136 | 1.687692163 |
| Slc25a37 | solute carrier family 25, member 37 | 1.675974084 |
| Hspa8 | heat shock 70kDa protein 8 | 1.673330593 |
| Rtn3 | reticulon 3 | 1.67098441 |
| Nphp1 | nephronophthisis 1 (juvenile) | 1.668308303 |
| Atpif1 | ATPase inhibitory factor 1 | 1.667034608 |
| Tuba1c | tubulin, alpha 1c | 1.666471276 |
| Plekha6 | pleckstrin homology domain containing, family A member 6 | 1.665671104 |
| Cox6b1 | cytochrome c oxidase subunit Vib polypeptide 1 (ubiquitous) | 1.662672072 |
| Sgpl1 | sphingosine-1-phosphate lyase 1 | 1.661589892 |
| Arhgap18 | Rho GTPase activating protein 18 | 1.653378247 |
| Cdkn1b | cyclin-dependent kinase inhibitor 1B (p27, Kip1) | 1.649294488 |
| Uqcrh | ubiquinol-cytochrome c reductase hinge protein | 1.644123798 |
| Camk2n1 | calcium/calmodulin-dependent protein kinase II inhibitor 1 | 1.640311454 |
| Pgbd5 | piggyBac transposable element derived 5 | 1.637750986 |
| Tnip1 | TNFAIP3 interacting protein 1 | 1.636822087 |
| Cep170 | centrosomal protein 170kDa | 1.631835278 |
| Limd1 | LIM domains containing 1 | 1.629215259 |
| Reck | reversion-inducing-cysteine-rich protein with kazal motifs | 1.626089249 |
| Gapdh | glyceraldehyde-3-phosphate dehydrogenase | 1.625436295 |
| Ecm1 | extracellular matrix protein 1 | 1.624694749 |
| Rsf1 | remodeling and spacing factor 1 | 1.623696876 |
| Ndor1 | NADPH dependent diflavin oxidoreductase 1 | 1.62001279 |
| Cyb5a | cytochrome b5 type A (microsomal) | 1.618857967 |
| Mpst | mercaptopyruvate sulfurtransferase | 1.613751704 |
| Tyrobp | TYRO protein tyrosine kinase binding protein | 1.608177304 |
| Wdr8 | WD repeat domain 8 | 1.606765783 |
| Grm7 | glutamate receptor, metabotropic 7 | 1.602780779 |
| Sgk1 | serum/glucocorticoid regulated kinase 1 | 1.602655161 |
| St7 | suppression of tumorigenicity 7 | 1.601298266 |
| Stk19 | serine/threonine kinase 19 | 1.58923018 |
| Renbp | renin binding protein | 1.588699877 |
| Sparc | secreted protein, acidic, cysteine-rich (osteonectin) | 1.572542923 |
| Sgta | small glutamine-rich tetratricopeptide repeat (TPR)-containing, alpha | 1.571302714 |
| Adck4 | aarF domain containing kinase 4 | 1.570698176 |
| Nsmce2 | non-SMC element 2, MMS21 homolog (S. cerevisiae) | 1.560442717 |
| C6orf130 | chromosome 6 open reading frame 130 | 1.559683488 |
| Pias2 | protein inhibitor of activated STAT, 2 | 1.555651171 |
| Kdelr2 | KDEL (Lys-Asp-Glu-Leu) endoplasmic reticulum protein retention receptor 2 | 1.555249321 |
| Srp54 | signal recognition particle 54kDa | 1.549254813 |
| Prkar1a | protein kinase, cAMP-dependent, regulatory, type I, alpha (tissue specific extinguisher 1) | 1.54889696 |
| Eif4h | eukaryotic translation initiation factor 4H | 1.547886145 |
| Rab2a | RAB2A, member RAS oncogene family | 1.546615566 |
| App | amyloid beta (A4) precursor protein | 1.545173946 |
| Trim59 | tripartite motif-containing 59 | 1.54358431 |
| Taf9 | TAF9 RNA polymerase II, TATA box binding protein (TBP)-associated factor, 32kDa | 1.542277995 |
| Mt3 | metallothionein 3 | 1.539981979 |
| Arid5b | AT rich interactive domain 5B (MRF1-like) | 1.539713428 |
| Dcx | doublecortin | 1.533975286 |
| Uba1 | ubiquitin-like modifier activating enzyme 1 | 1.533230412 |
| Atp2b3 | ATPase, Ca++ transporting, plasma membrane 3 | 1.531114535 |
| Tmem159 | transmembrane protein 159 | 1.52782865 |
| Inha | inhibin, alpha | 1.527560235 |
| Mrps15 | mitochondrial ribosomal protein S15 | 1.527552066 |
| Aplp2 | amyloid beta (A4) precursor-like protein 2 | 1.526224013 |
| Hic2 | hypermethylated in cancer 2 | 1.521521358 |
| Aldoc | aldolase C, fructose-bisphosphate | 1.520000903 |
| Pkm2 | pyruvate kinase, muscle | 1.519893032 |
| Tubb4 | tubulin, beta 4 | 1.51916847 |
| Sh3bgrl | SH3 domain binding glutamic acid-rich protein like | 1.514631386 |
| Wbp1 | WW domain binding protein 1 | 1.513604308 |
| Nisch | nischarin | 1.510538137 |
| Snx2 | sorting nexin 2 | 1.508129034 |
| Timm17b | translocase of inner mitochondrial membrane 17 homolog B (yeast) | 1.501856162 |
| Ypel3 | yippee-like 3 (Drosophila) | 1.501028243 |
| Efhc2 | EF-hand domain (C-terminal) containing 2 | -1.500178599 |
| Prss35 | protease, serine, 35 | -1.500306542 |
| Scnm1 | sodium channel modifier 1 | -1.500603588 |
| Ido1 | indoleamine 2,3-dioxygenase 1 | -1.503090227 |
| Cntln | centlein, centrosomal protein | -1.505219488 |
| H1f0 | H1 histone family, member 0 | -1.505605478 |
| Ccdc113 | coiled-coil domain containing 113 | -1.506055358 |
| Rprd1a | regulation of nuclear pre-mRNA domain containing 1A | -1.50705302 |
| Pcp2 | Purkinje cell protein 2 | -1.51007968 |
| Sepsecs | Sep (O-phosphoserine) tRNA:Sec (selenocysteine) tRNA synthase | -1.51064911 |
| Mosc2 | MOCO sulphurase C-terminal domain containing 2 | -1.512837595 |
| Klhl5 | kelch-like 5 (Drosophila) | -1.513579607 |
| Selp | selectin P (granule membrane protein 140kDa, antigen CD62) | -1.514164054 |
| Crym | crystallin, mu | -1.514449088 |
| Ing5 | inhibitor of growth family, member 5 | -1.514934907 |
| Pde6g | phosphodiesterase 6G, cGMP-specific, rod, gamma | -1.515704396 |
| Palm2 | paralemmin 2 | -1.515915452 |
| Sag | S-antigen; retina and pineal gland (arrestin) | -1.516037629 |
| F8 | coagulation factor VIII, procoagulant component | -1.517246943 |
| Ptpn1 | protein tyrosine phosphatase, non-receptor type 1 | -1.518804692 |
| Dppa5 | developmental pluripotency associated 5 | -1.520542962 |
| Figf | c-fos induced growth factor (vascular endothelial growth factor D) | -1.521844074 |
| Kal1 | Kallmann syndrome 1 sequence | -1.52271733 |
| Tasp1 | taspase, threonine aspartase, 1 | -1.525769707 |
| Sln | sarcolipin | -1.52807447 |
| Morn2 | MORN repeat containing 2 | -1.528297181 |
| Tnfrsf25 | tumor necrosis factor receptor superfamily, member 25 | -1.531390924 |
| Ly96 | lymphocyte antigen 96 | -1.533002589 |
| Elf2 | E74-like factor 2 (ets domain transcription factor) | -1.533473471 |
| Echdc1 | enoyl Coenzyme A hydratase domain containing 1 | -1.536008278 |
| Usp10 | ubiquitin specific peptidase 10 | -1.538813325 |
| Ftsj3 | FtsJ homolog 3 (E. coli) | -1.539663787 |
| Gulp1 | GULP, engulfment adaptor PTB domain containing 1 | -1.540341584 |
| Thbd | thrombomodulin | -1.541190713 |
| Sema6c | sema domain, transmembrane domain (TM), and cytoplasmic domain, (semaphorin) 6C | -1.545722131 |
| Arhgap15 | Rho GTPase activating protein 15 | -1.546749391 |
| Irx6 | iroquois homeobox 6 | -1.552541893 |
| Map3k9 | mitogen-activated protein kinase kinase kinase 9 | -1.552873844 |
| Map4k4 | mitogen-activated protein kinase kinase kinase kinase 4 | -1.558187167 |
| Tsr1 | TSR1, 20S rRNA accumulation, homolog (S. cerevisiae) | -1.559573254 |
| Rwdd4a | RWD domain containing 4A | -1.561999346 |
| Sfrs12 | splicing factor, arginine/serine-rich 12 | -1.565113544 |
| Ehmt1 | euchromatic histone-lysine N-methyltransferase 1 | -1.565336602 |
| Klhl12 | kelch-like 12 (Drosophila) | -1.569222141 |
| Zbt43 | zinc finger and BTB domain containing 43 | -1.572889742 |
| Prdm8 | PR domain containing 8 | -1.581274797 |
| Ly6g6e | lymphocyte antigen 6 complex, locus G6E | -1.582311397 |
| Gna14 | guanine nucleotide binding protein (G protein), alpha 14 | -1.598180617 |
| Gabpb2 | GA binding protein transcription factor, beta subunit 2 | -1.606745891 |
| Uts2 | urotensin 2 | -1.630130045 |
| Chodl | chondrolectin | -1.632742964 |
| Casp9 | caspase 9, apoptosis-related cysteine peptidase | -1.656308589 |
| Il33 | interleukin 33 | -1.672361928 |
| Lhx4 | LIM homeobox 4 | -1.684598114 |
| Rps15a | ribosomal protein S15a | -1.8570344 |
| Sc4mol | sterol-C4-methyl oxidase-like | -2.002597344 |
| Tomm22 | translocase of outer mitochondrial membrane 22 homolog (yeast) | -2.071426334 |
| Bat5 | HLA-B associated transcript 5 | -2.087296671 |
| Enpp5 | ectonucleotide pyrophosphatase/phosphodiesterase 5 (putative function) | -2.320524799 |

**Table S3.** Hypothalamic gene transcripts differentially expressed in running-*ad libitum* C57Bl/6 mice compared to sedentary-*ad libitum* C57Bl/6 mice (n=3 animals per group). Statistical significance was considered at p≤0.05.

| **Gene Smbol** | **Description** | **z-ratio (run vs. sed)** |
| --- | --- | --- |
| Fkbp5 | FK506 binding protein 5 | 4.629585121 |
| Vps72 | vacuolar protein sorting 72 homolog (S. cerevisiae) | 4.113280234 |
| Sh3gl1 | SH3-domain GRB2-like 1 | 3.457982228 |
| Mkx | mohawk homeobox | 3.335088856 |
| Fzd6 | frizzled homolog 6 (Drosophila) | 3.301516918 |
| Fbxo46 | F-box protein 46 | 3.160729768 |
| Lss | lanosterol synthase (2,3-oxidosqualene-lanosterol cyclase) | 3.089629529 |
| Hfe | hemochromatosis | 3.063626572 |
| Fxyd7 | FXYD domain containing ion transport regulator 7 | 3.056303115 |
| Rab3b | RAB3B, member RAS oncogene family | 2.981833231 |
| Adcyap1r1 | adenylate cyclase activating polypeptide 1 (pituitary) receptor type I | 2.980135993 |
| Asb1 | ankyrin repeat and SOCS box-containing 1 | 2.960200544 |
| Rpl21 | ribosomal protein L21 | 2.787825268 |
| Tbl3 | transducin (beta)-like 3 | 2.698130968 |
| AI428936 | chromosome 19 open reading frame 46 | 2.684144791 |
| Slc30a6 | solute carrier family 30 (zinc transporter), member 6 | 2.671221404 |
| Akap8 | A kinase (PRKA) anchor protein 8 | 2.613614844 |
| Npas3 | neuronal PAS domain protein 3 | 2.59188109 |
| Phf7 | PHD finger protein 7 | 2.561755343 |
| Gstm4 | glutathione S-transferase mu 4 | 2.561554665 |
| Polk | polymerase (DNA directed) kappa | 2.552427401 |
| Sesn2 | sestrin 2 | 2.534443895 |
| Pdxk | pyridoxal (pyridoxine, vitamin B6) kinase | 2.530319472 |
| Tor1b | torsin family 1, member B (torsin B) | 2.517340102 |
| Madd | MAP-kinase activating death domain | 2.498494888 |
| Wipf1 | WAS/WASL interacting protein family, member 1 | 2.462488338 |
| Alg12 | asparagine-linked glycosylation 12, alpha-1,6-mannosyltransferase homolog (S. cerevisiae) | 2.418637697 |
| Nif3l1 | NIF3 NGG1 interacting factor 3-like 1 (S. pombe) | 2.410184139 |
| Bbs2 | Bardet-Biedl syndrome 2 | 2.404108333 |
| C2 | complement component 2 | 2.402366462 |
| Dnajb4 | DnaJ (Hsp40) homolog, subfamily B, member 4 | 2.395296014 |
| Tdp1 | tyrosyl-DNA phosphodiesterase 1 | 2.347970205 |
| Klf5 | Kruppel-like factor 5 (intestinal) | 2.34687094 |
| Mycbpap | MYCBP associated protein | 2.342083856 |
| Sfrp1 | secreted frizzled-related protein 1 | 2.324604825 |
| Pdk4 | pyruvate dehydrogenase kinase, isozyme 4 | 2.310998704 |
| Stmn1 | stathmin 1 | 2.276815646 |
| Ccdc9 | coiled-coil domain containing 9 | 2.269303753 |
| Med9 | mediator complex subunit 9 | 2.260922703 |
| Parp14 | poly (ADP-ribose) polymerase family, member 14 | 2.247446996 |
| Arid3a | AT rich interactive domain 3A (BRIGHT-like) | 2.246151151 |
| Aktip | AKT interacting protein | 2.245038144 |
| Prei3 | MOB1, Mps One Binder kinase activator-like 3 (yeast) | 2.233033366 |
| Bmp4 | bone morphogenetic protein 4 | 2.2293231 |
| Ccm2 | cerebral cavernous malformation 2 | 2.221061465 |
| Rbks | ribokinase | 2.209731493 |
| Aste1 | asteroid homolog 1 (Drosophila) | 2.209053051 |
| Kcnn1 | potassium intermediate/small conductance calcium-activated channel, subfamily N, member 1 | 2.200035908 |
| Larp5 | La ribonucleoprotein domain family, member 4B | 2.197649514 |
| Elf1 | E74-like factor 1 (ets domain transcription factor) | 2.172569081 |
| Cad | carbamoyl-phosphate synthetase 2, aspartate transcarbamylase, and dihydroorotase | 2.165957514 |
| Gramd1b | GRAM domain containing 1B | 2.160944648 |
| Pabpc4 | poly(A) binding protein, cytoplasmic 4 (inducible form) | 2.158722113 |
| Fbln2 | fibulin 2 | 2.141528922 |
| Ppat | phosphoribosyl pyrophosphate amidotransferase | 2.139802891 |
| Eno3 | enolase 3 (beta, muscle) | 2.105824579 |
| Tmem143 | transmembrane protein 143 | 2.103713966 |
| Junb | jun B proto-oncogene | 2.103273146 |
| Rspo3 | R-spondin 3 homolog (Xenopus laevis) | 2.100950003 |
| Thada | thyroid adenoma associated | 2.079423324 |
| Ehd1 | EH-domain containing 1 | 2.042265044 |
| Mars | methionyl-tRNA synthetase | 2.020277639 |
| Id4 | inhibitor of DNA binding 4, dominant negative helix-loop-helix protein | 2.011494661 |
| Rnaseh2c | ribonuclease H2, subunit C | 2.00807067 |
| Dennd3 | DENN/MADD domain containing 3 | 2.007834835 |
| Pla2g7 | phospholipase A2, group VII (platelet-activating factor acetylhydrolase, plasma) | 2.002534152 |
| Tmtc4 | transmembrane and tetratricopeptide repeat containing 4 | 1.97125664 |
| Dok4 | docking protein 4 | 1.967071554 |
| BC022687 | chromosome 14 open reading frame 79 | 1.963911403 |
| Lrrc33 | leucine rich repeat containing 33 | 1.95884751 |
| Tgfb2 | transforming growth factor, beta 2 | 1.947264527 |
| Olfml2b | olfactomedin-like 2B | 1.940106982 |
| Polr3a | polymerase (RNA) III (DNA directed) polypeptide A, 155kDa | 1.936615855 |
| Tm6sf1 | transmembrane 6 superfamily member 1 | 1.926865367 |
| Alkbh4 | alkB, alkylation repair homolog 4 (E. coli) | 1.923363729 |
| Vapa | VAMP (vesicle-associated membrane protein)-associated protein A, 33kDa | 1.907004508 |
| BC051142 | cDNA sequence BC051142 | 1.897891703 |
| Rab3d | RAB3D, member RAS oncogene family | 1.895381517 |
| Apip | APAF1 interacting protein | 1.891967488 |
| C1qtnf2 | C1q and tumor necrosis factor related protein 2 | 1.890702544 |
| Rilpl1 | Rab interacting lysosomal protein-like 1 | 1.870970861 |
| Pigl | phosphatidylinositol glycan anchor biosynthesis, class L | 1.85948293 |
| C1galt1c1 | C1GALT1-specific chaperone 1 | 1.850259107 |
| Peli2 | pellino homolog 2 (Drosophila) | 1.849697521 |
| Cadm3 | cell adhesion molecule 3 | 1.849135789 |
| Id2 | inhibitor of DNA binding 2, dominant negative helix-loop-helix protein | 1.826305631 |
| Ang | angiogenin, ribonuclease, RNase A family, 5 | 1.81830736 |
| Dhx37 | DEAH (Asp-Glu-Ala-His) box polypeptide 37 | 1.809035877 |
| Commd1 | copper metabolism (Murr1) domain containing 1 | 1.796837545 |
| Zc3h7a | zinc finger CCCH-type containing 7A | 1.793339373 |
| Lhx1 | LIM homeobox 1 | 1.782908959 |
| Rhebl1 | Ras homolog enriched in brain like 1 | 1.763736338 |
| Odf2 | outer dense fiber of sperm tails 2 | 1.762280933 |
| Qtrt1 | queuine tRNA-ribosyltransferase 1 | 1.754078503 |
| Azin1 | antizyme inhibitor 1 | 1.753971675 |
| Pus3 | pseudouridylate synthase 3 | 1.752001521 |
| Etaa1 | Ewing tumor-associated antigen 1 | 1.751517453 |
| Nfatc2 | nuclear factor of activated T-cells, cytoplasmic, calcineurin-dependent 2 | 1.748776052 |
| Vps37c | vacuolar protein sorting 37 homolog C (S. cerevisiae) | 1.745634212 |
| Cdc42ep3 | CDC42 effector protein (Rho GTPase binding) 3 | 1.741977988 |
| En2 | engrailed homeobox 2 | 1.732908711 |
| Clstn3 | calsyntenin 3 | 1.729128529 |
| Kcnj12 | potassium inwardly-rectifying channel, subfamily J, member 12 | 1.725413045 |
| Hps1 | Hermansky-Pudlak syndrome 1 | 1.724357356 |
| Slc38a9 | solute carrier family 38, member 9 | 1.712453728 |
| Pomt2 | protein-O-mannosyltransferase 2 | 1.709759353 |
| Hdac6 | histone deacetylase 6 | 1.702897543 |
| Zbtb39 | zinc finger and BTB domain containing 39 | 1.700260417 |
| Fhod1 | formin homology 2 domain containing 1 | 1.692000617 |
| Bop1 | block of proliferation 1 | 1.679901138 |
| Ddx5 | DEAD (Asp-Glu-Ala-Asp) box polypeptide 5 | 1.679444874 |
| Pdgfrb | platelet-derived growth factor receptor, beta polypeptide | 1.6724583 |
| Tsn | translin | 1.669999137 |
| Slc6a12 | solute carrier family 6 (neurotransmitter transporter, betaine/GABA), member 12 | 1.664541989 |
| Mapk12 | mitogen-activated protein kinase 12 | 1.660824089 |
| Ccl25 | chemokine (C-C motif) ligand 25 | 1.658068556 |
| Sall1 | sal-like 1 (Drosophila) | 1.655605439 |
| Serpind1 | serpin peptidase inhibitor, clade D (heparin cofactor), member 1 | 1.650268959 |
| Spata7 | spermatogenesis associated 7 | 1.648055932 |
| Cpeb1 | cytoplasmic polyadenylation element binding protein 1 | 1.646928804 |
| Dhx8 | DEAH (Asp-Glu-Ala-His) box polypeptide 8 | 1.64319646 |
| Tmcc3 | transmembrane and coiled-coil domain family 3 | 1.638144587 |
| Zbtb5 | zinc finger and BTB domain containing 5 | 1.637302857 |
| Pcnx | pecanex homolog (Drosophila) | 1.636912753 |
| Tex264 | testis expressed 264 | 1.635944023 |
| Kremen2 | kringle containing transmembrane protein 2 | 1.634813704 |
| Iqsec2 | IQ motif and Sec7 domain 2 | 1.628722243 |
| Clgn | calmegin | 1.62767629 |
| Grin3b | glutamate receptor, ionotropic, N-methyl-D-aspartate 3B | 1.624943139 |
| Itpka | inositol 1,4,5-trisphosphate 3-kinase A | 1.620874897 |
| Ntsr1 | neurotensin receptor 1 (high affinity) | 1.614787407 |
| Nqo2 | NAD(P)H dehydrogenase, quinone 2 | 1.606561106 |
| Crocc | ciliary rootlet coiled-coil, rootletin | 1.604324789 |
| Caly | calcyon neuron-specific vesicular protein | 1.597550464 |
| Dynlrb2 | dynein, light chain, roadblock-type 2 | 1.583864953 |
| Rapgef3 | Rap guanine nucleotide exchange factor (GEF) 3 | 1.58090538 |
| Gja4 | gap junction protein, alpha 4, 37kDa | 1.574760244 |
| Ylpm1 | YLP motif containing 1 | 1.574691339 |
| Arsj | arylsulfatase family, member J | 1.566209051 |
| Atp6v1b2 | ATPase, H+ transporting, lysosomal 56/58kDa, V1 subunit B2 | 1.563555067 |
| Incenp | inner centromere protein antigens 135/155kDa | 1.557027251 |
| Foxc1 | forkhead box C1 | 1.553014049 |
| Htra3 | HtrA serine peptidase 3 | 1.544945743 |
| Yod1 | YOD1 OTU deubiquinating enzyme 1 homolog (S. cerevisiae) | 1.544616169 |
| Dot1l | DOT1-like, histone H3 methyltransferase (S. cerevisiae) | 1.544460115 |
| Kcnk2 | potassium channel, subfamily K, member 2 | 1.537861849 |
| Ctr9 | Ctr9, Paf1/RNA polymerase II complex component, homolog (S. cerevisiae) | 1.534242443 |
| Tle6 | transducin-like enhancer of split 6 (E(sp1) homolog, Drosophila) | 1.534140197 |
| Ppil2 | peptidylprolyl isomerase (cyclophilin)-like 2 | 1.533765409 |
| Chrd | chordin | 1.528942606 |
| Nedd4 | neural precursor cell expressed, developmentally down-regulated 4 | 1.527802855 |
| Ankrd24 | ankyrin repeat domain 24 | 1.523016523 |
| AK122209 | URB2 ribosome biogenesis 2 homolog (S. cerevisiae) | 1.514279583 |
| U2af2 | U2 small nuclear RNA auxiliary factor 2 | 1.512465202 |
| Nme7 | non-metastatic cells 7, protein expressed in (nucleoside-diphosphate kinase) | 1.507671961 |
| Rai14 | retinoic acid induced 14 | 1.505727608 |
| Dgcr8 | DiGeorge syndrome critical region gene 8 | 1.501935129 |
| Tdrd6 | tudor domain containing 6 | 1.501642155 |
| Uroc1 | urocanase domain containing 1 | -1.500273865 |
| Wnt10a | wingless-type MMTV integration site family, member 10A | -1.500441564 |
| Dnmbp | dynamin binding protein | -1.500543767 |
| Ccdc44 | coiled-coil domain containing 44 | -1.500689705 |
| Hsd17b2 | hydroxysteroid (17-beta) dehydrogenase 2 | -1.501385247 |
| Foxp3 | forkhead box P3 | -1.502436714 |
| Apbb1ip | amyloid beta (A4) precursor protein-binding, family B, member 1 interacting protein | -1.503479732 |
| Slc30a3 | solute carrier family 30 (zinc transporter), member 3 | -1.504090329 |
| Morn3 | MORN repeat containing 3 | -1.504540785 |
| Kdr | kinase insert domain receptor (a type III receptor tyrosine kinase) | -1.504708361 |
| Wdr41 | WD repeat domain 41 | -1.50477045 |
| Adck2 | aarF domain containing kinase 2 | -1.504800845 |
| Irak1bp1 | interleukin-1 receptor-associated kinase 1 binding protein 1 | -1.505499392 |
| Ppfibp1 | PTPRF interacting protein, binding protein 1 (liprin beta 1) | -1.505618864 |
| Angptl1 | angiopoietin-like 1 | -1.505893479 |
| Zdhhc18 | zinc finger, DHHC-type containing 18 | -1.506656781 |
| Zfyve19 | zinc finger, FYVE domain containing 19 | -1.508270063 |
| Chm | choroideremia (Rab escort protein 1) | -1.508635394 |
| Eaf2 | ELL associated factor 2 | -1.508637191 |
| F5 | coagulation factor V (proaccelerin, labile factor) | -1.508682493 |
| Ptprm | protein tyrosine phosphatase, receptor type, M | -1.509108177 |
| Vash1 | vasohibin 1 | -1.509432611 |
| BC032265 | fructosamine 3 kinase related protein | -1.509617458 |
| Baz1b | bromodomain adjacent to zinc finger domain, 1B | -1.509952601 |
| Spag4 | sperm associated antigen 4 | -1.510465355 |
| Mbd6 | methyl-CpG binding domain protein 6 | -1.51060789 |
| Btbd16 | BTB (POZ) domain containing 16 | -1.511184042 |
| Ela2 | elastase, neutrophil expressed | -1.511319249 |
| Mfsd4 | major facilitator superfamily domain containing 4 | -1.511491203 |
| Accn3 | amiloride-sensitive cation channel 3 | -1.511648911 |
| Treml1 | triggering receptor expressed on myeloid cells-like 1 | -1.512629573 |
| Tmtc1 | transmembrane and tetratricopeptide repeat containing 1 | -1.513505011 |
| Polm | polymerase (DNA directed), mu | -1.51396243 |
| Kcnj9 | potassium inwardly-rectifying channel, subfamily J, member 9 | -1.514383301 |
| Socs3 | suppressor of cytokine signaling 3 | -1.514430966 |
| Bmp2k | BMP2 inducible kinase | -1.51474952 |
| BC030500 | cDNA sequence BC030500 | -1.515344054 |
| Kcne4 | potassium voltage-gated channel, Isk-related family, member 4 | -1.516114857 |
| Tead4 | TEA domain family member 4 | -1.51625211 |
| Pla2g4e | phospholipase A2, group IVE | -1.516459556 |
| She | Src homology 2 domain containing E | -1.516500559 |
| Avil | advillin | -1.516502196 |
| Lcp1 | lymphocyte cytosolic protein 1 (L-plastin) | -1.516780214 |
| Wdr42a | WD repeat domain 42A | -1.517715483 |
| Lmx1a | LIM homeobox transcription factor 1, alpha | -1.518031194 |
| Cgnl1 | cingulin-like 1 | -1.518711104 |
| Rnf139 | ring finger protein 139 | -1.518967443 |
| Tnrc18 | trinucleotide repeat containing 18 | -1.519075628 |
| Onecut3 | one cut homeobox 3 | -1.519227513 |
| Kcnk5 | potassium channel, subfamily K, member 5 | -1.51999833 |
| Paxip1 | PAX interacting (with transcription-activation domain) protein 1 | -1.520521633 |
| Asb9 | ankyrin repeat and SOCS box-containing 9 | -1.520923362 |
| Ccdc64 | coiled-coil domain containing 64 | -1.520928756 |
| Htr2a | 5-hydroxytryptamine (serotonin) receptor 2A | -1.521487096 |
| Tbx22 | T-box 22 | -1.521733061 |
| Scgn | secretagogin, EF-hand calcium binding protein | -1.521829179 |
| Lep | leptin | -1.522720664 |
| Fhit | fragile histidine triad gene | -1.524361392 |
| Apoc3 | apolipoprotein C-III | -1.524772618 |
| BC060267 | chromosome 3 open reading frame 25 | -1.524854971 |
| Pdcl3 | phosducin-like 3 | -1.52552732 |
| Slc4a1 | solute carrier family 4, anion exchanger, member 1 | -1.525774605 |
| Adamts1 | ADAM metallopeptidase with thrombospondin type 1 motif, 1 | -1.52641813 |
| Lrp6 | low density lipoprotein receptor-related protein 6 | -1.52649401 |
| Acyp1 | acylphosphatase 1, erythrocyte (common) type | -1.526872634 |
| Tmem82 | transmembrane protein 82 | -1.527418823 |
| Lass1 | LAG1 homolog, ceramide synthase 1 | -1.52920926 |
| Krt82 | keratin 82 | -1.5292426 |
| Rbp7 | retinol binding protein 7, cellular | -1.5292808 |
| Tbc1d8b | TBC1 domain family, member 8B (with GRAM domain) | -1.529699559 |
| Lrrc36 | leucine rich repeat containing 36 | -1.530029162 |
| Tbx6 | T-box 6 | -1.530900431 |
| Thg1l | tRNA-histidine guanylyltransferase 1-like (S. cerevisiae) | -1.531192228 |
| Ppef2 | protein phosphatase, EF-hand calcium binding domain 2 | -1.531480387 |
| Akap3 | A kinase (PRKA) anchor protein 3 | -1.531796736 |
| Il23a | interleukin 23, alpha subunit p19 | -1.532830824 |
| Fndc3a | fibronectin type III domain containing 3A | -1.533637993 |
| Cyp2u1 | cytochrome P450, family 2, subfamily U, polypeptide 1 | -1.533747688 |
| Abca4 | ATP-binding cassette, sub-family A (ABC1), member 4 | -1.53394315 |
| Entpd6 | ectonucleoside triphosphate diphosphohydrolase 6 (putative function) | -1.53442001 |
| Slc10a7 | solute carrier family 10 (sodium/bile acid cotransporter family), member 7 | -1.534628486 |
| Traf3ip1 | TNF receptor-associated factor 3 interacting protein 1 | -1.534715617 |
| Cnksr3 | CNKSR family member 3 | -1.535926688 |
| S100a8 | S100 calcium binding protein A8 | -1.536122348 |
| Papln | papilin, proteoglycan-like sulfated glycoprotein | -1.536241248 |
| Liph | lipase, member H | -1.536304788 |
| Ly9 | lymphocyte antigen 9 | -1.536663724 |
| Phf20l1 | PHD finger protein 20-like 1 | -1.537014383 |
| Aire | autoimmune regulator | -1.537034126 |
| Fxyd2 | FXYD domain containing ion transport regulator 2 | -1.537301169 |
| Phka1 | phosphorylase kinase, alpha 1 (muscle) | -1.537467851 |
| Vash2 | vasohibin 2 | -1.537943737 |
| Clcc1 | chloride channel CLIC-like 1 | -1.538120038 |
| Rbpjl | recombination signal binding protein for immunoglobulin kappa J region-like | -1.538289806 |
| Cenpn | centromere protein N | -1.53837344 |
| Ugt1a10 | UDP glucuronosyltransferase 1 family, polypeptide A10 | -1.538474217 |
| Kif5b | kinesin family member 5B | -1.539166508 |
| Wnt9b | wingless-type MMTV integration site family, member 9B | -1.540304911 |
| Notum | notum pectinacetylesterase homolog (Drosophila) | -1.541319803 |
| Anxa4 | annexin A4 | -1.54182317 |
| Idi2 | isopentenyl-diphosphate delta isomerase 2 | -1.543208094 |
| Wasf2 | WAS protein family, member 2 | -1.543489656 |
| Mllt10 | myeloid/lymphoid or mixed-lineage leukemia (trithorax homolog, Drosophila); translocated to, 10 | -1.543679018 |
| Nms | neuromedin S | -1.544294035 |
| BC089491 | selenoprotein V | -1.54448134 |
| Stac3 | SH3 and cysteine rich domain 3 | -1.544997535 |
| Trim40 | tripartite motif-containing 40 | -1.545229344 |
| Csf3 | colony stimulating factor 3 (granulocyte) | -1.545276382 |
| Fscn2 | fascin homolog 2, actin-bundling protein, retinal (Strongylocentrotus purpuratus) | -1.545325093 |
| Tnfrsf10b | tumor necrosis factor receptor superfamily, member 10b | -1.545823703 |
| Mb | myoglobin | -1.546808615 |
| Htr1b | 5-hydroxytryptamine (serotonin) receptor 1B | -1.547166062 |
| Abhd1 | abhydrolase domain containing 1 | -1.548053268 |
| Zfp92 | zinc finger protein 92 homolog (mouse) | -1.548571433 |
| Mfsd7 | major facilitator superfamily domain containing 7 | -1.54900256 |
| Gnb3 | guanine nucleotide binding protein (G protein), beta polypeptide 3 | -1.549341011 |
| Papss2 | 3'-phosphoadenosine 5'-phosphosulfate synthase 2 | -1.549520224 |
| Lifr | leukemia inhibitory factor receptor alpha | -1.550248537 |
| Pde3a | phosphodiesterase 3A, cGMP-inhibited | -1.551270037 |
| Rln1 | relaxin 1 | -1.553271135 |
| Mfap4 | microfibrillar-associated protein 4 | -1.553844339 |
| Egr2 | early growth response 2 | -1.554188082 |
| Il2rb | interleukin 2 receptor, beta | -1.554357886 |
| Gyltl1b | glycosyltransferase-like 1B | -1.555386065 |
| Tm6sf2 | transmembrane 6 superfamily member 2 | -1.556627007 |
| Sstr5 | somatostatin receptor 5 | -1.558295772 |
| Ninj1 | ninjurin 1 | -1.559160496 |
| Slc22a18 | solute carrier family 22, member 18 | -1.560258239 |
| Slc14a2 | solute carrier family 14 (urea transporter), member 2 | -1.562616739 |
| Pgr | progesterone receptor | -1.562648746 |
| Irak4 | interleukin-1 receptor-associated kinase 4 | -1.564032688 |
| Acad8 | acyl-Coenzyme A dehydrogenase family, member 8 | -1.565698621 |
| Tsnaxip1 | translin-associated factor X interacting protein 1 | -1.566101463 |
| Agrp | agouti related protein homolog (mouse) | -1.566240356 |
| Kif17 | kinesin family member 17 | -1.567028043 |
| Slit1 | slit homolog 1 (Drosophila) | -1.567051103 |
| Hlf | hepatic leukemia factor | -1.567508001 |
| Pde8b | phosphodiesterase 8B | -1.567674858 |
| AI842396 | inhibitor of CDK, cyclin A1 interacting protein 1 | -1.569216642 |
| Gstcd | glutathione S-transferase, C-terminal domain containing | -1.569287829 |
| Pou3f3 | POU class 3 homeobox 3 | -1.570698077 |
| Avp | arginine vasopressin | -1.571612595 |
| Ctsl | cathepsin L1 | -1.572183373 |
| Nat2 | N-acetyltransferase 2 (arylamine N-acetyltransferase) | -1.574508868 |
| Hdac9 | histone deacetylase 9 | -1.574640643 |
| Gprc5c | G protein-coupled receptor, family C, group 5, member C | -1.575611329 |
| Slc2a4 | solute carrier family 2 (facilitated glucose transporter), member 4 | -1.576650191 |
| Jmjd2a | lysine (K)-specific demethylase 4A | -1.577539086 |
| Cabyr | calcium binding tyrosine-(Y)-phosphorylation regulated | -1.578551605 |
| Klhl15 | kelch-like 15 (Drosophila) | -1.578585064 |
| Nkx2-1 | NK2 homeobox 1 | -1.579135919 |
| Rlbp1l1 | retinaldehyde binding protein 1-like 1 | -1.579745064 |
| Ces3 | carboxylesterase 3 | -1.579856411 |
| Ripk5 | dual serine/threonine and tyrosine protein kinase | -1.580008861 |
| Lrfn1 | leucine rich repeat and fibronectin type III domain containing 1 | -1.580788102 |
| Il20rb | interleukin 20 receptor beta | -1.583255847 |
| Nup54 | nucleoporin 54kDa | -1.58423682 |
| Pqlc3 | PQ loop repeat containing 3 | -1.585429025 |
| Crygn | crystallin, gamma N | -1.586250936 |
| Matn1 | matrilin 1, cartilage matrix protein | -1.588235964 |
| Usp37 | ubiquitin specific peptidase 37 | -1.588242285 |
| Ccdc79 | coiled-coil domain containing 79 | -1.58974343 |
| Adam21 | ADAM metallopeptidase domain 21 | -1.590912169 |
| Mybl2 | v-myb myeloblastosis viral oncogene homolog (avian)-like 2 | -1.593014514 |
| Ccdc99 | coiled-coil domain containing 99 | -1.594214118 |
| Rxra | retinoid X receptor, alpha | -1.594732242 |
| Tmod4 | tropomodulin 4 (muscle) | -1.594836711 |
| Fbxl21 | F-box and leucine-rich repeat protein 21 | -1.595387826 |
| Hsf5 | heat shock transcription factor family member 5 | -1.596755847 |
| Mtf1 | metal-regulatory transcription factor 1 | -1.59712482 |
| Ablim3 | actin binding LIM protein family, member 3 | -1.598386765 |
| Adamts18 | ADAM metallopeptidase with thrombospondin type 1 motif, 18 | -1.598449767 |
| Wasf3 | WAS protein family, member 3 | -1.599724767 |
| Catsper3 | cation channel, sperm associated 3 | -1.599811317 |
| Wfdc12 | WAP four-disulfide core domain 12 | -1.599892633 |
| Sf3b4 | splicing factor 3b, subunit 4, 49kDa | -1.601143987 |
| Krt17 | keratin 17 | -1.601407892 |
| Syt14 | synaptotagmin XIV | -1.604689774 |
| Dnali1 | dynein, axonemal, light intermediate chain 1 | -1.607534006 |
| Scube2 | signal peptide, CUB domain, EGF-like 2 | -1.609130993 |
| Gramd1a | GRAM domain containing 1A | -1.610410478 |
| Spag8 | sperm associated antigen 8 | -1.610559355 |
| Slc37a1 | solute carrier family 37 (glycerol-3-phosphate transporter), member 1 | -1.611592289 |
| Slc38a8 | solute carrier family 38, member 8 | -1.612007582 |
| Usp9x | ubiquitin specific peptidase 9, X-linked | -1.612174449 |
| Tbc1d14 | TBC1 domain family, member 14 | -1.614654965 |
| Leng8 | leukocyte receptor cluster (LRC) member 8 | -1.615114815 |
| Havcr1 | hepatitis A virus cellular receptor 1 | -1.616268218 |
| Hoxd11 | homeobox D11 | -1.618277005 |
| Itgad | integrin, alpha D | -1.621176006 |
| Tpmt | thiopurine S-methyltransferase | -1.622391915 |
| Nppa | natriuretic peptide precursor A | -1.625342513 |
| Pla2g2d | phospholipase A2, group IID | -1.627427279 |
| Lyg1 | lysozyme G-like 1 | -1.632212321 |
| Tm4sf5 | transmembrane 4 L six family member 5 | -1.635005925 |
| Mapk13 | mitogen-activated protein kinase 13 | -1.638725495 |
| Wnt6 | wingless-type MMTV integration site family, member 6 | -1.655786998 |
| Anxa9 | annexin A9 | -1.656677484 |
| Tle3 | transducin-like enhancer of split 3 (E(sp1) homolog, Drosophila) | -1.675730111 |
| Vax1 | ventral anterior homeobox 1 | -1.676345836 |
| Txndc9 | thioredoxin domain containing 9 | -1.677459383 |
| Camp | cathelicidin antimicrobial peptide | -1.69028464 |
| Ccl17 | chemokine (C-C motif) ligand 17 | -1.695378322 |
| S100a9 | S100 calcium binding protein A9 | -1.697428106 |
| Gzmk | granzyme K (granzyme 3; tryptase II) | -1.699853331 |
| B3gat2 | beta-1,3-glucuronyltransferase 2 (glucuronosyltransferase S) | -1.705173296 |
| Ccr6 | chemokine (C-C motif) receptor 6 | -1.707864733 |
| Mov10 | Mov10, Moloney leukemia virus 10, homolog (mouse) | -1.714101366 |
| Arl10 | ADP-ribosylation factor-like 10 | -1.737292193 |
| Ltf | lactotransferrin | -1.754295747 |
| Gsn | gelsolin (amyloidosis, Finnish type) | -1.76256815 |
| Trim72 | tripartite motif-containing 72 | -1.782425324 |
| Hcrt | hypocretin (orexin) neuropeptide precursor | -1.806632805 |
| Lcn2 | lipocalin 2 | -1.879275114 |

**Table S4.** Hypothalamic gene transcripts differentially expressed in sedentary-caloric restriction (CR) C57Bl/6 mice compared to sedentary-*ad libitum* (AL) C57Bl/6 mice (n=3 animals per group). Statistical significance was considered at p≤0.05.

| **Gene Symbol** | **Description** | **z-ratio (CR vs. AL)** |
| --- | --- | --- |
| Otof | otoferlin | 3.665366906 |
| Ylpm1 | YLP motif containing 1 | 3.649334373 |
| Wipf1 | WAS/WASL interacting protein family, member 1 | 3.619024404 |
| Hip1 | huntingtin interacting protein 1 | 3.570941932 |
| Narg2 | NMDA receptor regulated 2 | 3.555626294 |
| Polk | polymerase (DNA directed) kappa | 3.533718249 |
| Med1 | mediator complex subunit 1 | 3.513780245 |
| Dnaja2 | DnaJ (Hsp40) homolog, subfamily A, member 2 | 3.504038779 |
| Nr6a1 | nuclear receptor subfamily 6, group A, member 1 | 3.464526861 |
| Smc5l1 | structural maintenance of chromosomes 5 | 3.444511519 |
| Kpnb1 | karyopherin (importin) beta 1 | 3.398280159 |
| Zcwpw1 | zinc finger, CW type with PWWP domain 1 | 3.396743507 |
| Asb1 | ankyrin repeat and SOCS box-containing 1 | 3.379216608 |
| Slc27a3 | solute carrier family 27 (fatty acid transporter), member 3 | 3.326439452 |
| Mettl6 | methyltransferase like 6 | 3.308676373 |
| Abi3 | ABI family, member 3 | 3.307275973 |
| Pgrmc1 | progesterone receptor membrane component 1 | 3.304147861 |
| Rfwd2 | ring finger and WD repeat domain 2 | 3.299368784 |
| Tspan12 | tetraspanin 12 | 3.29931342 |
| L3mbtl2 | l(3)mbt-like 2 (Drosophila) | 3.294574757 |
| Apbb3 | amyloid beta (A4) precursor protein-binding, family B, member 3 | 3.290771413 |
| BC016423 | chromosome 10 open reading frame 18 | 3.277899114 |
| Stmn1 | stathmin 1 | 3.277574984 |
| Rbm19 | RNA binding motif protein 19 | 3.276856394 |
| Cog2 | component of oligomeric golgi complex 2 | 3.251949553 |
| Pdk4 | pyruvate dehydrogenase kinase, isozyme 4 | 3.215373752 |
| Srp19 | signal recognition particle 19kDa | 3.213449306 |
| Polr3k | polymerase (RNA) III (DNA directed) polypeptide K, 12.3 kDa | 3.200781497 |
| Cic | capicua homolog (Drosophila) | 3.164348557 |
| Cycs | cytochrome c, somatic | 3.163067727 |
| Kcns2 | potassium voltage-gated channel, delayed-rectifier, subfamily S, member 2 | 3.147315743 |
| Pdss1 | prenyl (decaprenyl) diphosphate synthase, subunit 1 | 3.132509496 |
| Pou4f1 | POU class 4 homeobox 1 | 3.127294586 |
| Id4 | inhibitor of DNA binding 4, dominant negative helix-loop-helix protein | 3.115187389 |
| Jag2 | jagged 2 | 3.10867181 |
| Wdr5 | WD repeat domain 5 | 3.084238996 |
| Recql5 | RecQ protein-like 5 | 3.082974729 |
| Tes | testis derived transcript (3 LIM domains) | 3.067303709 |
| Npy5r | neuropeptide Y receptor Y5 | 3.06547801 |
| Bop1 | block of proliferation 1 | 3.057642153 |
| Akap8 | A kinase (PRKA) anchor protein 8 | 3.038282939 |
| Ccdc91 | coiled-coil domain containing 91 | 2.980231717 |
| Dhx33 | DEAH (Asp-Glu-Ala-His) box polypeptide 33 | 2.960243496 |
| Rft1 | RFT1 homolog (S. cerevisiae) | 2.947407575 |
| Stab1 | stabilin 1 | 2.91003136 |
| Rragd | Ras-related GTP binding D | 2.88303204 |
| Rab11a | RAB11A, member RAS oncogene family | 2.862956307 |
| Slc23a2 | solute carrier family 23 (nucleobase transporters), member 2 | 2.847406961 |
| Fzd6 | frizzled homolog 6 (Drosophila) | 2.842651888 |
| Etv4 | ets variant 4 | 2.817162326 |
| Pdxk | pyridoxal (pyridoxine, vitamin B6) kinase | 2.805557379 |
| M6prbp1 | mannose-6-phosphate receptor binding protein 1 | 2.799195424 |
| Ebf1 | early B-cell factor 1 | 2.781326582 |
| Slain2 | SLAIN motif family, member 2 | 2.778248862 |
| Btg4 | B-cell translocation gene 4 | 2.777339488 |
| Stag1 | stromal antigen 1 | 2.774193027 |
| BC067068 | chromosome 12 open reading frame 26 | 2.752601793 |
| Commd8 | COMM domain containing 8 | 2.744464637 |
| Aqr | aquarius homolog (mouse) | 2.736879973 |
| Atpbd3 | ATP binding domain 3 | 2.732095378 |
| Mansc1 | MANSC domain containing 1 | 2.720520592 |
| Tnfaip8 | tumor necrosis factor, alpha-induced protein 8 | 2.719350381 |
| Tor1b | torsin family 1, member B (torsin B) | 2.700814741 |
| Setx | senataxin | 2.698131916 |
| Sox9 | SRY (sex determining region Y)-box 9 | 2.697727771 |
| BC020002 | missing oocyte, meiosis regulator, homolog (Drosophila) | 2.688331999 |
| Chpf | chondroitin polymerizing factor | 2.686598075 |
| BC088983 | KIAA1712 | 2.674773715 |
| Bet1 | blocked early in transport 1 homolog (S. cerevisiae) | 2.673486698 |
| Llgl2 | lethal giant larvae homolog 2 (Drosophila) | 2.660204961 |
| Rbpms2 | RNA binding protein with multiple splicing 2 | 2.657430682 |
| Hdac6 | histone deacetylase 6 | 2.651994499 |
| Fbxl5 | F-box and leucine-rich repeat protein 5 | 2.650091382 |
| St3gal2 | ST3 beta-galactoside alpha-2,3-sialyltransferase 2 | 2.625622005 |
| Stk32b | serine/threonine kinase 32B | 2.608139582 |
| Slc30a6 | solute carrier family 30 (zinc transporter), member 6 | 2.604430804 |
| Rrn3 | RRN3 RNA polymerase I transcription factor homolog (S. cerevisiae) | 2.602760891 |
| Bbs2 | Bardet-Biedl syndrome 2 | 2.601178228 |
| Lss | lanosterol synthase (2,3-oxidosqualene-lanosterol cyclase) | 2.599981647 |
| Cbln2 | cerebellin 2 precursor | 2.594485684 |
| BC022687 | chromosome 14 open reading frame 79 | 2.589566262 |
| Twistnb | TWIST neighbor | 2.584655682 |
| Cyfip2 | cytoplasmic FMR1 interacting protein 2 | 2.577976697 |
| Idh2 | isocitrate dehydrogenase 2 (NADP+), mitochondrial | 2.565042892 |
| Pibf1 | progesterone immunomodulatory binding factor 1 | 2.557471366 |
| Hps6 | Hermansky-Pudlak syndrome 6 | 2.556248565 |
| Ehd1 | EH-domain containing 1 | 2.551889525 |
| Rab10 | RAB10, member RAS oncogene family | 2.478702936 |
| Zc3h7a | zinc finger CCCH-type containing 7A | 2.466868899 |
| Htr5b | 5-hydroxytryptamine (serotonin) receptor 5B | 2.447947028 |
| Pla2g7 | phospholipase A2, group VII (platelet-activating factor acetylhydrolase, plasma) | 2.428305735 |
| Prmt8 | protein arginine methyltransferase 8 | 2.427970771 |
| Lrrc6 | leucine rich repeat containing 6 | 2.423923942 |
| Bbs9 | Bardet-Biedl syndrome 9 | 2.419455697 |
| Tpp2 | tripeptidyl peptidase II | 2.394471712 |
| Cav1 | caveolin 1, caveolae protein, 22kDa | 2.387464629 |
| Id2 | inhibitor of DNA binding 2, dominant negative helix-loop-helix protein | 2.383329352 |
| Dok4 | docking protein 4 | 2.380650229 |
| Camk1d | calcium/calmodulin-dependent protein kinase ID | 2.378521375 |
| Cdyl | chromodomain protein, Y-like | 2.344980751 |
| Fastkd1 | FAST kinase domains 1 | 2.335737919 |
| Aph1b | anterior pharynx defective 1 homolog B (C. elegans) | 2.3275881 |
| Gan | gigaxonin | 2.325088575 |
| Cox7a2 | cytochrome c oxidase subunit VIIa polypeptide 2 (liver) | 2.321743085 |
| Ube3a | ubiquitin protein ligase E3A | 2.318485265 |
| Tsn | translin | 2.310765744 |
| Fyco1 | FYVE and coiled-coil domain containing 1 | 2.304705628 |
| Setd8 | SET domain containing (lysine methyltransferase) 8 | 2.2924515 |
| Npb | neuropeptide B | 2.283895652 |
| Centa2 | ArfGAP with dual PH domains 2 | 2.281959453 |
| U2af2 | U2 small nuclear RNA auxiliary factor 2 | 2.27309304 |
| BC005537 | chromosome 6 open reading frame 62 | 2.246026638 |
| Tmem176a | transmembrane protein 176A | 2.23459123 |
| Txndc12 | thioredoxin domain containing 12 (endoplasmic reticulum) | 2.234456759 |
| Cdc42ep3 | CDC42 effector protein (Rho GTPase binding) 3 | 2.227639211 |
| Mrpl35 | mitochondrial ribosomal protein L35 | 2.21570693 |
| Csgalnact1 | chondroitin sulfate N-acetylgalactosaminyltransferase 1 | 2.191473511 |
| Twist1 | twist homolog 1 (Drosophila) | 2.181610819 |
| Senp5 | SUMO1/sentrin specific peptidase 5 | 2.179739695 |
| Nr4a2 | nuclear receptor subfamily 4, group A, member 2 | 2.177920485 |
| Ccng2 | cyclin G2 | 2.160777749 |
| Phtf2 | putative homeodomain transcription factor 2 | 2.156701381 |
| Ddx19b | DEAD (Asp-Glu-Ala-As) box polypeptide 19B | 2.154700951 |
| Mrc1 | mannose receptor, C type 1 | 2.152737548 |
| Kctd3 | potassium channel tetramerisation domain containing 3 | 2.148717097 |
| Zan | zonadhesin | 2.143898517 |
| Ncbp2 | nuclear cap binding protein subunit 2, 20kDa | 2.139913836 |
| Pdgfrb | platelet-derived growth factor receptor, beta polypeptide | 2.136703519 |
| Trhr | thyrotropin-releasing hormone receptor | 2.114365813 |
| Mettl4 | methyltransferase like 4 | 2.112102438 |
| Npc2 | Niemann-Pick disease, type C2 | 2.111348789 |
| Pfn4 | profilin family, member 4 | 2.093834783 |
| Slc35f1 | solute carrier family 35, member F1 | 2.066237973 |
| Cdc34 | cell division cycle 34 homolog (S. cerevisiae) | 2.062511804 |
| Ythdf3 | YTH domain family, member 3 | 2.054800522 |
| Ccdc9 | coiled-coil domain containing 9 | 2.03859638 |
| Nt5e | 5'-nucleotidase, ecto (CD73) | 2.025528546 |
| Hk1 | hexokinase 1 | 2.017894179 |
| Cat | catalase | 2.017749745 |
| Rhebl1 | Ras homolog enriched in brain like 1 | 2.016261428 |
| Ehbp1 | EH domain binding protein 1 | 2.011073689 |
| Lman1 | lectin, mannose-binding, 1 | 1.991016811 |
| Rasip1 | Ras interacting protein 1 | 1.98951045 |
| Matn4 | matrilin 4 | 1.987826278 |
| Kirrel3 | kin of IRRE like 3 (Drosophila) | 1.984907417 |
| Nhlrc2 | NHL repeat containing 2 | 1.973580852 |
| Nptx1 | neuronal pentraxin I | 1.969040585 |
| Xrcc6 | X-ray repair complementing defective repair in Chinese hamster cells 6 | 1.966584985 |
| Ndufb9 | NADH dehydrogenase (ubiquinone) 1 beta subcomplex, 9, 22kDa | 1.947988794 |
| Vps36 | vacuolar protein sorting 36 homolog (S. cerevisiae) | 1.941802917 |
| Clk1 | CDC-like kinase 1 | 1.940614415 |
| Kin | KIN, antigenic determinant of recA protein homolog (mouse) | 1.926546463 |
| Lrsam1 | leucine rich repeat and sterile alpha motif containing 1 | 1.922416153 |
| Ccl25 | chemokine (C-C motif) ligand 25 | 1.918112428 |
| Aktip | AKT interacting protein | 1.914847668 |
| Man2b2 | mannosidase, alpha, class 2B, member 2 | 1.912010227 |
| Sox17 | SRY (sex determining region Y)-box 17 | 1.901775769 |
| AA691260 | low density lipoprotein receptor adaptor protein 1 | 1.899971653 |
| Calcoco1 | calcium binding and coiled-coil domain 1 | 1.895293261 |
| Acadsb | acyl-Coenzyme A dehydrogenase, short/branched chain | 1.890942421 |
| Net1 | neuroepithelial cell transforming 1 | 1.890241851 |
| Hif3a | hypoxia inducible factor 3, alpha subunit | 1.882601934 |
| Olfm2 | olfactomedin 2 | 1.881772961 |
| Pbx4 | pre-B-cell leukemia homeobox 4 | 1.871631045 |
| Nudt12 | nudix (nucleoside diphosphate linked moiety X)-type motif 12 | 1.861929697 |
| Nuak2 | NUAK family, SNF1-like kinase, 2 | 1.859181717 |
| Dll1 | delta-like 1 (Drosophila) | 1.856925836 |
| H6pd | hexose-6-phosphate dehydrogenase (glucose 1-dehydrogenase) | 1.852110147 |
| Dcun1d5 | DCN1, defective in cullin neddylation 1, domain containing 5 (S. cerevisiae) | 1.844686437 |
| Casp1 | caspase 1, apoptosis-related cysteine peptidase (interleukin 1, beta, convertase) | 1.84284613 |
| Ntrk3 | neurotrophic tyrosine kinase, receptor, type 3 | 1.835664931 |
| Hist1h3d | histone cluster 1, H3d | 1.834906017 |
| Vbp1 | von Hippel-Lindau binding protein 1 | 1.834704458 |
| Elavl4 | ELAV (embryonic lethal, abnormal vision, Drosophila)-like 4 (Hu antigen D) | 1.830808695 |
| Rap2b | RAP2B, member of RAS oncogene family | 1.82810031 |
| Emg1 | EMG1 nucleolar protein homolog (S. cerevisiae) | 1.827263222 |
| Agpat9 | 1-acylglycerol-3-phosphate O-acyltransferase 9 | 1.821936152 |
| Foxo6 | forkhead box protein O6 | 1.820271367 |
| Prkcq | protein kinase C, theta | 1.814641917 |
| Dleu7 | deleted in lymphocytic leukemia, 7 | 1.811212931 |
| Tmem178 | transmembrane protein 178 | 1.80869672 |
| Arntl | aryl hydrocarbon receptor nuclear translocator-like | 1.803659025 |
| Gucy1a3 | guanylate cyclase 1, soluble, alpha 3 | 1.802574902 |
| Olfml2b | olfactomedin-like 2B | 1.795173567 |
| Faim | Fas apoptotic inhibitory molecule | 1.794502681 |
| Grb14 | growth factor receptor-bound protein 14 | 1.790530007 |
| Kremen2 | kringle containing transmembrane protein 2 | 1.788153282 |
| Prpf40a | PRP40 pre-mRNA processing factor 40 homolog A (S. cerevisiae) | 1.780573211 |
| Huwe1 | HECT, UBA and WWE domain containing 1 | 1.777643583 |
| Rbm12 | RNA binding motif protein 12 | 1.775214175 |
| Pdik1l | PDLIM1 interacting kinase 1 like | 1.771549673 |
| Spred2 | sprouty-related, EVH1 domain containing 2 | 1.759953046 |
| Inhba | inhibin, beta A | 1.759459613 |
| Lrrk2 | leucine-rich repeat kinase 2 | 1.752248157 |
| Efnb1 | ephrin-B1 | 1.749788152 |
| Tpd52l2 | tumor protein D52-like 2 | 1.733803267 |
| Atg3 | ATG3 autophagy related 3 homolog (S. cerevisiae) | 1.728880039 |
| Gabarapl2 | GABA(A) receptor-associated protein-like 2 | 1.722973077 |
| Sfrp1 | secreted frizzled-related protein 1 | 1.720425458 |
| Ezh2 | enhancer of zeste homolog 2 (Drosophila) | 1.720093237 |
| Rev3l | REV3-like, catalytic subunit of DNA polymerase zeta (yeast) | 1.716888023 |
| Mthfd2 | methylenetetrahydrofolate dehydrogenase (NADP+ dependent) 2, methenyltetrahydrofolate cyclohydrolase | 1.715733759 |
| Notch1 | Notch homolog 1, translocation-associated (Drosophila) | 1.711620975 |
| Thsd1 | thrombospondin, type I, domain containing 1 | 1.711382133 |
| Stx6 | syntaxin 6 | 1.71130295 |
| Nr1d2 | nuclear receptor subfamily 1, group D, member 2 | 1.705371503 |
| Tmem158 | transmembrane protein 158 | 1.704511636 |
| Rfc5 | replication factor C (activator 1) 5, 36.5kDa | 1.693984201 |
| Slc18a3 | solute carrier family 18 (vesicular acetylcholine), member 3 | 1.692519125 |
| Lix1 | Lix1 homolog (chicken) | 1.689005048 |
| Pabpc1 | poly(A) binding protein, cytoplasmic 1 | 1.687377497 |
| Krcc1 | lysine-rich coiled-coil 1 | 1.685856873 |
| Kctd20 | potassium channel tetramerisation domain containing 20 | 1.683268621 |
| Adal | adenosine deaminase-like | 1.678200145 |
| P2ry14 | purinergic receptor P2Y, G-protein coupled, 14 | 1.677385873 |
| Donson | downstream neighbor of SON | 1.675804954 |
| Loh12cr1 | loss of heterozygosity, 12, chromosomal region 1 | 1.672081298 |
| Atp2a3 | ATPase, Ca++ transporting, ubiquitous | 1.669647363 |
| Lysmd2 | LysM, putative peptidoglycan-binding, domain containing 2 | 1.665922545 |
| Ergic2 | ERGIC and golgi 2 | 1.662720757 |
| Zfml | zinc finger protein 638 | 1.661710669 |
| Mier1 | mesoderm induction early response 1 homolog (Xenopus laevis) | 1.66031109 |
| BC028528 | chromosome 1 open reading frame 54 | 1.659077165 |
| Krt73 | keratin 73 | 1.658111066 |
| Hmg20a | high-mobility group 20A | 1.657386273 |
| Npas1 | neuronal PAS domain protein 1 | 1.651016477 |
| Rabep1 | rabaptin, RAB GTPase binding effector protein 1 | 1.650791908 |
| Aldh3a1 | aldehyde dehydrogenase 3 family, memberA1 | 1.646128926 |
| Mbtd1 | mbt domain containing 1 | 1.643738498 |
| Katnb1 | katanin p80 (WD repeat containing) subunit B 1 | 1.626078357 |
| Psmc3ip | PSMC3 interacting protein | 1.624370816 |
| Aldh1l2 | aldehyde dehydrogenase 1 family, member L2 | 1.612107322 |
| Cryba2 | crystallin, beta A2 | 1.611491445 |
| Polg | polymerase (DNA directed), gamma | 1.602552049 |
| Sema5b | sema domain, seven thrombospondin repeats (type 1 and type 1-like), transmembrane domain (TM) and short cytoplasmic domain, (semaphorin) 5B | 1.598266415 |
| Atp5a1 | ATP synthase, H+ transporting, mitochondrial F1 complex, alpha subunit 1, cardiac muscle | 1.597389041 |
| Hps4 | Hermansky-Pudlak syndrome 4 | 1.596398055 |
| Top1 | topoisomerase (DNA) I | 1.582734048 |
| Commd1 | copper metabolism (Murr1) domain containing 1 | 1.576115286 |
| Pfkl | phosphofructokinase, liver | 1.570591462 |
| Epm2aip1 | EPM2A (laforin) interacting protein 1 | 1.553726199 |
| Rpl21 | ribosomal protein L21 | 1.546769126 |
| Peci | peroxisomal D3,D2-enoyl-CoA isomerase | 1.539215578 |
| Smyd5 | SMYD family member 5 | 1.532449218 |
| Bmpr1a | bone morphogenetic protein receptor, type IA | 1.532237331 |
| Ap3s2 | adaptor-related protein complex 3, sigma 2 subunit | 1.522249186 |
| Vip | vasoactive intestinal peptide | 1.520291079 |
| Bcam | basal cell adhesion molecule (Lutheran blood group) | 1.5183169 |
| Bxdc1 | brix domain containing 1 | 1.513855554 |
| Usp14 | ubiquitin specific peptidase 14 (tRNA-guanine transglycosylase) | 1.513825255 |
| Tmed8 | transmembrane emp24 protein transport domain containing 8 | 1.510782748 |
| Phf13 | PHD finger protein 13 | 1.508838962 |
| Rhod | ras homolog gene family, member D | -1.500475344 |
| Smpd2 | sphingomyelin phosphodiesterase 2, neutral membrane (neutral sphingomyelinase) | -1.50310345 |
| Krt12 | keratin 12 | -1.505562206 |
| Dtd1 | D-tyrosyl-tRNA deacylase 1 homolog (S. cerevisiae) | -1.506125905 |
| Mrpl20 | mitochondrial ribosomal protein L20 | -1.510232694 |
| Med28 | mediator complex subunit 28 | -1.527710421 |
| Relt | RELT tumor necrosis factor receptor | -1.531429511 |
| Terf2ip | telomeric repeat binding factor 2, interacting protein | -1.534634708 |
| Fbxl4 | F-box and leucine-rich repeat protein 4 | -1.545908821 |
| Wdr59 | WD repeat domain 59 | -1.547215729 |
| Nde1 | nudE nuclear distribution gene E homolog 1 (A. nidulans) | -1.548428208 |
| Irf9 | interferon regulatory factor 9 | -1.551871787 |
| Mlstd2 | fatty acyl CoA reductase 1 | -1.561548638 |
| Sema7a | semaphorin 7A, GPI membrane anchor (John Milton Hagen blood group) | -1.562573549 |
| Arrdc3 | arrestin domain containing 3 | -1.56336768 |
| Rhbdl3 | rhomboid, veinlet-like 3 (Drosophila) | -1.563893907 |
| Psmg2 | proteasome (prosome, macropain) assembly chaperone 2 | -1.564900538 |
| Etfa | electron-transfer-flavoprotein, alpha polypeptide | -1.565268191 |
| Trip12 | thyroid hormone receptor interactor 12 | -1.568400175 |
| Pgm2 | phosphoglucomutase 2 | -1.569196474 |
| Dnajb9 | DnaJ (Hsp40) homolog, subfamily B, member 9 | -1.56923715 |
| Xab1 | GPN-loop GTPase 1 | -1.574233586 |
| Dgkh | diacylglycerol kinase, eta | -1.574856451 |
| Asah3l | alkaline ceramidase 2 | -1.580734302 |
| Adcy9 | adenylate cyclase 9 | -1.581544274 |
| Gpr27 | G protein-coupled receptor 27 | -1.587731188 |
| Slc35f4 | solute carrier family 35, member F4 | -1.597132901 |
| Tbl3 | transducin (beta)-like 3 | -1.606845879 |
| Txndc1 | thioredoxin-related transmembrane protein 1 | -1.60939469 |
| Clstn3 | calsyntenin 3 | -1.61122243 |
| Nab1 | NGFI-A binding protein 1 (EGR1 binding protein 1) | -1.615454891 |
| Ppp2r5d | protein phosphatase 2, regulatory subunit B', delta isoform | -1.627143 |
| Rundc3b | RUN domain containing 3B | -1.630351942 |
| BC013529 | chromosome 6 open reading frame 72 | -1.634494875 |
| Polr2g | polymerase (RNA) II (DNA directed) polypeptide G | -1.639519369 |
| Pcdhgb6 | protocadherin gamma subfamily B, 6 | -1.641460797 |
| Pygm | phosphorylase, glycogen, muscle | -1.644690802 |
| Smcr7l | Smith-Magenis syndrome chromosome region, candidate 7-like | -1.6491087 |
| Gpr135 | G protein-coupled receptor 135 | -1.649452535 |
| Dynlt3 | dynein, light chain, Tctex-type 3 | -1.650331718 |
| Fcf1 | FCF1 small subunit (SSU) processome component homolog (S. cerevisiae) | -1.653434673 |
| Eif3k | eukaryotic translation initiation factor 3, subunit K | -1.665082245 |
| BC026585 | cDNA sequence BC026585 | -1.676001201 |
| Terf2 | telomeric repeat binding factor 2 | -1.682547367 |
| Rhoc | ras homolog gene family, member C | -1.684058559 |
| Xpo7 | exportin 7 | -1.684488172 |
| Otud5 | OTU domain containing 5 | -1.688990508 |
| Slc4a8 | solute carrier family 4, sodium bicarbonate cotransporter, member 8 | -1.689349117 |
| Psmd11 | proteasome (prosome, macropain) 26S subunit, non-ATPase, 11 | -1.70056206 |
| Chpt1 | choline phosphotransferase 1 | -1.705190364 |
| Exoc7 | exocyst complex component 7 | -1.707921112 |
| Tyro3 | TYRO3 protein tyrosine kinase | -1.713604146 |
| Amhr2 | anti-Mullerian hormone receptor, type II | -1.721167214 |
| Shc3 | SHC (Src homology 2 domain containing) transforming protein 3 | -1.722233757 |
| Umps | uridine monophosphate synthetase | -1.743700565 |
| Rad52 | RAD52 homolog (S. cerevisiae) | -1.746821469 |
| Slc10a4 | solute carrier family 10 (sodium/bile acid cotransporter family), member 4 | -1.751540627 |
| Hhex | hematopoietically expressed homeobox | -1.752651933 |
| Nck1 | NCK adaptor protein 1 | -1.753463485 |
| Nid1 | nidogen 1 | -1.753675667 |
| Mllt1 | myeloid/lymphoid or mixed-lineage leukemia (trithorax homolog, Drosophila); translocated to, 1 | -1.754113258 |
| Nup88 | nucleoporin 88kDa | -1.756392568 |
| Oxr1 | oxidation resistance 1 | -1.762987034 |
| Ryr3 | ryanodine receptor 3 | -1.764863436 |
| Nf1 | neurofibromin 1 | -1.768050473 |
| Pomt2 | protein-O-mannosyltransferase 2 | -1.771510654 |
| BC011248 | chromosome 6 open reading frame 153 | -1.774494804 |
| Hcrtr1 | hypocretin (orexin) receptor 1 | -1.779324108 |
| Scarb2 | scavenger receptor class B, member 2 | -1.782729081 |
| Stra13 | stimulated by retinoic acid 13 homolog (mouse) | -1.784574721 |
| Pcgf3 | polycomb group ring finger 3 | -1.786711356 |
| Wipi1 | WD repeat domain, phosphoinositide interacting 1 | -1.789150759 |
| Stard5 | StAR-related lipid transfer (START) domain containing 5 | -1.792153814 |
| Wwox | WW domain containing oxidoreductase | -1.792472743 |
| Kcnj16 | potassium inwardly-rectifying channel, subfamily J, member 16 | -1.796226539 |
| Snx19 | sorting nexin 19 | -1.799958746 |
| Mkrn1 | makorin ring finger protein 1 | -1.808351141 |
| Surf4 | surfeit 4 | -1.813342747 |
| Txndc11 | thioredoxin domain containing 11 | -1.814142242 |
| Stoml1 | stomatin (EPB72)-like 1 | -1.827073087 |
| Nt5c2 | 5'-nucleotidase, cytosolic II | -1.841229202 |
| Rxrg | retinoid X receptor, gamma | -1.853278509 |
| Pofut1 | protein O-fucosyltransferase 1 | -1.856970408 |
| Ptrh1 | peptidyl-tRNA hydrolase 1 homolog (S. cerevisiae) | -1.87548886 |
| Rfx2 | regulatory factor X, 2 (influences HLA class II expression) | -1.878438032 |
| Zfyve27 | zinc finger, FYVE domain containing 27 | -1.894094343 |
| Arid3b | AT rich interactive domain 3B (BRIGHT-like) | -1.894107508 |
| Rasal1 | RAS protein activator like 1 (GAP1 like) | -1.909677198 |
| Zic4 | Zic family member 4 | -1.911417382 |
| Gmppa | GDP-mannose pyrophosphorylase A | -1.921937112 |
| Scyl1 | SCY1-like 1 (S. cerevisiae) | -1.922323041 |
| Ubqln1 | ubiquilin 1 | -1.923345987 |
| Agpat1 | 1-acylglycerol-3-phosphate O-acyltransferase 1 (lysophosphatidic acid acyltransferase, alpha) | -1.927018035 |
| Nus1 | nuclear undecaprenyl pyrophosphate synthase 1 homolog (S. cerevisiae) | -1.92908152 |
| Map3k14 | mitogen-activated protein kinase kinase kinase 14 | -1.941059722 |
| Nt5c | 5', 3'-nucleotidase, cytosolic | -1.947667474 |
| Khdrbs2 | KH domain containing, RNA binding, signal transduction associated 2 | -1.953776071 |
| Rbbp6 | retinoblastoma binding protein 6 | -1.956829332 |
| Ephb6 | EPH receptor B6 | -1.975147278 |
| Clic6 | chloride intracellular channel 6 | -1.983620409 |
| Rnuxa | phosphorylated adaptor for RNA export | -1.988466271 |
| Nxf1 | nuclear RNA export factor 1 | -1.993455937 |
| Mcph1 | microcephalin 1 | -1.999801993 |
| Cecr6 | cat eye syndrome chromosome region, candidate 6 | -2.003190476 |
| Rnf34 | ring finger protein 34 | -2.016248356 |
| Dak | dihydroxyacetone kinase 2 homolog (S. cerevisiae) | -2.026837697 |
| Ube1c | ubiquitin-like modifier activating enzyme 3 | -2.027675198 |
| Dgat1 | diacylglycerol O-acyltransferase homolog 1 (mouse) | -2.027807795 |
| Znrf2 | zinc and ring finger 2 | -2.032101518 |
| Mta1 | metastasis associated 1 | -2.034796517 |
| Tdp1 | tyrosyl-DNA phosphodiesterase 1 | -2.036002284 |
| Stk24 | serine/threonine kinase 24 (STE20 homolog, yeast) | -2.047903928 |
| B4galt7 | xylosylprotein beta 1,4-galactosyltransferase, polypeptide 7 (galactosyltransferase I) | -2.055018041 |
| Per3 | period homolog 3 (Drosophila) | -2.058891507 |
| P2ry6 | pyrimidinergic receptor P2Y, G-protein coupled, 6 | -2.065422119 |
| Chrna3 | cholinergic receptor, nicotinic, alpha 3 | -2.067428653 |
| Clgn | calmegin | -2.069320535 |
| Nfx1 | nuclear transcription factor, X-box binding 1 | -2.078041434 |
| Nrg3 | neuregulin 3 | -2.079611156 |
| Sec15l1 | exocyst complex component 6 | -2.094386871 |
| Csad | cysteine sulfinic acid decarboxylase | -2.101428313 |
| Ebag9 | estrogen receptor binding site associated, antigen, 9 | -2.112873808 |
| Sh3gl3 | SH3-domain GRB2-like 3 | -2.114223598 |
| Scube3 | signal peptide, CUB domain, EGF-like 3 | -2.114865217 |
| Arhgef1 | Rho guanine nucleotide exchange factor (GEF) 1 | -2.119278252 |
| Emid2 | EMI domain containing 2 | -2.144869467 |
| Cyfip1 | cytoplasmic FMR1 interacting protein 1 | -2.15769358 |
| Kcnn1 | potassium intermediate/small conductance calcium-activated channel, subfamily N, member 1 | -2.158023634 |
| Epas1 | endothelial PAS domain protein 1 | -2.176252132 |
| Kcnab3 | potassium voltage-gated channel, shaker-related subfamily, beta member 3 | -2.184693709 |
| Cdc16 | cell division cycle 16 homolog (S. cerevisiae) | -2.189717069 |
| Cybasc3 | cytochrome b, ascorbate dependent 3 | -2.198891466 |
| Ahctf1 | AT hook containing transcription factor 1 | -2.212940255 |
| Snx13 | sorting nexin 13 | -2.22227602 |
| Ankrd46 | ankyrin repeat domain 46 | -2.228128283 |
| Tmem19 | transmembrane protein 19 | -2.255679904 |
| Ascc3l1 | small nuclear ribonucleoprotein 200kDa (U5) | -2.262320035 |
| Tmem179b | transmembrane protein 179B | -2.278170829 |
| Adam22 | ADAM metallopeptidase domain 22 | -2.294461292 |
| Plscr1 | phospholipid scramblase 1 | -2.296726615 |
| Klhl8 | kelch-like 8 (Drosophila) | -2.328907154 |
| Pelp1 | proline, glutamate and leucine rich protein 1 | -2.340763354 |
| Rasl11a | RAS-like, family 11, member A | -2.342696477 |
| Pld5 | phospholipase D family, member 5 | -2.351563091 |
| Mal | mal, T-cell differentiation protein | -2.368853568 |
| Ela1 | chymotrypsin-like elastase family, member 1 | -2.372220565 |
| Adprhl2 | ADP-ribosylhydrolase like 2 | -2.374191175 |
| Nrg1 | neuregulin 1 | -2.376121499 |
| Suhw2 | zinc finger protein 280B | -2.37625852 |
| Cep97 | centrosomal protein 97kDa | -2.390488428 |
| C8g | complement component 8, gamma polypeptide | -2.394206427 |
| Cp | ceruloplasmin (ferroxidase) | -2.408973587 |
| Ak3l1 | adenylate kinase 3-like 1 | -2.40943452 |
| Npr2 | natriuretic peptide receptor B/guanylate cyclase B (atrionatriuretic peptide receptor B) | -2.414999539 |
| Stk36 | serine/threonine kinase 36, fused homolog (Drosophila) | -2.420559417 |
| Lmo3 | LIM domain only 3 (rhombotin-like 2) | -2.421260537 |
| Myl6b | myosin, light chain 6B, alkali, smooth muscle and non-muscle | -2.422314917 |
| Ndufb5 | NADH dehydrogenase (ubiquinone) 1 beta subcomplex, 5, 16kDa | -2.429075668 |
| Plekhg2 | pleckstrin homology domain containing, family G (with RhoGef domain) member 2 | -2.442460645 |
| Lrrc33 | leucine rich repeat containing 33 | -2.482447074 |
| Slitrk3 | SLIT and NTRK-like family, member 3 | -2.508783781 |
| Capza1 | capping protein (actin filament) muscle Z-line, alpha 1 | -2.521738518 |
| BC050811 | coiled-coil domain containing 144B | -2.52246268 |
| Bmi1 | BMI1 polycomb ring finger oncogene | -2.536886062 |
| Tssc1 | tumor suppressing subtransferable candidate 1 | -2.541719222 |
| Cchcr1 | coiled-coil alpha-helical rod protein 1 | -2.548430065 |
| Cd274 | CD274 molecule | -2.566028763 |
| Cul1 | cullin 1 | -2.576801696 |
| Mustn1 | musculoskeletal, embryonic nuclear protein 1 | -2.582394643 |
| Slc38a10 | solute carrier family 38, member 10 | -2.594762774 |
| Zfp36 | zinc finger protein 36, C3H type, homolog (mouse) | -2.614954997 |
| BC038925 | metallo-beta-lactamase domain containing 1 | -2.622949865 |
| Tjp2 | tight junction protein 2 (zona occludens 2) | -2.625531071 |
| Nkd2 | naked cuticle homolog 2 (Drosophila) | -2.632928867 |
| Tmtc4 | transmembrane and tetratricopeptide repeat containing 4 | -2.640083956 |
| Mrp63 | mitochondrial ribosomal protein 63 | -2.651820529 |
| Picalm | phosphatidylinositol binding clathrin assembly protein | -2.65443864 |
| Asl | argininosuccinate lyase | -2.668203423 |
| Prima1 | proline rich membrane anchor 1 | -2.67405321 |
| Mars | methionyl-tRNA synthetase | -2.696071887 |
| Id3 | inhibitor of DNA binding 3, dominant negative helix-loop-helix protein | -2.716250503 |
| Asb2 | ankyrin repeat and SOCS box-containing 2 | -2.722126894 |
| Col7a1 | collagen, type VII, alpha 1 | -2.735926125 |
| Htr1d | 5-hydroxytryptamine (serotonin) receptor 1D | -2.747277837 |
| Cdca7 | cell division cycle associated 7 | -2.754988148 |
| Coro2a | coronin, actin binding protein, 2A | -2.770074678 |
| Ppfibp2 | PTPRF interacting protein, binding protein 2 (liprin beta 2) | -2.772976734 |
| Fchsd1 | FCH and double SH3 domains 1 | -2.775573395 |
| Arf5 | ADP-ribosylation factor 5 | -2.778182086 |
| Pyroxd1 | pyridine nucleotide-disulphide oxidoreductase domain 1 | -2.815032216 |
| Elfn1 | extracellular leucine-rich repeat and fibronectin type III domain containing 1 | -2.815647558 |
| Nrip2 | nuclear receptor interacting protein 2 | -2.81919243 |
| Decr2 | 2,4-dienoyl CoA reductase 2, peroxisomal | -2.831842878 |
| Uevld | UEV and lactate/malate dehyrogenase domains | -2.833317476 |
| Sorcs2 | sortilin-related VPS10 domain containing receptor 2 | -2.835462832 |
| Rnf168 | ring finger protein 168 | -2.836451298 |
| Sh3glb1 | SH3-domain GRB2-like endophilin B1 | -2.861649632 |
| Spry4 | sprouty homolog 4 (Drosophila) | -2.864726574 |
| Sars2 | seryl-tRNA synthetase 2, mitochondrial | -2.872098892 |
| Pthr1 | parathyroid hormone 1 receptor | -2.890430608 |
| Arpc5l | actin related protein 2/3 complex, subunit 5-like | -2.914385142 |
| Cmtm8 | CKLF-like MARVEL transmembrane domain containing 8 | -2.934250838 |
| Kctd12 | potassium channel tetramerisation domain containing 12 | -2.938320882 |
| Bmp7 | bone morphogenetic protein 7 | -2.94879032 |
| Hn1 | hematological and neurological expressed 1 | -2.961720755 |
| Hadhb | hydroxyacyl-Coenzyme A dehydrogenase/3-ketoacyl-Coenzyme A thiolase/enoyl-Coenzyme A hydratase (trifunctional protein), beta subunit | -2.972992219 |
| Gtf3c5 | general transcription factor IIIC, polypeptide 5, 63kDa | -3.009902833 |
| Dyrk3 | dual-specificity tyrosine-(Y)-phosphorylation regulated kinase 3 | -3.036335338 |
| Wdsof1 | WD repeats and SOF1 domain containing | -3.055651097 |
| Tmem32 | membrane magnesium transporter 1 | -3.081590439 |
| Pdcd6ip | programmed cell death 6 interacting protein | -3.106849358 |
| Tmtc2 | transmembrane and tetratricopeptide repeat containing 2 | -3.129387026 |
| Polb | polymerase (DNA directed), beta | -3.133169284 |
| C78339 | family with sequence similarity 8, member A1 | -3.137866108 |
| Rin3 | Ras and Rab interactor 3 | -3.191730009 |
| Hnrpf | heterogeneous nuclear ribonucleoprotein F | -3.276214079 |
| Dirc2 | disrupted in renal carcinoma 2 | -3.297152946 |
| Slc30a7 | solute carrier family 30 (zinc transporter), member 7 | -3.298177764 |
| Jdp2 | Jun dimerization protein 2 | -3.317423922 |
| Chst10 | carbohydrate sulfotransferase 10 | -3.319475345 |
| Eps15 | epidermal growth factor receptor pathway substrate 15 | -3.364720948 |
| Arrdc2 | arrestin domain containing 2 | -3.374534901 |
| Dusp19 | dual specificity phosphatase 19 | -3.380867016 |
| Zic1 | Zic family member 1 (odd-paired homolog, Drosophila) | -3.393506756 |
| Hspe1 | heat shock 10kDa protein 1 (chaperonin 10) | -3.552422303 |
| Ung | uracil-DNA glycosylase | -3.566024389 |
| Dicer1 | dicer 1, ribonuclease type III | -3.637559633 |
| Hdhd3 | haloacid dehalogenase-like hydrolase domain containing 3 | -3.691116592 |
| Pcolce | procollagen C-endopeptidase enhancer | -3.704786281 |
| Rnf216 | ring finger protein 216 | -3.707997239 |
| Spsb2 | splA/ryanodine receptor domain and SOCS box containing 2 | -3.732871404 |
| Rabep2 | rabaptin, RAB GTPase binding effector protein 2 | -3.741978469 |
| Eif2c4 | eukaryotic translation initiation factor 2C, 4 | -3.779367935 |
| Ube2d3 | ubiquitin-conjugating enzyme E2D 3 (UBC4/5 homolog, yeast) | -3.849429023 |
| Igfbp6 | insulin-like growth factor binding protein 6 | -3.849916074 |
| Rbed1 | ELMO/CED-12 domain containing 3 | -3.864483126 |
| Ppp1r12c | protein phosphatase 1, regulatory (inhibitor) subunit 12C | -3.90416922 |
| Ttc27 | tetratricopeptide repeat domain 27 | -3.926282621 |
| E2f1 | E2F transcription factor 1 | -3.946458488 |
| Mcat | malonyl CoA:ACP acyltransferase (mitochondrial) | -3.967589003 |
| Accs | 1-aminocyclopropane-1-carboxylate synthase homolog (Arabidopsis)(non-functional) | -3.987729128 |
| Nedd1 | neural precursor cell expressed, developmentally down-regulated 1 | -3.993849191 |
| Rnf26 | ring finger protein 26 | -3.994969296 |
| Snf1lk2 | salt-inducible kinase 2 | -4.011746643 |
| Lrrk1 | leucine-rich repeat kinase 1 | -4.079854457 |
| Naga | N-acetylgalactosaminidase, alpha- | -4.101143492 |
| Mtss1 | metastasis suppressor 1 | -4.124299216 |
| Ptplad2 | protein tyrosine phosphatase-like A domain containing 2 | -4.213731213 |
| Kcnk4 | potassium channel, subfamily K, member 4 | -4.25019739 |
| Ftsj1 | FtsJ homolog 1 (E. coli) | -4.368325911 |
| Adcyap1r1 | adenylate cyclase activating polypeptide 1 (pituitary) receptor type I | -4.394777852 |
| Ubiad1 | UbiA prenyltransferase domain containing 1 | -4.431840377 |
| Hfe | hemochromatosis | -4.443226284 |
| C1qtnf2 | C1q and tumor necrosis factor related protein 2 | -4.529355154 |
| Apoa2 | apolipoprotein A-II | -4.529841728 |
| Lama1 | laminin, alpha 1 | -4.571889204 |
| AI467606 | chromosome 16 open reading frame 54 | -4.619406312 |
| Tead2 | TEA domain family member 2 | -4.630393396 |
| Slc26a6 | solute carrier family 26, member 6 | -4.755564608 |
| Abhd14a | abhydrolase domain containing 14A | -4.843255125 |
| Slc22a7 | solute carrier family 22 (organic anion transporter), member 7 | -4.844656767 |
| Tmem138 | transmembrane protein 138 | -5.07142544 |
| Pls3 | plastin 3 (T isoform) | -5.3844713 |
| Hif1a | hypoxia inducible factor 1, alpha subunit (basic helix-loop-helix transcription factor) | -5.664330278 |
| Tmem176b | transmembrane protein 176B | -5.734727972 |
| Ccdc90b | coiled-coil domain containing 90B | -6.007179745 |
| Cln6 | ceroid-lipofuscinosis, neuronal 6, late infantile, variant | -6.110227884 |
| Pltp | phospholipid transfer protein | -6.330772118 |
| Igf1 | insulin-like growth factor 1 (somatomedin C) | -6.786947576 |

**Table S5.** Hypothalamic gene transcripts differentially expressed in running (run) -*ad libitum* *db/db* mice compared to sedentary (sed) -*ad libitum* *db/db* mice (n=3 animals per group). Statistical significance was considered at p≤0.05.

| **Gene Symbol** | **Description** | **z-ratio (run vs. sed)** |
| --- | --- | --- |
| Naga | N-acetylgalactosaminidase, alpha- | 7.096186558 |
| Hace1 | HECT domain and ankyrin repeat containing, E3 ubiquitin protein ligase 1 | 4.650617345 |
| Heatr5a | HEAT repeat containing 5A | 4.199688599 |
| Slc22a7 | solute carrier family 22 (organic anion transporter), member 7 | 4.128936805 |
| Suhw2 | zinc finger protein 280B | 3.592537092 |
| Nqo1 | NAD(P)H dehydrogenase, quinone 1 | 3.50635649 |
| Acp6 | acid phosphatase 6, lysophosphatidic | 3.49293846 |
| Wdr59 | WD repeat domain 59 | 3.474229963 |
| Rasl10a | RAS-like, family 10, member A | 3.38051374 |
| Ccdc85a | coiled-coil domain containing 85A | 3.37331735 |
| Golga4 | golgi autoantigen, golgin subfamily a, 4 | 3.275141569 |
| Hcrtr1 | hypocretin (orexin) receptor 1 | 3.265437178 |
| Ptplad2 | protein tyrosine phosphatase-like A domain containing 2 | 3.225630507 |
| Syt13 | synaptotagmin XIII | 3.225277114 |
| Rbed1 | ELMO/CED-12 domain containing 3 | 3.222753517 |
| Kcnu1 | potassium channel, subfamily U, member 1 | 3.214134926 |
| N6amt2 | N-6 adenine-specific DNA methyltransferase 2 (putative) | 3.167311112 |
| Fgfrl1 | fibroblast growth factor receptor-like 1 | 3.093850682 |
| Ypel5 | yippee-like 5 (Drosophila) | 3.039568793 |
| Arpc5l | actin related protein 2/3 complex, subunit 5-like | 2.949984302 |
| Capg | capping protein (actin filament), gelsolin-like | 2.914000756 |
| Arf5 | ADP-ribosylation factor 5 | 2.870354135 |
| Ccdc90b | coiled-coil domain containing 90B | 2.862507476 |
| Tbc1d23 | TBC1 domain family, member 23 | 2.81890618 |
| Tes | testis derived transcript (3 LIM domains) | 2.793170862 |
| Apoa2 | apolipoprotein A-II | 2.784717012 |
| Dusp19 | dual specificity phosphatase 19 | 2.780962549 |
| Mta1 | metastasis associated 1 | 2.693959169 |
| Gmip | GEM interacting protein | 2.688143576 |
| Msc | musculin (activated B-cell factor-1) | 2.667393905 |
| Ung | uracil-DNA glycosylase | 2.646640431 |
| Ascc3l1 | small nuclear ribonucleoprotein 200kDa (U5) | 2.606981138 |
| Acyp2 | acylphosphatase 2, muscle type | 2.537910703 |
| Gabrb2 | gamma-aminobutyric acid (GABA) A receptor, beta 2 | 2.48681982 |
| Slc12a8 | solute carrier family 12 (potassium/chloride transporters), member 8 | 2.481326461 |
| Slc30a7 | solute carrier family 30 (zinc transporter), member 7 | 2.476639136 |
| Ppfibp2 | PTPRF interacting protein, binding protein 2 (liprin beta 2) | 2.469248288 |
| Thap4 | THAP domain containing 4 | 2.459284302 |
| Scrn1 | secernin 1 | 2.449420326 |
| Bcorl1 | BCL6 co-repressor-like 1 | 2.427582896 |
| Ly86 | lymphocyte antigen 86 | 2.425126703 |
| Oplah | 5-oxoprolinase (ATP-hydrolysing) | 2.417748176 |
| Nle1 | notchless homolog 1 (Drosophila) | 2.368518515 |
| Rprml | reprimo-like | 2.368047528 |
| Commd8 | COMM domain containing 8 | 2.363452707 |
| Lrrc40 | leucine rich repeat containing 40 | 2.359935302 |
| Jag2 | jagged 2 | 2.350050017 |
| Fndc3b | fibronectin type III domain containing 3B | 2.345272409 |
| Rabep2 | rabaptin, RAB GTPase binding effector protein 2 | 2.342314821 |
| Pld2 | phospholipase D2 | 2.340492793 |
| Tmtc2 | transmembrane and tetratricopeptide repeat containing 2 | 2.338388417 |
| Rpusd1 | RNA pseudouridylate synthase domain containing 1 | 2.326169842 |
| Cdh22 | cadherin-like 22 | 2.321497249 |
| Aph1a | anterior pharynx defective 1 homolog A (C. elegans) | 2.289589798 |
| Opa1 | optic atrophy 1 (autosomal dominant) | 2.288868325 |
| Trps1 | trichorhinophalangeal syndrome I | 2.270309301 |
| Lst1 | leukocyte specific transcript 1 | 2.262133085 |
| Hpcal1 | hippocalcin-like 1 | 2.260204536 |
| Sars2 | seryl-tRNA synthetase 2, mitochondrial | 2.254576751 |
| Irx5 | iroquois homeobox 5 | 2.243963254 |
| Hist1h2ad | histone cluster 1, H2ad | 2.243194411 |
| Gatad2b | GATA zinc finger domain containing 2B | 2.22015238 |
| Hspa12b | heat shock 70kD protein 12B | 2.218663445 |
| Spag5 | sperm associated antigen 5 | 2.21059324 |
| Trpc7 | transient receptor potential cation channel, subfamily C, member 7 | 2.186741601 |
| Hps6 | Hermansky-Pudlak syndrome 6 | 2.184512071 |
| Ddit4l | DNA-damage-inducible transcript 4-like | 2.154580112 |
| Hadhb | hydroxyacyl-Coenzyme A dehydrogenase/3-ketoacyl-Coenzyme A thiolase/enoyl-Coenzyme A hydratase (trifunctional protein), beta subunit | 2.133397094 |
| Fbxo30 | F-box protein 30 | 2.123952449 |
| Smcr7l | Smith-Magenis syndrome chromosome region, candidate 7-like | 2.118593464 |
| Mterfd1 | MTERF domain containing 1 | 2.114003164 |
| Hs3st3a1 | heparan sulfate (glucosamine) 3-O-sulfotransferase 3A1 | 2.104951423 |
| Cdc37 | cell division cycle 37 homolog (S. cerevisiae) | 2.099837628 |
| Slc22a6 | solute carrier family 22 (organic anion transporter), member 6 | 2.096702824 |
| Papd4 | PAP associated domain containing 4 | 2.096091726 |
| Ciz1 | CDKN1A interacting zinc finger protein 1 | 2.092406586 |
| Islr2 | immunoglobulin superfamily containing leucine-rich repeat 2 | 2.076359126 |
| Nhej1 | nonhomologous end-joining factor 1 | 2.07322352 |
| Tyro3 | TYRO3 protein tyrosine kinase | 2.055216785 |
| Mboat1 | membrane bound O-acyltransferase domain containing 1 | 2.053115512 |
| Ebag9 | estrogen receptor binding site associated, antigen, 9 | 2.037401241 |
| Tnfrsf19 | tumor necrosis factor receptor superfamily, member 19 | 2.037074735 |
| Shc3 | SHC (Src homology 2 domain containing) transforming protein 3 | 2.034061552 |
| Trem2 | triggering receptor expressed on myeloid cells 2 | 2.033497799 |
| Zfml | zinc finger protein 638 | 2.032808567 |
| Leprel2 | leprecan-like 2 | 2.026703179 |
| Psmd14 | proteasome (prosome, macropain) 26S subunit, non-ATPase, 14 | 2.014135512 |
| Arhgef1 | Rho guanine nucleotide exchange factor (GEF) 1 | 2.008670443 |
| Stk40 | serine/threonine kinase 40 | 1.993998413 |
| Gfra4 | GDNF family receptor alpha 4 | 1.988332916 |
| Kcna1 | potassium voltage-gated channel, shaker-related subfamily, member 1 (episodic ataxia with myokymia) | 1.978259389 |
| Mycbp | c-myc binding protein | 1.961728195 |
| Tmem179b | transmembrane protein 179B | 1.959264486 |
| Nkd2 | naked cuticle homolog 2 (Drosophila) | 1.958753621 |
| Coro6 | coronin 6 | 1.958055423 |
| Map3k1 | mitogen-activated protein kinase kinase kinase 1 | 1.946042388 |
| Gtf2h5 | general transcription factor IIH, polypeptide 5 | 1.943441624 |
| Igsf9 | immunoglobulin superfamily, member 9 | 1.932532967 |
| Megf9 | multiple EGF-like-domains 9 | 1.917861688 |
| Xab1 | GPN-loop GTPase 1 | 1.9134881 |
| Pcyt1a | phosphate cytidylyltransferase 1, choline, alpha | 1.905931268 |
| Ftsj1 | FtsJ homolog 1 (E. coli) | 1.903970164 |
| Sec11c | SEC11 homolog C (S. cerevisiae) | 1.903162672 |
| Atf5 | activating transcription factor 5 | 1.886667919 |
| Cchcr1 | coiled-coil alpha-helical rod protein 1 | 1.886332819 |
| Acrbp | acrosin binding protein | 1.882072585 |
| Rad1 | RAD1 homolog (S. pombe) | 1.879154964 |
| Dapk3 | death-associated protein kinase 3 | 1.874021722 |
| Lhx5 | LIM homeobox 5 | 1.870978591 |
| Etv1 | ets variant 1 | 1.85809827 |
| Tssc4 | tumor suppressing subtransferable candidate 4 | 1.857142221 |
| Efcab2 | EF-hand calcium binding domain 2 | 1.856998416 |
| Samd5 | sterile alpha motif domain containing 5 | 1.846118192 |
| Wdr78 | WD repeat domain 78 | 1.845825111 |
| Zfyve27 | zinc finger, FYVE domain containing 27 | 1.845117751 |
| Samd9l | sterile alpha motif domain containing 9-like | 1.837032123 |
| Insc | inscuteable homolog (Drosophila) | 1.833025552 |
| Nanos2 | nanos homolog 2 (Drosophila) | 1.81656926 |
| Cdca7 | cell division cycle associated 7 | 1.814046477 |
| Txndc1 | thioredoxin-related transmembrane protein 1 | 1.812744393 |
| Ears2 | glutamyl-tRNA synthetase 2, mitochondrial (putative) | 1.79794586 |
| Gbp2 | guanylate binding protein 2, interferon-inducible | 1.797944157 |
| Asgr1 | asialoglycoprotein receptor 1 | 1.788666 |
| Gosr1 | golgi SNAP receptor complex member 1 | 1.787419389 |
| Csad | cysteine sulfinic acid decarboxylase | 1.78682529 |
| Supv3l1 | suppressor of var1, 3-like 1 (S. cerevisiae) | 1.784219445 |
| Pygo1 | pygopus homolog 1 (Drosophila) | 1.782308244 |
| Lingo4 | leucine rich repeat and Ig domain containing 4 | 1.781159022 |
| Stk36 | serine/threonine kinase 36, fused homolog (Drosophila) | 1.780777474 |
| St6galnac2 | ST6 (alpha-N-acetyl-neuraminyl-2,3-beta-galactosyl-1,3)-N-acetylgalactosaminide alpha-2,6-sialyltransferase 2 | 1.776914633 |
| Hspb8 | heat shock 22kDa protein 8 | 1.772049714 |
| Ezh2 | enhancer of zeste homolog 2 (Drosophila) | 1.76053435 |
| Nfkbib | nuclear factor of kappa light polypeptide gene enhancer in B-cells inhibitor, beta | 1.759833027 |
| Sat2 | spermidine/spermine N1-acetyltransferase family member 2 | 1.75848648 |
| Nup88 | nucleoporin 88kDa | 1.752519279 |
| Col1a1 | collagen, type I, alpha 1 | 1.752010245 |
| Yipf1 | Yip1 domain family, member 1 | 1.748621432 |
| Ndrg4 | NDRG family member 4 | 1.748021582 |
| Zbtb22 | zinc finger and BTB domain containing 22 | 1.740591145 |
| Rnf26 | ring finger protein 26 | 1.737087503 |
| Rhbdl3 | rhomboid, veinlet-like 3 (Drosophila) | 1.733588665 |
| Tmem150 | transmembrane protein 150 | 1.73306214 |
| Crtc3 | CREB regulated transcription coactivator 3 | 1.723924426 |
| Tcirg1 | T-cell, immune regulator 1, ATPase, H+ transporting, lysosomal V0 subunit A3 | 1.723810263 |
| Ptges2 | prostaglandin E synthase 2 | 1.721852856 |
| Map3k6 | mitogen-activated protein kinase kinase kinase 6 | 1.720371867 |
| Dph3 | DPH3, KTI11 homolog (S. cerevisiae) | 1.71671459 |
| Dgkh | diacylglycerol kinase, eta | 1.7139306 |
| Zbtb38 | zinc finger and BTB domain containing 38 | 1.710149296 |
| Npy1r | neuropeptide Y receptor Y1 | 1.708010742 |
| Snf1lk2 | salt-inducible kinase 2 | 1.700230472 |
| Loxl1 | lysyl oxidase-like 1 | 1.696952601 |
| Tirap | toll-interleukin 1 receptor (TIR) domain containing adaptor protein | 1.693741716 |
| Taf3 | TAF3 RNA polymerase II, TATA box binding protein (TBP)-associated factor, 140kDa | 1.69156819 |
| Map1lc3b | microtubule-associated protein 1 light chain 3 beta | 1.687325241 |
| Pcdhb7 | protocadherin beta 7 | 1.670824919 |
| Tmem158 | transmembrane protein 158 | 1.667425955 |
| Apoa1 | apolipoprotein A-I | 1.665905654 |
| Col23a1 | collagen, type XXIII, alpha 1 | 1.662619015 |
| Hdhd3 | haloacid dehalogenase-like hydrolase domain containing 3 | 1.656134318 |
| Asf1a | ASF1 anti-silencing function 1 homolog A (S. cerevisiae) | 1.651507918 |
| Stk24 | serine/threonine kinase 24 (STE20 homolog, yeast) | 1.649184283 |
| Il6st | interleukin 6 signal transducer (gp130, oncostatin M receptor) | 1.631648577 |
| Golim4 | golgi integral membrane protein 4 | 1.625546397 |
| Brp44 | brain protein 44 | 1.617659446 |
| Ppapdc1a | phosphatidic acid phosphatase type 2 domain containing 1A | 1.61658123 |
| Nfkbil2 | nuclear factor of kappa light polypeptide gene enhancer in B-cells inhibitor-like 2 | 1.614487628 |
| Xdh | xanthine dehydrogenase | 1.610843815 |
| Pak4 | p21 protein (Cdc42/Rac)-activated kinase 4 | 1.608054849 |
| Itgb3bp | integrin beta 3 binding protein (beta3-endonexin) | 1.6078013 |
| Nus1 | nuclear undecaprenyl pyrophosphate synthase 1 homolog (S. cerevisiae) | 1.60749823 |
| Ppm1f | protein phosphatase 1F (PP2C domain containing) | 1.606394956 |
| Zan | zonadhesin | 1.606283819 |
| Abcd2 | ATP-binding cassette, sub-family D (ALD), member 2 | 1.603212828 |
| Cacnb4 | calcium channel, voltage-dependent, beta 4 subunit | 1.597716592 |
| Nr1d2 | nuclear receptor subfamily 1, group D, member 2 | 1.596156495 |
| Gpr135 | G protein-coupled receptor 135 | 1.596093996 |
| Samhd1 | SAM domain and HD domain 1 | 1.585325462 |
| Cox5a | cytochrome c oxidase subunit Va | 1.581214255 |
| Arv1 | ARV1 homolog (S. cerevisiae) | 1.578897764 |
| Scarb2 | scavenger receptor class B, member 2 | 1.572103017 |
| Dtx4 | deltex homolog 4 (Drosophila) | 1.570594955 |
| Mrpl52 | mitochondrial ribosomal protein L52 | 1.566407742 |
| Sfrp5 | secreted frizzled-related protein 5 | 1.564587828 |
| Rabac1 | Rab acceptor 1 (prenylated) | 1.561328643 |
| Atpaf2 | ATP synthase mitochondrial F1 complex assembly factor 2 | 1.561084777 |
| Tspan12 | tetraspanin 12 | 1.55791691 |
| Rwdd3 | RWD domain containing 3 | 1.554675125 |
| Epas1 | endothelial PAS domain protein 1 | 1.5533059 |
| Ttr | transthyretin | 1.553244894 |
| Nrg1 | neuregulin 1 | 1.543811641 |
| Agpat1 | 1-acylglycerol-3-phosphate O-acyltransferase 1 (lysophosphatidic acid acyltransferase, alpha) | 1.535441307 |
| Trex1 | three prime repair exonuclease 1 | 1.534986211 |
| Hist1h2ak | histone cluster 1, H2ak | 1.534771609 |
| Pyroxd1 | pyridine nucleotide-disulphide oxidoreductase domain 1 | 1.534559754 |
| Rps2 | ribosomal protein S2 | 1.53308103 |
| Bcat2 | branched chain aminotransferase 2, mitochondrial | 1.524788361 |
| Pcgf3 | polycomb group ring finger 3 | 1.522746208 |
| Cd74 | CD74 molecule, major histocompatibility complex, class II invariant chain | 1.522668943 |
| Sdccag1 | serologically defined colon cancer antigen 1 | 1.52211287 |
| Xpnpep1 | X-prolyl aminopeptidase (aminopeptidase P) 1, soluble | 1.521323779 |
| Rqcd1 | RCD1 required for cell differentiation1 homolog (S. pombe) | 1.518170818 |
| Osmr | oncostatin M receptor | 1.517315418 |
| Hmgcs2 | 3-hydroxy-3-methylglutaryl-Coenzyme A synthase 2 (mitochondrial) | 1.510066746 |
| Ppm1b | protein phosphatase 1B (formerly 2C), magnesium-dependent, beta isoform | 1.50780962 |
| Ephb6 | EPH receptor B6 | 1.506062951 |
| Edg8 | sphingosine-1-phosphate receptor 5 | 1.505941539 |
| Zdhhc1 | zinc finger, DHHC-type containing 1 | 1.503635305 |
| Bmper | BMP binding endothelial regulator | 1.503432071 |
| Mpp5 | membrane protein, palmitoylated 5 (MAGUK p55 subfamily member 5) | 1.502777566 |
| Bmx | BMX non-receptor tyrosine kinase | 1.502031934 |
| Stard8 | StAR-related lipid transfer (START) domain containing 8 | 1.501620712 |
| Grm8 | glutamate receptor, metabotropic 8 | 1.501205166 |
| Smek2 | SMEK homolog 2, suppressor of mek1 (Dictyostelium) | 1.500532105 |
| Rwdd2b | RWD domain containing 2B | -1.500906787 |
| Lrrn2 | leucine rich repeat neuronal 2 | -1.501318694 |
| Dnaja1 | DnaJ (Hsp40) homolog, subfamily A, member 1 | -1.507470379 |
| Adam9 | ADAM metallopeptidase domain 9 (meltrin gamma) | -1.508957204 |
| Trim11 | tripartite motif-containing 11 | -1.509988229 |
| Tomm70a | translocase of outer mitochondrial membrane 70 homolog A (S. cerevisiae) | -1.51916463 |
| Ifi27 | interferon, alpha-inducible protein 27 | -1.520312022 |
| Kcnv1 | potassium channel, subfamily V, member 1 | -1.525249351 |
| Hcfc2 | host cell factor C2 | -1.530196464 |
| Egr4 | early growth response 4 | -1.531712194 |
| Hspa4l | heat shock 70kDa protein 4-like | -1.533189212 |
| Rec8 | REC8 homolog (yeast) | -1.537272326 |
| Ccdc117 | coiled-coil domain containing 117 | -1.540813202 |
| Rin1 | Ras and Rab interactor 1 | -1.543891269 |
| Zfp161 | zinc finger protein 161 homolog (mouse) | -1.547200792 |
| Trim62 | tripartite motif-containing 62 | -1.548039498 |
| Sema3e | sema domain, immunoglobulin domain (Ig), short basic domain, secreted, (semaphorin) 3E | -1.55739415 |
| Tbr1 | T-box, brain, 1 | -1.558327407 |
| Nmd3 | NMD3 homolog (S. cerevisiae) | -1.568678192 |
| H2afj | H2A histone family, member J | -1.578579364 |
| Parp8 | poly (ADP-ribose) polymerase family, member 8 | -1.593110836 |
| Cldn2 | claudin 2 | -1.598038767 |
| Cort | cortistatin | -1.614126044 |
| Snx1 | sorting nexin 1 | -1.61657838 |
| Glra1 | glycine receptor, alpha 1 | -1.710595772 |
| Rpe | ribulose-5-phosphate-3-epimerase | -1.749341094 |

**Table S6.** Hypothalamic gene transcripts differentially expressed in sedentary-caloric restriction (CR) *db/db* mice compared to sedentary-*ad libitum* (AL) *db/db* mice (n=3 animals per group). Statistical significance was considered at p≤0.05.

| **Gene Symbol** | **Description** | **z-ratio (CR vs. AL)** |
| --- | --- | --- |
| Pmch | pro-melanin-concentrating hormone | 5.778343147 |
| Oxt | oxytocin, prepropeptide | 5.69393542 |
| Fpr2 | formyl peptide receptor 2 | 5.688493313 |
| Cuedc1 | CUE domain containing 1 | 4.600468779 |
| Cdkn1a | cyclin-dependent kinase inhibitor 1A (p21, Cip1) | 4.540265989 |
| Avp | arginine vasopressin | 4.522146132 |
| Hcrt | hypocretin (orexin) neuropeptide precursor | 4.410056224 |
| Trim72 | tripartite motif-containing 72 | 4.40136946 |
| Plvap | plasmalemma vesicle associated protein | 4.255858048 |
| Dynll2 | dynein, light chain, LC8-type 2 | 4.211614824 |
| Lyg1 | lysozyme G-like 1 | 4.188403926 |
| Agrp | agouti related protein homolog (mouse) | 4.165960338 |
| Nipsnap1 | nipsnap homolog 1 (C. elegans) | 4.0568529 |
| Sstr1 | somatostatin receptor 1 | 3.970476632 |
| Scrg1 | stimulator of chondrogenesis 1 | 3.96181371 |
| Eef2 | eukaryotic translation elongation factor 2 | 3.949998487 |
| Ccl17 | chemokine (C-C motif) ligand 17 | 3.904231862 |
| Maea | macrophage erythroblast attacher | 3.526368512 |
| Samd5 | sterile alpha motif domain containing 5 | 3.432480819 |
| Fkbp5 | FK506 binding protein 5 | 3.368017669 |
| Itgad | integrin, alpha D | 3.222921561 |
| Siat7f | ST6 (alpha-N-acetyl-neuraminyl-2,3-beta-galactosyl-1,3)-N-acetylgalactosaminide alpha-2,6-sialyltransferase 6 | 3.188248524 |
| Ddx5 | DEAD (Asp-Glu-Ala-Asp) box polypeptide 5 | 3.118623203 |
| Gbas | glioblastoma amplified sequence | 3.116557004 |
| Sult1a1 | sulfotransferase family, cytosolic, 1A, phenol-preferring, member 1 | 3.068774015 |
| Hnrnpa2b1 | heterogeneous nuclear ribonucleoprotein A2/B1 | 3.065031776 |
| Cirbp | cold inducible RNA binding protein | 2.985858112 |
| Arrdc4 | arrestin domain containing 4 | 2.980133554 |
| Fgfr3 | fibroblast growth factor receptor 3 | 2.973436035 |
| Tom1 | target of myb1 (chicken) | 2.963237695 |
| Spock2 | sparc/osteonectin, cwcv and kazal-like domains proteoglycan (testican) 2 | 2.854442954 |
| Nnat | neuronatin | 2.846106148 |
| Actb | actin, beta | 2.845859214 |
| Gal | galanin prepropeptide | 2.812757875 |
| Tsc22d3 | TSC22 domain family, member 3 | 2.739638094 |
| Gsn | gelsolin (amyloidosis, Finnish type) | 2.722710215 |
| Rap1gap | RAP1 GTPase activating protein | 2.720049612 |
| Dctn1 | dynactin 1 (p150, glued homolog, Drosophila) | 2.702788667 |
| Ssbp4 | single stranded DNA binding protein 4 | 2.668730588 |
| Lcn2 | lipocalin 2 | 2.620621414 |
| Lrp6 | low density lipoprotein receptor-related protein 6 | 2.611673482 |
| Gpr19 | G protein-coupled receptor 19 | 2.585401077 |
| Arhgef15 | Rho guanine nucleotide exchange factor (GEF) 15 | 2.563459466 |
| Nfkbia | nuclear factor of kappa light polypeptide gene enhancer in B-cells inhibitor, alpha | 2.546429445 |
| Atg4d | ATG4 autophagy related 4 homolog D (S. cerevisiae) | 2.520630939 |
| Bcl2l1 | BCL2-like 1 | 2.44204791 |
| Ramp3 | receptor (G protein-coupled) activity modifying protein 3 | 2.415225867 |
| Sumo3 | SMT3 suppressor of mif two 3 homolog 3 (S. cerevisiae) | 2.391565795 |
| Igfbp3 | insulin-like growth factor binding protein 3 | 2.379693419 |
| Nubp2 | nucleotide binding protein 2 (MinD homolog, E. coli) | 2.37002651 |
| Pfn1 | profilin 1 | 2.367524142 |
| Per1 | period homolog 1 (Drosophila) | 2.359354138 |
| Wasf3 | WAS protein family, member 3 | 2.295002903 |
| Srrm2 | serine/arginine repetitive matrix 2 | 2.282188578 |
| Cdh13 | cadherin 13, H-cadherin (heart) | 2.270858956 |
| Arhgap27 | Rho GTPase activating protein 27 | 2.26959546 |
| Vps33a | vacuolar protein sorting 33 homolog A (S. cerevisiae) | 2.266472438 |
| Gprc5b | G protein-coupled receptor, family C, group 5, member B | 2.227899642 |
| Ccr6 | chemokine (C-C motif) receptor 6 | 2.197356745 |
| Necap1 | NECAP endocytosis associated 1 | 2.193362942 |
| Map3k6 | mitogen-activated protein kinase kinase kinase 6 | 2.167736183 |
| Txnip | thioredoxin interacting protein | 2.166320667 |
| Slc6a1 | solute carrier family 6 (neurotransmitter transporter, GABA), member 1 | 2.156961162 |
| S100a8 | S100 calcium binding protein A8 | 2.154032529 |
| Gpx3 | glutathione peroxidase 3 (plasma) | 2.150375473 |
| Rab4a | RAB4A, member RAS oncogene family | 2.129357477 |
| Rps6 | ribosomal protein S6 | 2.128025627 |
| Calr3 | calreticulin 3 | 2.121663489 |
| Gbx2 | gastrulation brain homeobox 2 | 2.109572442 |
| C4b | complement component 4B (Chido blood group) | 2.105834299 |
| Agt | angiotensinogen (serpin peptidase inhibitor, clade A, member 8) | 2.092616749 |
| Rac1 | ras-related C3 botulinum toxin substrate 1 (rho family, small GTP binding protein Rac1) | 2.090452048 |
| Tpmt | thiopurine S-methyltransferase | 2.08761864 |
| Pnpo | pyridoxamine 5'-phosphate oxidase | 2.064383214 |
| Ppp1cc | protein phosphatase 1, catalytic subunit, gamma isoform | 2.060573804 |
| Btf3 | basic transcription factor 3 | 2.058819719 |
| Mbp | myelin basic protein | 2.052651906 |
| Pcdh9 | protocadherin 9 | 2.050621242 |
| Rab1b | RAB1B, member RAS oncogene family | 2.046554726 |
| Tsc22d1 | TSC22 domain family, member 1 | 2.045565052 |
| BC005624 | chromosome 9 open reading frame 78 | 2.04060846 |
| Gabbr1 | gamma-aminobutyric acid (GABA) B receptor, 1 | 2.039203858 |
| Armcx5 | armadillo repeat containing, X-linked 5 | 2.032496238 |
| Etv1 | ets variant 1 | 2.024467861 |
| Tdrd6 | tudor domain containing 6 | 2.018512035 |
| Adipor2 | adiponectin receptor 2 | 1.980762652 |
| Tcte3 | t-complex-associated-testis-expressed 3 | 1.980099928 |
| Gramd1a | GRAM domain containing 1A | 1.978854995 |
| Atp6ap2 | ATPase, H+ transporting, lysosomal accessory protein 2 | 1.961042496 |
| Gfer | growth factor, augmenter of liver regeneration | 1.958635323 |
| Pnpla2 | patatin-like phospholipase domain containing 2 | 1.958539325 |
| Rps13 | ribosomal protein S13 | 1.951608199 |
| Nmnat2 | nicotinamide nucleotide adenylyltransferase 2 | 1.951127276 |
| Amn | amnionless homolog (mouse) | 1.936674539 |
| Gnmt | glycine N-methyltransferase | 1.931064534 |
| Sqstm1 | sequestosome 1 | 1.928899991 |
| Spin | spindlin 1 | 1.928299934 |
| Eif4b | eukaryotic translation initiation factor 4B | 1.9201653 |
| Trim35 | tripartite motif-containing 35 | 1.909120735 |
| Slc2a4 | solute carrier family 2 (facilitated glucose transporter), member 4 | 1.900721656 |
| Rpl24 | ribosomal protein L24 | 1.890418822 |
| Txndc9 | thioredoxin domain containing 9 | 1.876163148 |
| Gstcd | glutathione S-transferase, C-terminal domain containing | 1.865693995 |
| Mfsd2 | major facilitator superfamily domain containing 2 | 1.864897465 |
| Usp29 | ubiquitin specific peptidase 29 | 1.86001739 |
| Tle3 | transducin-like enhancer of split 3 (E(sp1) homolog, Drosophila) | 1.851856494 |
| Myo5a | myosin VA (heavy chain 12, myoxin) | 1.843998204 |
| Vamp2 | vesicle-associated membrane protein 2 (synaptobrevin 2) | 1.834222115 |
| Rbbp7 | retinoblastoma binding protein 7 | 1.828351618 |
| Rfng | RFNG O-fucosylpeptide 3-beta-N-acetylglucosaminyltransferase | 1.820967703 |
| Ggt7 | gamma-glutamyltransferase 7 | 1.817221818 |
| Dgat2 | diacylglycerol O-acyltransferase homolog 2 (mouse) | 1.810800821 |
| Cav2 | caveolin 2 | 1.810376569 |
| Mfsd11 | major facilitator superfamily domain containing 11 | 1.803513018 |
| Hint1 | histidine triad nucleotide binding protein 1 | 1.79275917 |
| Acat1 | acetyl-Coenzyme A acetyltransferase 1 | 1.790003107 |
| Ripk5 | dual serine/threonine and tyrosine protein kinase | 1.771509461 |
| Galnt11 | UDP-N-acetyl-alpha-D-galactosamine:polypeptide N-acetylgalactosaminyltransferase 11 (GalNAc-T11) | 1.768395583 |
| Gnb1 | guanine nucleotide binding protein (G protein), beta polypeptide 1 | 1.767642341 |
| Atp6v0e2 | ATPase, H+ transporting V0 subunit e2 | 1.767608388 |
| Elovl4 | elongation of very long chain fatty acids (FEN1/Elo2, SUR4/Elo3, yeast)-like 4 | 1.766797172 |
| Znhit3 | zinc finger, HIT type 3 | 1.761947046 |
| Sf3b4 | splicing factor 3b, subunit 4, 49kDa | 1.749337832 |
| Map3k3 | mitogen-activated protein kinase kinase kinase 3 | 1.745665622 |
| Rpl34 | ribosomal protein L34 | 1.74525568 |
| Mllt11 | myeloid/lymphoid or mixed-lineage leukemia (trithorax homolog, Drosophila); translocated to, 11 | 1.744699544 |
| Rpl18 | ribosomal protein L18 | 1.743266742 |
| Ppp2r1a | protein phosphatase 2 (formerly 2A), regulatory subunit A, alpha isoform | 1.722198199 |
| Eif5a | eukaryotic translation initiation factor 5A | 1.721122605 |
| Apoc1 | apolipoprotein C-I | 1.707456582 |
| Ccbl2 | cysteine conjugate-beta lyase 2 | 1.701267766 |
| Pld3 | phospholipase D family, member 3 | 1.693489964 |
| Nptx2 | neuronal pentraxin II | 1.68974134 |
| Tspan33 | tetraspanin 33 | 1.687609105 |
| Atp5l | ATP synthase, H+ transporting, mitochondrial F0 complex, subunit G | 1.687383764 |
| Htra1 | HtrA serine peptidase 1 | 1.686720701 |
| Apoa1bp | apolipoprotein A-I binding protein | 1.686400829 |
| BC030500 | cDNA sequence BC030500 | 1.684576607 |
| Tspan3 | tetraspanin 3 | 1.677669984 |
| Scube2 | signal peptide, CUB domain, EGF-like 2 | 1.671969566 |
| Gpam | glycerol-3-phosphate acyltransferase, mitochondrial | 1.671485246 |
| Plekha1 | pleckstrin homology domain containing, family A (phosphoinositide binding specific) member 1 | 1.67023701 |
| Itgb1bp1 | integrin beta 1 binding protein 1 | 1.666723656 |
| AI987692 | gasdermin C | 1.666594005 |
| Tmem2 | transmembrane protein 2 | 1.654017107 |
| Gna12 | guanine nucleotide binding protein (G protein) alpha 12 | 1.653984115 |
| Wdr13 | WD repeat domain 13 | 1.645301045 |
| Qsox1 | quiescin Q6 sulfhydryl oxidase 1 | 1.641645576 |
| Pi4kb | phosphatidylinositol 4-kinase, catalytic, beta | 1.638156074 |
| Ppm2c | protein phosphatase 2C, magnesium-dependent, catalytic subunit | 1.635286604 |
| Calb2 | calbindin 2 | 1.631235358 |
| C2 | complement component 2 | 1.629172441 |
| Stk25 | serine/threonine kinase 25 (STE20 homolog, yeast) | 1.628393355 |
| Atp6v1g2 | ATPase, H+ transporting, lysosomal 13kDa, V1 subunit G2 | 1.628159294 |
| Cdan1 | congenital dyserythropoietic anemia, type I | 1.628064327 |
| Usp53 | ubiquitin specific peptidase 53 | 1.625696247 |
| Ppp1r16b | protein phosphatase 1, regulatory (inhibitor) subunit 16B | 1.623633975 |
| Emd | emerin | 1.622088283 |
| Ube2e1 | ubiquitin-conjugating enzyme E2E 1 (UBC4/5 homolog, yeast) | 1.618915962 |
| Zfand2a | zinc finger, AN1-type domain 2A | 1.617476613 |
| Ireb2 | iron-responsive element binding protein 2 | 1.616447699 |
| Itpk1 | inositol 1,3,4-triphosphate 5/6 kinase | 1.612781398 |
| Has1 | hyaluronan synthase 1 | 1.609703477 |
| Gcn5l2 | K(lysine) acetyltransferase 2A | 1.605354098 |
| Rab7 | RAB7A, member RAS oncogene family | 1.604650655 |
| Galc | galactosylceramidase | 1.603035835 |
| Galk1 | galactokinase 1 | 1.602473554 |
| Camkk1 | calcium/calmodulin-dependent protein kinase kinase 1, alpha | 1.602039702 |
| Plekhf1 | pleckstrin homology domain containing, family F (with FYVE domain) member 1 | 1.6000073 |
| Cyb5r3 | cytochrome b5 reductase 3 | 1.592504619 |
| Fcho1 | FCH domain only 1 | 1.59161221 |
| Tmsb10 | thymosin beta 10 | 1.584715144 |
| Adss | adenylosuccinate synthase | 1.583438654 |
| Mcm10 | minichromosome maintenance complex component 10 | 1.577389762 |
| Snap91 | synaptosomal-associated protein, 91kDa homolog (mouse) | 1.577193868 |
| Adrm1 | adhesion regulating molecule 1 | 1.576977743 |
| Apoa1 | apolipoprotein A-I | 1.576098788 |
| Ctgf | connective tissue growth factor | 1.575537078 |
| Scamp5 | secretory carrier membrane protein 5 | 1.572822968 |
| Dnm | dynamin 1 | 1.565904451 |
| Tm4sf5 | transmembrane 4 L six family member 5 | 1.565242785 |
| Mtch1 | mitochondrial carrier homolog 1 (C. elegans) | 1.564713452 |
| Tom1l2 | target of myb1-like 2 (chicken) | 1.562461771 |
| Csnk1d | casein kinase 1, delta | 1.562264213 |
| Pcdh10 | protocadherin 10 | 1.557192422 |
| Adam32 | ADAM metallopeptidase domain 32 | 1.552482837 |
| Cyc1 | cytochrome c-1 | 1.549858584 |
| Leng8 | leukocyte receptor cluster (LRC) member 8 | 1.548130956 |
| Mlf2 | myeloid leukemia factor 2 | 1.545158148 |
| Ppt1 | palmitoyl-protein thioesterase 1 | 1.537242854 |
| Wnt3 | wingless-type MMTV integration site family, member 3 | 1.532628143 |
| Slc6a15 | solute carrier family 6 (neutral amino acid transporter), member 15 | 1.532323256 |
| Plod2 | procollagen-lysine, 2-oxoglutarate 5-dioxygenase 2 | 1.531927854 |
| Churc1 | churchill domain containing 1 | 1.529904702 |
| Guk1 | guanylate kinase 1 | 1.522403408 |
| Cdkl3 | cyclin-dependent kinase-like 3 | 1.52205846 |
| Kremen | kringle containing transmembrane protein 1 | 1.520493066 |
| Ufsp1 | UFM1-specific peptidase 1 (non-functional) | 1.513899394 |
| Tfdp1 | transcription factor Dp-1 | 1.513584716 |
| Kif5b | kinesin family member 5B | 1.510225542 |
| Pik3ip1 | phosphoinositide-3-kinase interacting protein 1 | 1.503455459 |
| Ganc | glucosidase, alpha; neutral C | -1.503986602 |
| Mdga2 | MAM domain containing glycosylphosphatidylinositol anchor 2 | -1.508451996 |
| Anxa5 | annexin A5 | -1.508960779 |
| Vrk3 | vaccinia related kinase 3 | -1.509600974 |
| Usp33 | ubiquitin specific peptidase 33 | -1.510841747 |
| Sh3kbp1 | SH3-domain kinase binding protein 1 | -1.511811238 |
| Smek2 | SMEK homolog 2, suppressor of mek1 (Dictyostelium) | -1.514482564 |
| Samd4 | sterile alpha motif domain containing 4A | -1.514717366 |
| Cbx5 | chromobox homolog 5 (HP1 alpha homolog, Drosophila) | -1.517924324 |
| Abcd2 | ATP-binding cassette, sub-family D (ALD), member 2 | -1.518137254 |
| Nrgn | neurogranin (protein kinase C substrate, RC3) | -1.518674821 |
| Hn1l | hematological and neurological expressed 1-like | -1.519043142 |
| Mettl3 | methyltransferase like 3 | -1.520078241 |
| Hspa2 | heat shock 70kDa protein 2 | -1.521330679 |
| Lsm12 | LSM12 homolog (S. cerevisiae) | -1.523631435 |
| Rgl2 | ral guanine nucleotide dissociation stimulator-like 2 | -1.527415221 |
| Mnat1 | menage a trois homolog 1, cyclin H assembly factor (Xenopus laevis) | -1.534260589 |
| Tnfrsf19 | tumor necrosis factor receptor superfamily, member 19 | -1.534300089 |
| Supt3h | suppressor of Ty 3 homolog (S. cerevisiae) | -1.536513081 |
| Arc | activity-regulated cytoskeleton-associated protein | -1.539391414 |
| Ephb1 | EPH receptor B1 | -1.540683956 |
| Dusp23 | dual specificity phosphatase 23 | -1.541116122 |
| Irx3 | iroquois homeobox 3 | -1.546029466 |
| Tcf4 | transcription factor 4 | -1.546270595 |
| Accn2 | amiloride-sensitive cation channel 2, neuronal | -1.546596393 |
| Evi2a | ecotropic viral integration site 2A | -1.548175805 |
| Acss1 | acyl-CoA synthetase short-chain family member 1 | -1.550688503 |
| Snx3 | sorting nexin 3 | -1.552262977 |
| Gal3st1 | galactose-3-O-sulfotransferase 1 | -1.554921991 |
| Tmem42 | transmembrane protein 42 | -1.555776682 |
| Rxfp3 | relaxin/insulin-like family peptide receptor 3 | -1.557394701 |
| Tsc2 | tuberous sclerosis 2 | -1.558518302 |
| Slc7a11 | solute carrier family 7, (cationic amino acid transporter, y+ system) member 11 | -1.559761973 |
| Spred1 | sprouty-related, EVH1 domain containing 1 | -1.570243932 |
| Tax1bp1 | Tax1 (human T-cell leukemia virus type I) binding protein 1 | -1.570886425 |
| Txnl4 | thioredoxin-like 4A | -1.571156546 |
| Asah2 | N-acylsphingosine amidohydrolase (non-lysosomal ceramidase) 2 | -1.572829362 |
| Pdgfra | platelet-derived growth factor receptor, alpha polypeptide | -1.574207848 |
| Rpl4 | ribosomal protein L4 | -1.575018117 |
| Zdhhc9 | zinc finger, DHHC-type containing 9 | -1.575588114 |
| Fahd1 | fumarylacetoacetate hydrolase domain containing 1 | -1.576561589 |
| Ppid | peptidylprolyl isomerase D | -1.578638153 |
| Gabrb1 | gamma-aminobutyric acid (GABA) A receptor, beta 1 | -1.580782045 |
| Htra3 | HtrA serine peptidase 3 | -1.581366404 |
| Sdccag1 | serologically defined colon cancer antigen 1 | -1.586407653 |
| Zmiz1 | zinc finger, MIZ-type containing 1 | -1.594321136 |
| Ptprk | protein tyrosine phosphatase, receptor type, K | -1.595643042 |
| Tmc7 | transmembrane channel-like 7 | -1.595656258 |
| Ubxd8 | Fas associated factor family member 2 | -1.59661247 |
| Kcng4 | potassium voltage-gated channel, subfamily G, member 4 | -1.596718019 |
| Bicd2 | bicaudal D homolog 2 (Drosophila) | -1.596977933 |
| Syt1 | synaptotagmin I | -1.597573715 |
| Odz3 | odz, odd Oz/ten-m homolog 3 (Drosophila) | -1.600375488 |
| Pa2g4 | proliferation-associated 2G4, 38kDa | -1.60037819 |
| Schip1 | schwannomin interacting protein 1 | -1.600960702 |
| Nubp1 | nucleotide binding protein 1 (MinD homolog, E. coli) | -1.601748657 |
| Luzp2 | leucine zipper protein 2 | -1.60527555 |
| Enpp1 | ectonucleotide pyrophosphatase/phosphodiesterase 1 | -1.610779995 |
| Sox18 | SRY (sex determining region Y)-box 18 | -1.611540045 |
| Crtc3 | CREB regulated transcription coactivator 3 | -1.61379375 |
| Odz4 | odz, odd Oz/ten-m homolog 4 (Drosophila) | -1.619099788 |
| Kcnu1 | potassium channel, subfamily U, member 1 | -1.619869577 |
| Hsd17b12 | hydroxysteroid (17-beta) dehydrogenase 12 | -1.620521128 |
| Hpcal4 | hippocalcin like 4 | -1.622198114 |
| Tmem98 | transmembrane protein 98 | -1.622212555 |
| Wwc1 | WW and C2 domain containing 1 | -1.624203335 |
| Zhx2 | zinc fingers and homeoboxes 2 | -1.629949999 |
| Atp5sl | ATP5S-like | -1.630220363 |
| Gnptg | N-acetylglucosamine-1-phosphate transferase, gamma subunit | -1.63154083 |
| Bcs1l | BCS1-like (yeast) | -1.632037392 |
| Mmrn2 | multimerin 2 | -1.634791045 |
| Exdl2 | exonuclease 3'-5' domain containing 2 | -1.638851181 |
| Caprin1 | cell cycle associated protein 1 | -1.640653536 |
| Rasl12 | RAS-like, family 12 | -1.647567297 |
| Dlgap2 | discs, large (Drosophila) homolog-associated protein 2 | -1.660116484 |
| Gad1 | glutamate decarboxylase 1 (brain, 67kDa) | -1.660368419 |
| Fmn2 | formin 2 | -1.66526545 |
| Glt25d1 | glycosyltransferase 25 domain containing 1 | -1.667369349 |
| Rbm4b | RNA binding motif protein 4B | -1.66851029 |
| Tppp | tubulin polymerization promoting protein | -1.668716129 |
| Phlda1 | pleckstrin homology-like domain, family A, member 1 | -1.670804006 |
| Gbp3 | guanylate binding protein 3 | -1.671495572 |
| Tst | thiosulfate sulfurtransferase (rhodanese) | -1.674365605 |
| Gcc2 | GRIP and coiled-coil domain containing 2 | -1.686734995 |
| Agpat4 | 1-acylglycerol-3-phosphate O-acyltransferase 4 (lysophosphatidic acid acyltransferase, delta) | -1.687865816 |
| Rtn4ip1 | reticulon 4 interacting protein 1 | -1.689325252 |
| Dgkb | diacylglycerol kinase, beta 90kDa | -1.689614961 |
| Elovl6 | ELOVL family member 6, elongation of long chain fatty acids (FEN1/Elo2, SUR4/Elo3-like, yeast) | -1.693536776 |
| Lphn3 | latrophilin 3 | -1.694052423 |
| Loxl1 | lysyl oxidase-like 1 | -1.696399214 |
| Fcgrt | Fc fragment of IgG, receptor, transporter, alpha | -1.698533213 |
| Dusp16 | dual specificity phosphatase 16 | -1.700832487 |
| Brp44 | brain protein 44 | -1.701502503 |
| Cept1 | choline/ethanolamine phosphotransferase 1 | -1.719441784 |
| Asf1a | ASF1 anti-silencing function 1 homolog A (S. cerevisiae) | -1.72477997 |
| Samd9l | sterile alpha motif domain containing 9-like | -1.72768447 |
| Cisd1 | CDGSH iron sulfur domain 1 | -1.730100939 |
| Tmem108 | transmembrane protein 108 | -1.736488997 |
| Ibtk | inhibitor of Bruton agammaglobulinemia tyrosine kinase | -1.741151321 |
| Gata3 | GATA binding protein 3 | -1.74290829 |
| B3galt6 | UDP-Gal:betaGal beta 1,3-galactosyltransferase polypeptide 6 | -1.746540995 |
| Papola | poly(A) polymerase alpha | -1.755155955 |
| Mboat1 | membrane bound O-acyltransferase domain containing 1 | -1.755434843 |
| En2 | engrailed homeobox 2 | -1.757226041 |
| Zdhhc14 | zinc finger, DHHC-type containing 14 | -1.764516602 |
| Grm8 | glutamate receptor, metabotropic 8 | -1.766830745 |
| Bnip3l | BCL2/adenovirus E1B 19kDa interacting protein 3-like | -1.76693295 |
| Rhob | ras homolog gene family, member B | -1.768045199 |
| Adcy4 | adenylate cyclase 4 | -1.777518422 |
| Zmat3 | zinc finger, matrin type 3 | -1.779213423 |
| Acot11 | acyl-CoA thioesterase 11 | -1.780688723 |
| Rgma | RGM domain family, member A | -1.783106526 |
| Evl | Enah/Vasp-like | -1.788030829 |
| Nsd1 | nuclear receptor binding SET domain protein 1 | -1.791584494 |
| Serpind1 | serpin peptidase inhibitor, clade D (heparin cofactor), member 1 | -1.797470574 |
| BC064033 | family with sequence similarity 180, member A | -1.79944582 |
| Impact | Impact homolog (mouse) | -1.805389847 |
| Skiv2l2 | superkiller viralicidic activity 2-like 2 (S. cerevisiae) | -1.807217253 |
| Agpat5 | 1-acylglycerol-3-phosphate O-acyltransferase 5 (lysophosphatidic acid acyltransferase, epsilon) | -1.807842637 |
| Bgn | biglycan | -1.81398805 |
| Hist2h2ac | histone cluster 2, H2ac | -1.816756985 |
| Entpd4 | ectonucleoside triphosphate diphosphohydrolase 4 | -1.825420422 |
| Mas1 | MAS1 oncogene | -1.828879183 |
| Il6st | interleukin 6 signal transducer (gp130, oncostatin M receptor) | -1.830544993 |
| Ccdc124 | coiled-coil domain containing 124 | -1.842107595 |
| Cartpt | CART prepropeptide | -1.855152455 |
| Isyna1 | inositol-3-phosphate synthase 1 | -1.858766201 |
| Adamtsl4 | ADAMTS-like 4 | -1.867794944 |
| Socs5 | suppressor of cytokine signaling 5 | -1.86949451 |
| Stk4 | serine/threonine kinase 4 | -1.869941728 |
| Ky | kyphoscoliosis peptidase | -1.876970306 |
| Cab39l | calcium binding protein 39-like | -1.877206168 |
| Ntsr2 | neurotensin receptor 2 | -1.884837632 |
| Negr1 | neuronal growth regulator 1 | -1.888729853 |
| Ankrd12 | ankyrin repeat domain 12 | -1.889456858 |
| Cd99l2 | CD99 molecule-like 2 | -1.890387434 |
| Ddo | D-aspartate oxidase | -1.894311455 |
| BC049806 | family with sequence similarity 126, member B | -1.904116808 |
| Crtc1 | CREB regulated transcription coactivator 1 | -1.914898303 |
| Leo1 | Leo1, Paf1/RNA polymerase II complex component, homolog (S. cerevisiae) | -1.921866424 |
| Wnt7a | wingless-type MMTV integration site family, member 7A | -1.932826236 |
| Nmral1 | NmrA-like family domain containing 1 | -1.935709117 |
| Slc7a4 | solute carrier family 7 (cationic amino acid transporter, y+ system), member 4 | -1.938053211 |
| Cnih4 | cornichon homolog 4 (Drosophila) | -1.941681448 |
| Nat14 | N-acetyltransferase 14 (GCN5-related, putative) | -1.958449513 |
| Gbf1 | golgi-specific brefeldin A resistant guanine nucleotide exchange factor 1 | -1.963151977 |
| Rdh5 | retinol dehydrogenase 5 (11-cis/9-cis) | -1.967033955 |
| Gramd1b | GRAM domain containing 1B | -1.97503207 |
| Chn2 | chimerin (chimaerin) 2 | -1.982357826 |
| Ndfip2 | Nedd4 family interacting protein 2 | -1.990689403 |
| Nudt10 | nudix (nucleoside diphosphate linked moiety X)-type motif 10 | -1.994316 |
| Cox6c | cytochrome c oxidase subunit VIc | -1.99989139 |
| Ppargc1a | peroxisome proliferator-activated receptor gamma, coactivator 1 alpha | -2.000039692 |
| Itgb4 | integrin, beta 4 | -2.009220109 |
| Klhl9 | kelch-like 9 (Drosophila) | -2.012824534 |
| Slain1 | SLAIN motif family, member 1 | -2.023730844 |
| Ahdc1 | AT hook, DNA binding motif, containing 1 | -2.024230754 |
| Cachd1 | cache domain containing 1 | -2.026751178 |
| Heatr5a | HEAT repeat containing 5A | -2.040391273 |
| Mrpl55 | mitochondrial ribosomal protein L55 | -2.05135918 |
| Dut | deoxyuridine triphosphatase | -2.059694074 |
| Dci | dodecenoyl-Coenzyme A delta isomerase (3,2 trans-enoyl-Coenzyme A isomerase) | -2.068614102 |
| Emp2 | epithelial membrane protein 2 | -2.07541618 |
| Kcne1l | KCNE1-like | -2.075544745 |
| Cx3cr1 | chemokine (C-X3-C motif) receptor 1 | -2.079440655 |
| Sipa1l1 | signal-induced proliferation-associated 1 like 1 | -2.084087355 |
| Afap1 | actin filament associated protein 1 | -2.107962589 |
| Rbmx | RNA binding motif protein, X-linked | -2.116961441 |
| Mef2c | myocyte enhancer factor 2C | -2.118518915 |
| Gamt | guanidinoacetate N-methyltransferase | -2.126061739 |
| Nit1 | nitrilase 1 | -2.127476608 |
| Cmtm5 | CKLF-like MARVEL transmembrane domain containing 5 | -2.14514839 |
| Grin1 | glutamate receptor, ionotropic, N-methyl D-aspartate 1 | -2.149157891 |
| Dek | DEK oncogene | -2.163788333 |
| Zfp91 | zinc finger protein 91 homolog (mouse) | -2.183750246 |
| Ide | insulin-degrading enzyme | -2.187308345 |
| Pes1 | pescadillo homolog 1, containing BRCT domain (zebrafish) | -2.188505275 |
| Megf9 | multiple EGF-like-domains 9 | -2.189319513 |
| Robo1 | roundabout, axon guidance receptor, homolog 1 (Drosophila) | -2.197812636 |
| Zmynd11 | zinc finger, MYND domain containing 11 | -2.200779248 |
| Chordc1 | cysteine and histidine-rich domain (CHORD)-containing 1 | -2.203774027 |
| Plekha2 | pleckstrin homology domain containing, family A (phosphoinositide binding specific) member 2 | -2.20648278 |
| Prkaa2 | protein kinase, AMP-activated, alpha 2 catalytic subunit | -2.207376464 |
| Lman2l | lectin, mannose-binding 2-like | -2.21064808 |
| Hsp90b1 | heat shock protein 90kDa beta (Grp94), member 1 | -2.215345868 |
| Rsad2 | radical S-adenosyl methionine domain containing 2 | -2.216269585 |
| Kcnk13 | potassium channel, subfamily K, member 13 | -2.221493904 |
| Ttc14 | tetratricopeptide repeat domain 14 | -2.236944022 |
| Chrm2 | cholinergic receptor, muscarinic 2 | -2.241333575 |
| Slco1c1 | solute carrier organic anion transporter family, member 1C1 | -2.249423807 |
| Ints7 | integrator complex subunit 7 | -2.250011781 |
| Sat2 | spermidine/spermine N1-acetyltransferase family member 2 | -2.262698395 |
| Rcor1 | REST corepressor 1 | -2.289968871 |
| Zeb2 | zinc finger E-box binding homeobox 2 | -2.297265377 |
| Kcnh1 | potassium voltage-gated channel, subfamily H (eag-related), member 1 | -2.300645499 |
| Purb | purine-rich element binding protein B | -2.305216349 |
| Unc5b | unc-5 homolog B (C. elegans) | -2.311888963 |
| Aff4 | AF4/FMR2 family, member 4 | -2.32577783 |
| Large | like-glycosyltransferase | -2.341496451 |
| Pde1a | phosphodiesterase 1A, calmodulin-dependent | -2.362688464 |
| Pigq | phosphatidylinositol glycan anchor biosynthesis, class Q | -2.37178802 |
| Ifitm3 | interferon induced transmembrane protein 3 (1-8U) | -2.372431872 |
| Ptprt | protein tyrosine phosphatase, receptor type, T | -2.372967591 |
| AB182283 | NAC alpha domain containing | -2.394772024 |
| Tbc1d9b | TBC1 domain family, member 9B (with GRAM domain) | -2.404094346 |
| Myoc | myocilin, trabecular meshwork inducible glucocorticoid response | -2.412418813 |
| Usp18 | ubiquitin specific peptidase 18 | -2.415185781 |
| Sec22c | SEC22 vesicle trafficking protein homolog C (S. cerevisiae) | -2.42815528 |
| AI504432 | expressed sequence AI504432 | -2.433318165 |
| Ptges3 | prostaglandin E synthase 3 (cytosolic) | -2.440268407 |
| Cd82 | CD82 molecule | -2.452297111 |
| Lsamp | limbic system-associated membrane protein | -2.452336645 |
| Gbp2 | guanylate binding protein 2, interferon-inducible | -2.457917998 |
| Ddx6 | DEAD (Asp-Glu-Ala-Asp) box polypeptide 6 | -2.459907936 |
| Upf1 | UPF1 regulator of nonsense transcripts homolog (yeast) | -2.46109627 |
| Josd2 | Josephin domain containing 2 | -2.512227371 |
| Selplg | selectin P ligand | -2.513150347 |
| P2ry13 | purinergic receptor P2Y, G-protein coupled, 13 | -2.519800859 |
| Klk6 | kallikrein-related peptidase 6 | -2.522325356 |
| Hist1h2bc | histone cluster 1, H2bc | -2.531716025 |
| Bmpr1b | bone morphogenetic protein receptor, type IB | -2.542795257 |
| Lcat | lecithin-cholesterol acyltransferase | -2.545139919 |
| Pgpep1 | pyroglutamyl-peptidase I | -2.570121357 |
| Laptm5 | lysosomal multispanning membrane protein 5 | -2.581981267 |
| Kctd4 | potassium channel tetramerisation domain containing 4 | -2.583737506 |
| Gas7 | growth arrest-specific 7 | -2.594293652 |
| Dcp1b | DCP1 decapping enzyme homolog B (S. cerevisiae) | -2.617894049 |
| Slc38a5 | solute carrier family 38, member 5 | -2.635524689 |
| Crocc | ciliary rootlet coiled-coil, rootletin | -2.647743741 |
| Trim9 | tripartite motif-containing 9 | -2.704230502 |
| Dgkk | diacylglycerol kinase, kappa | -2.712267524 |
| Ppp1r14a | protein phosphatase 1, regulatory (inhibitor) subunit 14A | -2.712812492 |
| Zbtb22 | zinc finger and BTB domain containing 22 | -2.732039798 |
| Gdf9 | growth differentiation factor 9 | -2.737318948 |
| Tmem70 | transmembrane protein 70 | -2.750017244 |
| Dap3 | death associated protein 3 | -2.756134607 |
| Efcab1 | EF-hand calcium binding domain 1 | -2.794616993 |
| Mllt3 | myeloid/lymphoid or mixed-lineage leukemia (trithorax homolog, Drosophila); translocated to, 3 | -2.80599561 |
| Mrps23 | mitochondrial ribosomal protein S23 | -2.809502946 |
| Syn2 | synapsin II | -2.812430339 |
| Mid1 | midline 1 (Opitz/BBB syndrome) | -2.815501128 |
| Mboat2 | membrane bound O-acyltransferase domain containing 2 | -2.856084657 |
| Hist1h2be | histone cluster 1, H2be | -2.865007438 |
| Aqp4 | aquaporin 4 | -2.8795326 |
| Slc5a5 | solute carrier family 5 (sodium iodide symporter), member 5 | -2.884731234 |
| Ccnd2 | cyclin D2 | -2.90664584 |
| Ccdc85a | coiled-coil domain containing 85A | -2.936064778 |
| Aebp1 | AE binding protein 1 | -3.006539867 |
| Pkd2l1 | polycystic kidney disease 2-like 1 | -3.05003451 |
| Kif21a | kinesin family member 21A | -3.111894832 |
| Prelp | proline/arginine-rich end leucine-rich repeat protein | -3.126548745 |
| Gkap1 | G kinase anchoring protein 1 | -3.192490451 |
| Deadc1 | adenosine deaminase, tRNA-specific 2, TAD2 homolog (S. cerevisiae) | -3.273742988 |
| Napepld | N-acyl phosphatidylethanolamine phospholipase D | -3.273989815 |
| Tbl1x | transducin (beta)-like 1X-linked | -3.275035465 |
| Thsd4 | thrombospondin, type I, domain containing 4 | -3.325198841 |
| Fjx1 | four jointed box 1 (Drosophila) | -3.334334481 |
| Ddit4l | DNA-damage-inducible transcript 4-like | -3.352597506 |
| Wdr46 | WD repeat domain 46 | -3.366777096 |
| Tex9 | testis expressed 9 | -3.370194981 |
| Btbd3 | BTB (POZ) domain containing 3 | -3.371530312 |
| Kcnd2 | potassium voltage-gated channel, Shal-related subfamily, member 2 | -3.415640264 |
| Slc6a4 | solute carrier family 6 (neurotransmitter transporter, serotonin), member 4 | -3.502669078 |
| Eif1b | eukaryotic translation initiation factor 1B | -3.519365916 |
| Nell2 | NEL-like 2 (chicken) | -3.630852452 |
| Mgat3 | mannosyl (beta-1,4-)-glycoprotein beta-1,4-N-acetylglucosaminyltransferase | -3.753666966 |
| Gps2 | G protein pathway suppressor 2 | -3.802955701 |
| Atf7ip | activating transcription factor 7 interacting protein | -3.812667285 |
| Odz1 | odz, odd Oz/ten-m homolog 1(Drosophila) | -3.886422446 |
| Wdfy3 | WD repeat and FYVE domain containing 3 | -3.942721157 |
| Arhgef19 | Rho guanine nucleotide exchange factor (GEF) 19 | -3.946752142 |
| Lyrm2 | LYR motif containing 2 | -3.980284294 |
| Axud1 | cysteine-serine-rich nuclear protein 1 | -3.986137478 |
| Ddr1 | discoidin domain receptor tyrosine kinase 1 | -3.993533807 |
| Tmem10 | oligodendrocytic myelin paranodal and inner loop protein | -4.231291057 |
| Ccdc117 | coiled-coil domain containing 117 | -4.268767794 |
| Trim62 | tripartite motif-containing 62 | -4.413576927 |
| Rec8 | REC8 homolog (yeast) | -4.480056797 |
| Il17d | interleukin 17D | -4.622191945 |
| Kcna1 | potassium voltage-gated channel, shaker-related subfamily, member 1 (episodic ataxia with myokymia) | -4.628359844 |
| Atf4 | activating transcription factor 4 (tax-responsive enhancer element B67) | -4.892771245 |
| Fgfr1op2 | FGFR1 oncogene partner 2 | -4.949682852 |
| Tph2 | tryptophan hydroxylase 2 | -5.319231037 |
| Glra1 | glycine receptor, alpha 1 | -5.626848515 |
| Scn1a | sodium channel, voltage-gated, type I, alpha subunit | -5.790848196 |
| H2afj | H2A histone family, member J | -5.873413123 |
| Kif3a | kinesin family member 3A | -6.017381642 |

**Table S7.** Hypothalamic gene transcripts commonly regulated by running in WT and *db/db* *ad libitum* fed mice (n=3 animals per group). Statistical significance was considered at p≤0.05.

| **Common gene symbol** | **WT z ratio** | ***db/db* z ratio** |
| --- | --- | --- |
| LOC100041504 | 3.507954841 | 1.55438728 |
| Wdr46 | 1.766822947 | 1.578235501 |
| Ccl21c | 1.587010291 | 1.568851522 |
|  |  |  |
| 9930021D14Rik | -1.568779712 | -1.567627248 |
|  |  |  |
| Sstr1 | -1.792199969 | -3.273337483 |
|  |  |  |
| Bmp7 | 4.645192484 | -3.541157737 |
| Rft1 | 1.945283255 | -4.997303484 |
| Msn | 3.50285555 | -3.148363859 |
| Gsta3 | 2.422222084 | -3.659313295 |
| Foxred2 | 3.013374739 | -2.764393053 |
| Accs | 1.886722362 | -3.842835679 |
| Nedd1 | 3.323035734 | -2.330143771 |
| Leng1 | 3.522958468 | -1.988163098 |
| AU022252 | 2.941282567 | -2.538261786 |
| B4galt7 | 1.929244896 | -3.448316788 |
| Ccdc71 | 2.190905778 | -3.011537589 |
| Zfp629 | 2.257636998 | -2.890679531 |
| Nrg3 | 3.04645592 | -2.056868999 |
| BC031575 | 3.590200489 | -1.509031149 |
| B930041G04 | 2.466040381 | -2.562222055 |
| E130016E03Rik | 2.221481846 | -2.798468153 |
| E4f1 | 3.030452138 | -1.955845678 |
| D8Ertd82e | 1.661961312 | -3.317427044 |
| Ipp | 2.222946183 | -2.631515883 |
| Arbp | 2.855885486 | -1.948405893 |
| Ckb | 2.172004926 | -2.536701189 |
| Ubb | 2.204047753 | -2.493332716 |
| Rplp1 | 2.23937476 | -2.427401896 |
| Sh3bp2 | 2.762299673 | -1.783141545 |
| Icam2 | 2.251739143 | -2.229634661 |
| Atp5b | 2.099431431 | -2.328868544 |
| Poli | 2.790931462 | -1.610598081 |
| Tuba1b | 2.126044749 | -2.273931639 |
| 2210417D09Rik | 2.566286786 | -1.827757333 |
| Xrn1 | 2.267937297 | -2.06951996 |
| Gm1821 | 2.060242786 | -2.272944578 |
| L3mbtl2 | 1.909001961 | -2.391524356 |
| Pwwp2b | 1.734684118 | -2.564932155 |
| Rgs6 | 2.373955573 | -1.894128235 |
| Ephb2 | 1.990485573 | -2.265262291 |
| 5830415L20Rik | 1.613484472 | -2.635393053 |
| Mt1 | 1.983573202 | -2.242920562 |
| Olfr873 | 2.408525382 | -1.797494006 |
| LOC218963 | 2.044533649 | -2.139537412 |
| Gprk6 | 1.652468323 | -2.502753545 |
| Psmc3ip | 1.833009443 | -2.320153129 |
| Pcdhb22 | 2.076428405 | -2.076372581 |
| Bet1 | 2.255830034 | -1.89446351 |
| Rpl41 | 2.001609669 | -2.141462993 |
| Rin3 | 2.038592812 | -2.096403526 |
| LOC100045981 | 1.996459671 | -2.122621373 |
| Syt3 | 1.876875324 | -2.222291113 |
| Nfx1 | 2.001701914 | -2.092444865 |
| Dgat1 | 2.100536143 | -1.987855097 |
| Nit2 | 2.011762955 | -2.073393052 |
| 2010107G12Rik | 2.556234464 | -1.517611541 |
| Atp1b1 | 2.063465353 | -1.996812533 |
| Zbtb2 | 1.737740096 | -2.321460397 |
| Ppp1r12c | 1.897605697 | -2.136362357 |
| Fbxl5 | 1.666802893 | -2.348197233 |
| Ifrg15 | 1.971715889 | -2.033970513 |
| Rpgrip1l | 2.02555082 | -1.975784098 |
| Atp5g3 | 1.961851295 | -2.030833152 |
| C78339 | 1.797256841 | -2.18862705 |
| Fth1 | 2.111743737 | -1.871257752 |
| Cplx1 | 1.955702047 | -1.993837193 |
| Dicer1 | 2.13429471 | -1.775909798 |
| Ppia | 1.874524402 | -2.035478946 |
| Rpl37 | 1.943376106 | -1.948844102 |
| Prnp | 1.87921805 | -1.973156759 |
| LOC100042427 | 1.843252797 | -1.967687077 |
| Eef1a1 | 1.863262688 | -1.900948242 |
| Hnrpf | 2.122393623 | -1.636120261 |
| Mxra7 | 2.199865116 | -1.548621493 |
| Gab3 | 1.994756541 | -1.722694772 |
| LOC666904 | 1.960917443 | -1.755836863 |
| Cox5b | 1.822404769 | -1.853217483 |
| Rccd1 | 2.092921535 | -1.575928855 |
| Cst3 | 1.867898504 | -1.781219874 |
| Gpr68 | 1.790235297 | -1.854075697 |
| Slc25a45 | 1.76207153 | -1.870902757 |
| Grina | 1.774608546 | -1.85066947 |
| Ctsk | 1.612815218 | -1.965229592 |
| Prkacb | 1.694533127 | -1.876019136 |
| Rpl9 | 1.684038577 | -1.864043695 |
| AA691260 | 1.923069703 | -1.616816835 |
| 1600021P15Rik | 1.841922594 | -1.687548935 |
| LOC100048483 | 1.696151038 | -1.814669485 |
| Cox6a1 | 1.592895296 | -1.912374945 |
| EG622339 | 1.74011097 | -1.764737092 |
| Cox4i1 | 1.694501368 | -1.807875803 |
| Bcas1 | 1.774835885 | -1.718390607 |
| Mif | 1.626961387 | -1.865239622 |
| Rab7l1 | 1.785977392 | -1.693450949 |
| Calm3 | 1.672985562 | -1.805073745 |
| Polb | 1.59332919 | -1.87796493 |
| Pdss1 | 1.566726966 | -1.899819638 |
| Plcb3 | 1.665094632 | -1.794302223 |
| U2af1l4 | 1.50399033 | -1.946105658 |
| Ndufb8 | 1.610053101 | -1.807969135 |
| Wbp2 | 1.61050408 | -1.799947866 |
| Mina | 1.664396497 | -1.744827908 |
| D4Wsu114e | 1.829421422 | -1.578106092 |
| Arhgap22 | 1.52475185 | -1.88105396 |
| Nsf | 1.709757051 | -1.67549829 |
| Crhr1 | 1.659739799 | -1.690417675 |
| Atp5j2 | 1.603533966 | -1.746120918 |
| Rps12 | 1.583433252 | -1.765066095 |
| Ndufa13 | 1.652980208 | -1.683667646 |
| Med11 | 1.570761765 | -1.762696357 |
| 3335299200 | 1.622228287 | -1.701799583 |
| Hnrph3 | 1.599937416 | -1.723691878 |
| Ccdc123 | 1.791646911 | -1.526409489 |
| LOC675813 | 1.588580732 | -1.725818476 |
| Prrg2 | 1.691571451 | -1.621952322 |
| Wdr5 | 1.594887688 | -1.715144857 |
| Tmem132c | 1.545307851 | -1.758004708 |
| 6330403K07Rik | 1.54977137 | -1.751890079 |
| Eno2 | 1.599259644 | -1.697738667 |
| Gper | 1.544318043 | -1.747336383 |
| Rpl35 | 1.544893946 | -1.724121883 |
| Thy1 | 1.628493805 | -1.617285624 |
| Rps14 | 1.544526701 | -1.676388988 |
| 2810021B07Rik | 1.517892999 | -1.700278786 |
| LOC100045950 | 1.616736508 | -1.59793754 |
| Cln6 | 1.534750876 | -1.639713575 |
| Hba-a1 | 1.546399058 | -1.627892691 |
| Ccdc111 | 1.662818631 | -1.509921244 |
| Atp6v1e1 | 1.583313979 | -1.587517501 |
| Cd81 | 1.596308318 | -1.533160474 |
| Plp1 | 1.567305102 | -1.561305427 |
| Cldn11 | 1.555455616 | -1.571855674 |
| Rps8 | 1.511263685 | -1.615932118 |
| LOC381801 | 1.534984344 | -1.583669999 |
| EG668668 | 1.529873756 | -1.576057982 |
| Clic6 | 1.523611055 | -1.581810365 |
| Plekhb1 | 1.522147784 | -1.542786287 |
| Rab11b | 1.508594181 | -1.54055148 |
| Vsnl1 | 1.525901969 | -1.513716882 |
| Syp | 1.518346149 | -1.519477504 |
| Aatk | 1.529703174 | -1.506677088 |
|  |  |  |
| Pmch | -2.249531924 | 1.780573964 |
| Cga | -1.893854102 | 1.75819492 |
| Cyp2c37 | -1.907109653 | 1.718758702 |
| Capsl | -1.797594226 | 1.816024564 |
| Mpo | -1.95653686 | 1.64572848 |
| Gbp1 | -1.895925573 | 1.689065816 |
| V1rc24 | -1.901643992 | 1.677983985 |
| Klk1b24 | -1.841985865 | 1.712725518 |
| Tshb | -1.872352179 | 1.659433802 |
| Prok2 | -1.830257258 | 1.695386877 |
| Igbp1b | -1.812436813 | 1.706903546 |
| Trrap | -1.771825708 | 1.722407189 |
| Zfp54 | -1.783414374 | 1.704444997 |
| Slitrk6 | -1.819955901 | 1.66620942 |
| 2010005H15Rik | -1.835922528 | 1.64994797 |
| Sh3rf2 | -1.802569442 | 1.683265426 |
| Zfa | -1.831083623 | 1.653826327 |
| Calcr | -1.787691218 | 1.683714635 |
| Crabp1 | -1.774273422 | 1.682347995 |
| Klk1b27 | -1.854075722 | 1.599886416 |
| Gm608 | -1.800416096 | 1.6327768 |
| Plunc | -1.801921647 | 1.628358247 |
| EG433016 | -1.790702074 | 1.618441163 |
| LOC100044133 | -1.815226562 | 1.590180696 |
| Kcna5 | -1.771293924 | 1.633714628 |
| C130057D23Rik | -1.792305772 | 1.608965316 |
| Kiss1 | -1.826389453 | 1.573482255 |
| Synpo2 | -1.827626841 | 1.554797388 |
| Pomc | -1.730674421 | 1.651234343 |
| 4933434I06Rik | -1.691605248 | 1.664787758 |
| Olfr913 | -1.748356199 | 1.606483527 |
| Gm428 | -1.736101571 | 1.611638195 |
| Agtr1a | -1.769475274 | 1.568571322 |
| Esr1 | -1.683999402 | 1.651273374 |
| Bglap2 | -1.690802451 | 1.643968481 |
| 1700003M02Rik | -1.665155096 | 1.668481476 |
| Map2k1 | -1.65218958 | 1.673135007 |
| Brs3 | -1.69333022 | 1.62599473 |
| Mylc2pl | -1.71892675 | 1.590865037 |
| Mro | -1.666632334 | 1.638882523 |
| Cldn23 | -1.68109847 | 1.624219093 |
| Npvf | -1.772901757 | 1.529718641 |
| Dlx2 | -1.70631079 | 1.595745648 |
| LOC667250 | -1.6038959 | 1.691653522 |
| Stk22a | -1.744063601 | 1.548770503 |
| Kti12 | -1.629013867 | 1.659486036 |
| Ttc15 | -1.749239938 | 1.538384195 |
| Ccdc96 | -1.70044184 | 1.585061927 |
| Snx7 | -1.635515241 | 1.646358355 |
| Glp2r | -1.625793908 | 1.655532212 |
| Slc5a3 | -1.631798363 | 1.649438976 |
| Cetn2 | -1.664471252 | 1.607706992 |
| Vwa3a | -1.683856361 | 1.588118051 |
| Sdc1 | -1.662558449 | 1.608491578 |
| Krt9 | -1.619957156 | 1.64223881 |
| Sytl4 | -1.696134867 | 1.56377367 |
| Rspo2 | -1.589479872 | 1.669566923 |
| Casc4 | -1.587635618 | 1.670959057 |
| Esm1 | -1.591142196 | 1.664567212 |
| Nek5 | -1.63044639 | 1.624523605 |
| Tubb6 | -1.69179198 | 1.562932254 |
| Scn11a | -1.648847296 | 1.602125596 |
| F2rl2 | -1.729128291 | 1.521176656 |
| Prl | -1.597508512 | 1.651298695 |
| Tac2 | -1.651546624 | 1.5965062 |
| Recql | -1.687089999 | 1.557984221 |
| Krt18 | -1.620105905 | 1.621787577 |
| Zkscan3 | -1.604198801 | 1.633839359 |
| H2-Q2 | -1.639304939 | 1.597286237 |
| B230399E16Rik | -1.609633236 | 1.626894758 |
| Prpsap2 | -1.56613701 | 1.662767016 |
| LOC435023 | -1.689865794 | 1.53811245 |
| Irak1 | -1.614577303 | 1.610741004 |
| Wdr69 | -1.671543552 | 1.547973067 |
| Gda | -1.649948121 | 1.567835655 |
| Kcnh6 | -1.658909668 | 1.55686805 |
| Pex10 | -1.641926412 | 1.56531477 |
| 2310061C15Rik | -1.663564247 | 1.538400683 |
| Plekha3 | -1.615137167 | 1.586673164 |
| Six6 | -1.679504668 | 1.521756804 |
| Tifab | -1.677549758 | 1.522837614 |
| Mfng | -1.593012436 | 1.607096703 |
| Casc1 | -1.601166457 | 1.59851448 |
| Higd1a | -1.546919822 | 1.646661174 |
| Nek6 | -1.66986204 | 1.523252985 |
| Actg2 | -1.645726203 | 1.546210425 |
| Hmgcll1 | -1.527244695 | 1.663139017 |
| Fank1 | -1.63068053 | 1.555710702 |
| Nmu | -1.65264674 | 1.53287651 |
| Fbxl13 | -1.648433721 | 1.536474977 |
| Ksr1 | -1.664018582 | 1.51583802 |
| BC040758 | -1.648754725 | 1.530638328 |
| Ott | -1.642250214 | 1.53525138 |
| Prl7a1 | -1.651449018 | 1.524258198 |
| Tbpl1 | -1.56051518 | 1.611965322 |
| Slc22a21 | -1.529443907 | 1.642660248 |
| Irgc1 | -1.597055648 | 1.571551227 |
| Spdef | -1.623158343 | 1.542110003 |
| C030019I05Rik | -1.638133954 | 1.527016473 |
| C1ql3 | -1.552943736 | 1.608436623 |
| Parvg | -1.614372739 | 1.543939687 |
| LOC671453 | -1.604899081 | 1.552338776 |
| Vgll2 | -1.63017683 | 1.524483446 |
| 1700027A23Rik | -1.526582042 | 1.625344188 |
| Nlrp6 | -1.643148973 | 1.508159844 |
| Chia | -1.526330416 | 1.623284049 |
| Pp11r | -1.638488073 | 1.509500039 |
| Defb1 | -1.623107973 | 1.521879579 |
| Capn11 | -1.629844716 | 1.513940332 |
| Rnf12 | -1.555652171 | 1.581689352 |
| Ptpn22 | -1.621853881 | 1.515398286 |
| Gria4 | -1.524292782 | 1.611251544 |
| Smc3 | -1.536464659 | 1.598928534 |
| 5330439C02Rik | -1.62468533 | 1.508903619 |
| Wdr3 | -1.553108511 | 1.580230961 |
| 9130008F23Rik | -1.581730307 | 1.547017614 |
| Mst1r | -1.606974311 | 1.52160864 |
| Gtl3 | -1.565643013 | 1.558896292 |
| Corin | -1.600229805 | 1.522735222 |
| Klk1b22 | -1.501464538 | 1.615708843 |
| Pmpcb | -1.52579559 | 1.590438303 |
| Cxcr4 | -1.529434926 | 1.586240211 |
| Olfr373 | -1.583346291 | 1.529185561 |
| Emr1 | -1.538082044 | 1.573074982 |
| Sp9 | -1.577703552 | 1.532354692 |
| Dkk1 | -1.562963795 | 1.54707709 |
| Pigy | -1.532409714 | 1.576132075 |
| Pik3cg | -1.54303314 | 1.565093663 |
| Slc23a3 | -1.527610214 | 1.579637974 |
| Pcf11 | -1.584189392 | 1.520454828 |
| Efcab3 | -1.563568853 | 1.538351659 |
| Cdc123 | -1.515993927 | 1.585582027 |
| Abca8b | -1.564788897 | 1.534752973 |
| Pam | -1.560391182 | 1.53707337 |
| Prpf39 | -1.504964622 | 1.592130003 |
| Folr1 | -1.535836878 | 1.559452265 |
| 4833442J19Rik | -1.545049596 | 1.547397091 |
| Tjp1 | -1.550102468 | 1.535127093 |
| Cct6b | -1.56173245 | 1.522750039 |
| Tnr | -1.572003787 | 1.512121552 |
| 1700020O03Rik | -1.53630339 | 1.543819001 |
| Tmem48 | -1.503839248 | 1.576243592 |
| 1700095K08Rik | -1.55439607 | 1.524686049 |
| Ltbp1 | -1.530408598 | 1.546274643 |
| Olfr495 | -1.548084068 | 1.528044111 |
| Npy2r | -1.5368005 | 1.537719246 |
| Inmt | -1.568204085 | 1.502607574 |
| Lhx2 | -1.508433513 | 1.560368224 |
| Prickle3 | -1.552207933 | 1.508393563 |
| 4732456N10Rik | -1.522365466 | 1.534414312 |
| Cage1 | -1.526575175 | 1.526913438 |
| 2900064A13Rik | -1.517889049 | 1.533974977 |
| Them5 | -1.534344806 | 1.50830148 |
| 4930430E16Rik | -1.521768027 | 1.518515559 |
| Pdcd1 | -1.510200153 | 1.527186854 |
| Il16 | -1.521435753 | 1.514631332 |
| D830046C22Rik | -1.515934875 | 1.518992931 |
| Tlr7 | -1.522848115 | 1.505112733 |
| Zfp93 | -1.517877572 | 1.509276363 |
| LOC100045044 | -1.502406415 | 1.513391041 |
| 2610016F04Rik | -1.506665927 | 1.506442116 |

**Table S8.** Hypothalamic gene transcripts commonly regulated by CR in WT and *db/db* mice (n=3 animals per group). Statistical significance was considered at p≤0.05.

| **Common Gene symbol** | **WT z ratio** | ***db/db* z ratio** |
| --- | --- | --- |
| Mt2 | 4.026650095 | 1.528678444 |
| Prrc1 | 2.964434787 | 1.80087088 |
| Dnm1 | 2.912346195 | 2.168688489 |
| Itgb3bp | 2.235547031 | 1.788920181 |
| Ecel1 | 2.012888613 | 1.876236849 |
|  |  |  |
| BC006662 | 1.568110509 | 2.134685945 |
|  |  |  |
| Mpp5 | -5.434475724 | -2.342686984 |
| 1810009N02Rik | -4.419038534 | -1.587861023 |
| Mrpl52 | -3.959416941 | -1.751833068 |
| Aldh1a1 | -4.549638643 | -2.653857799 |
| Enoph1 | -3.605073133 | -1.905057091 |
| Katnal1 | -3.935832573 | -2.414758303 |
| Hspa12b | -3.008145218 | -1.631560743 |
| Map1lc3b | -3.561642574 | -2.346218319 |
| Sec11c | -2.5504678 | -1.651924377 |
| Chl1 | -3.073387432 | -2.228873582 |
| Ric8b | -2.390103915 | -1.555014384 |
| Golim4 | -2.609441188 | -1.83327734 |
| 2700055A20Rik | -2.585985309 | -1.921956583 |
| Cbwd1 | -2.402653259 | -1.781744025 |
| Qtrt1 | -2.194533325 | -1.577744981 |
| Cpeb3 | -2.317195263 | -1.741682008 |
| Alcam | -2.523953653 | -1.961570009 |
| Gatad2b | -2.463470166 | -1.9350446 |
| Hexb | -2.47280719 | -2.038278966 |
| MGC41689 | -2.275114584 | -1.875570546 |
| Phkb | -2.208175912 | -1.823908702 |
| H2-Eb1 | -2.041968514 | -1.688574484 |
| C130038G02Rik | -1.952179679 | -1.605106133 |
| St6galnac2 | -1.825109273 | -1.547872751 |
| Wdr78 | -1.906875518 | -1.716844088 |
| Skp1a | -1.668750991 | -1.564168392 |
| Tesc | -1.840796523 | -1.774189039 |
| Bckdhb | -2.009425541 | -1.952993804 |
|  |  |  |
| Gosr1 | -1.513946013 | -1.574352617 |
| Prpf38b | -1.52530405 | -1.795011538 |
| Ptprb | -2.06604405 | -2.337776585 |
| Ucn | -2.518379155 | -2.819492831 |
| Ube2a | -1.583968718 | -1.914291602 |
| Mina | -1.657230526 | -2.025011658 |
| Plcb4 | -1.734688822 | -2.119896191 |
| Chchd7 | -1.628288842 | -2.054615433 |
| Osbpl6 | -3.124429111 | -3.59360283 |
| Pcyt1a | -1.622466591 | -2.256631425 |
| Creg2 | -1.713349788 | -2.496233067 |
| Zfp91-cntf | -2.030927257 | -3.246119679 |
| Ghitm | -1.661675368 | -3.117192622 |
| Ccl21c | -1.912429532 | -3.508327944 |
| Bach1 | -1.834323648 | -3.491333484 |
| Lgi2 | -1.954263793 | -3.782254273 |
| Trim11 | -1.541878285 | -3.489710612 |
| Prkag2 | -1.727790139 | -4.766779975 |
| Cit | -2.187286722 | -5.359151204 |
| Zbtb7a | -2.485031587 | -6.595648903 |
|  |  |  |
| Slc22a4 | 6.057703137 | -4.149752568 |
| Rpe | 3.771310174 | -4.063881349 |
| Ndufb2 | 4.962227606 | -1.837313568 |
| Kitl | 3.322258942 | -3.397140403 |
| Nfatc3 | 4.736191899 | -1.953597041 |
| Serpinb1a | 4.013422063 | -2.621454357 |
| EG624866 | 4.93382237 | -1.681637422 |
| Msi2h | 3.647558747 | -2.73055554 |
| Parp8 | 2.732296159 | -3.451189692 |
| Pdcd4 | 2.582791299 | -3.034218857 |
| Rnase1 | 3.546840728 | -2.059811591 |
| Zfp106 | 3.314105821 | -2.166632219 |
| 1500015O10Rik | 2.841767801 | -2.376732762 |
| Vapa | 2.939266452 | -2.179575355 |
| Kcnj12 | 2.535843936 | -2.417147217 |
| Glipr2 | 3.363038368 | -1.58844416 |
| Trim37 | 1.580577359 | -3.351092838 |
| Tpcn2 | 2.724379788 | -2.204337597 |
| Slc7a14 | 1.535216132 | -3.308293338 |
| Ncam2 | 2.251015292 | -2.493327586 |
| Fbxl18 | 1.716621999 | -3.003173254 |
| Nsbp1 | 2.3416338 | -2.11476512 |
| Parp14 | 1.980617142 | -2.301095298 |
| Sparcl1 | 2.139627492 | -2.129762236 |
| Lag3 | 2.230085154 | -2.023392021 |
| R3hdm2 | 2.336026376 | -1.91561826 |
| Rasgrp3 | 1.569247516 | -2.348790073 |
| Zfp553 | 2.360936793 | -1.500992786 |
| Cdkal1 | 1.8844009 | -1.961795643 |
| Nsg1 | 2.038753017 | -1.794338348 |
| 1190005P17Rik | 1.897422753 | -1.874957284 |
| Dlg4 | 1.579329247 | -2.053650935 |
| Ninj2 | 1.590355616 | -1.938349872 |
| Klk8 | 1.837527717 | -1.520645246 |
| Edg2 | 1.733212852 | -1.585376645 |
| Usp45 | 1.612768584 | -1.675419545 |
| Ptcd3 | 1.69102414 | -1.501613926 |
| Sltm | 1.619458705 | -1.564350638 |
| D10627 | 1.631920782 | -1.522663812 |
| Paip2 | 1.529708888 | -1.510808969 |
|  |  |  |
| Rab5b | -6.756666656 | 1.845145932 |
| Paip1 | -2.410853616 | 5.433483209 |
| D11Moh35 | -4.452925389 | 2.267435418 |
| Cacng7 | -3.505534426 | 3.114384219 |
| Acyp2 | -4.89327892 | 1.608844874 |
| C530028O21Rik | -4.678819498 | 1.736837758 |
| Cfl1 | -2.900091365 | 3.38580546 |
| Npcd | -3.423973878 | 2.427608878 |
| Paqr6 | -3.65886637 | 1.839211023 |
| Cabp7 | -3.250353783 | 1.967509621 |
| Kcnip4 | -3.102216362 | 2.101830866 |
| Cds2 | -2.874722078 | 2.257264151 |
| Ccdc115 | -3.514094262 | 1.525826789 |
| Lmtk3 | -3.405151689 | 1.603807303 |
| Nanos2 | -3.333930219 | 1.64855404 |
| Polr3e | -2.075063345 | 2.8919732 |
| Gpx2 | -3.012756211 | 1.61392093 |
| Madd | -3.027433015 | 1.567440073 |
| Nfkbib | -2.620544648 | 1.894091879 |
| Ndufb10 | -2.103002905 | 2.331174334 |
| Epn1 | -2.898687411 | 1.52704468 |
| Ralgds | -2.336983343 | 2.080189164 |
| Tnpo2 | -2.714252768 | 1.697218643 |
| Brsk1 | -2.019660457 | 2.358640407 |
| Tapbp | -2.611241451 | 1.7023718 |
| Rwdd3 | -1.814861165 | 2.334844512 |
| A330049M08Rik | -2.268298339 | 1.860499751 |
| Pde4a | -2.18664076 | 1.778673718 |
| Il18bp | -2.397441158 | 1.543116033 |
| Cited2 | -1.937295043 | 1.981290208 |
| Dnajb4 | -2.25430806 | 1.505388312 |
| Ptprn2 | -1.676697652 | 2.072439827 |
| Plekhj1 | -1.919236632 | 1.549354122 |
